# Protein Topology Determines Cysteine Oxidation Fate: The Case of Sulfenyl Amide Formation among Protein Families

**DOI:** 10.1371/journal.pcbi.1004051

**Published:** 2015-03-05

**Authors:** Lucas A. Defelipe, Esteban Lanzarotti, Diego Gauto, Marcelo A. Marti, Adrián G. Turjanski

**Affiliations:** 1 Departamento de Química Biológica, Facultad de Ciencias Exactas y Naturales, Universidad de Buenos Aires, Buenos Aires, Argentina; 2 INQUIMAE/UBA-CONICET, Facultad de Ciencias Exactas y Naturales, Universidad de Buenos Aires. Buenos Aires, Argentina; Heidelberg Institute for Theoretical Studies (HITS gGmbH), GERMANY

## Abstract

Cysteine residues have a rich chemistry and play a critical role in the catalytic activity of a plethora of enzymes. However, cysteines are susceptible to oxidation by Reactive Oxygen and Nitrogen Species, leading to a loss of their catalytic function. Therefore, cysteine oxidation is emerging as a relevant physiological regulatory mechanism. Formation of a cyclic sulfenyl amide residue at the active site of redox-regulated proteins has been proposed as a protection mechanism against irreversible oxidation as the sulfenyl amide intermediate has been identified in several proteins. However, how and why only some specific cysteine residues in particular proteins react to form this intermediate is still unknown. In the present work using in-silico based tools, we have identified a constrained conformation that accelerates sulfenyl amide formation. By means of combined MD and QM/MM calculation we show that this conformation positions the NH backbone towards the sulfenic acid and promotes the reaction to yield the sulfenyl amide intermediate, in one step with the concomitant release of a water molecule. Moreover, in a large subset of the proteins we found a conserved beta sheet-loop-helix motif, which is present across different protein folds, that is key for sulfenyl amide production as it promotes the previous formation of sulfenic acid. For catalytic activity, in several cases, proteins need the Cysteine to be in the cysteinate form, i.e. a low pK_a_ Cys. We found that the conserved motif stabilizes the cysteinate by hydrogen bonding to several NH backbone moieties. As cysteinate is also more reactive toward ROS we propose that the sheet-loop-helix motif and the constraint conformation have been selected by evolution for proteins that need a reactive Cys protected from irreversible oxidation. Our results also highlight how fold conservation can be correlated to redox chemistry regulation of protein function.

## Author Summary

Cysteine oxidation is emerging as a relevant regulatory mechanism of enzymatic function in the cell. Many proteins are protected from over oxidation by reactive oxygen species by the formation of a cyclic sulfenyl amide. Understanding how cyclic sulfenyl amide is formed and its dependence on protein structure is not only a basic question but necessary to predict which proteins may auto protect from over oxidation We describe a structural motif, which includes cysteine residues with a constrained conformation in a “forbidden” region of the Ramachandran plot plus a Beta-Cys-loop-helix motif, which has a reactive low pK_a_ Cysteine and also enables to form the cyclic sulfenyl amide with a low activation barrier. Our QM/MM computations show that the cyclization reaction only occurs if the “forbidden” conformation is acquired by the Cysteine residue. This structural motif was identified at least in 7 PFAM families and 145 proteins with solved structure, showing that a large number of proteins could have the ability to go through such cyclic product preventing irreversible oxidation.

## Introduction

**Figure 1:**
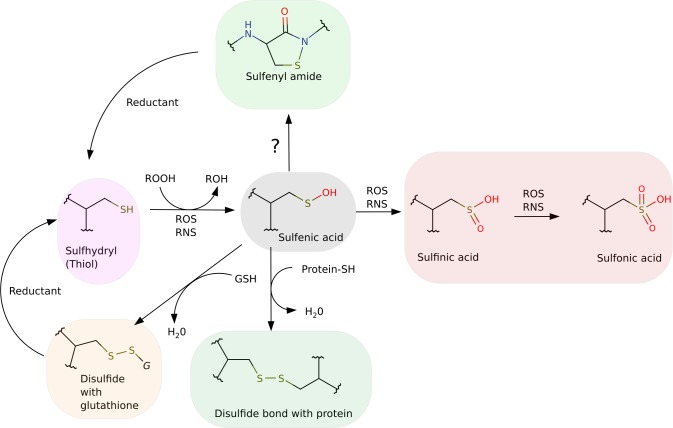
Potential reactions between protein cysteine residues, small redox molecules and RNOS.

Cysteine residues are involved in a plethora of roles in proteins, particularly in the context of cellular signaling, substrate and metal binding, protein–protein interactions and enzymatic activity. [1–4] However, most reactive cysteine residues are also quite sensitive to oxidative modification, leading to the formation of a diverse set of oxidized products when exposed to Reactive Nitrogen and/or Oxygen Species (RNOS) [1,2,5]. In this sense, oxidation is becoming an important regulatory mechanism in many proteins, and reactive cysteine residues are emerging as critical components in redox signaling [5]. A particularly oxidized form of cysteine, the sulfenic acid (Cys-SOH), which has an important role as a sensor of oxidative and nitrosative stress in enzymes and transcriptional regulators, has a rich chemistry that can modulate the fate of protein activity. Sulfenic acid is a metastable oxidized form of Cysteine, which easily gives rise to more stable products like disulfides, sulfinic acid, or even sulfonic acids i.e., overoxidation products [1,5]. Since reactive Cys oxidation usually leads to a loss of catalytic activity, there are several mechanisms that recover the reduced cysteine. These processes can be dependent on other proteins, small redox molecules (like Glutathione), or they can even occur by an autorecovery mechanism promoted by the protein itself. One autorecovery mechanism depends on the formation of an intramolecular sulfenyl amide, a cyclic product that involves the reaction of the sulfur atom with the backbone NH moiety of the succeeding residue protecting it from overoxidation. Other autorecovery mechanisms involve the reduction of the oxidized cysteine with a nearby cysteine residue to produce a disulfide bond [6]. All these possible Cys oxidation/reduction reactions are shown in Fig. 1.

The sulfenyl amide intermediate was first observed in the crystal structure of human protein tyrosine phosphatase 1B (PTP1B) [7]. Protein tyrosine phosphatases regulate signal transduction pathways involving tyrosine phosphorylation and have been implicated in the development of hypertension [8,9], diabetes [10–12], rheumatoid arthritis [13–16] and cancer [17–21]. Increasing evidence suggests that the cellular redox states of the catalytic cysteine are involved in determining tyrosine phosphatase activity through the reversible oxidization of the reactive cysteine to sulphenic acid (Cys-SOH) [18,22–24]. Hydrogen peroxide (H_2_O_2_) can regulate cellular processes by the transient inhibition of protein tyrosine phosphatases through the reversible oxidization of their catalytic cysteine, which suppresses protein dephosphorylation [25–27]. In this sense, the discovery of sulfenyl amide formation in PTP1B emerged as a possible mechanism to recover a functional reduced cysteine.

Since the discovery of the formation of the sulfenyl amide intermediate in PTP1B [7], several other proteins have been identified to harbor this intermediate, thus showing that sulfenyl amide formation is emerging as a common post-translational modification, related to protein redox reactivity. For example, in *Bacillus subtilis* OhrR, it is involved in the control of peroxiredoxins expression in response to ROS. Cyclic sulfenyl amide prevents the overoxidation of this repressor, and acts as a slow switch to prevent DNA binding, allowing the transcription of the peroxiredoxin genes [28,29]. Another protein where cyclic sulfenyl amide was detected is PTPalpha, composed by two domains, one proximal (D1), which has phosphatase activity and one distal (D2), which is not directly involved in phosphatase activity. The cyclic sulfenyl amide has been described in the D2 distal domain; in this case it functions as an allosteric regulator of the D1 domain, controlling its catalytic activity [24]. From another perspective, cysteine residues are highly reactive towards RNOS and several works have shown that protein environment regulates this reactivity by controlling not only the interaction with the oxidative species, but also by modifying for example the pK_a_ of the thiol group [30,31]. Interestingly, once the first oxidation has occurred yielding the corresponding sulfenic acid, there is little information about the molecular determinants that regulate its fate. The oxidized cysteine can follow one of two possible paths; to form a cyclic sulfenyl amide, which can then be recovered; or to be irreversibly oxidized to sulfinic or sulfonic acid. (Fig. 1) The reaction of sulfenic acid to form sulfenyl amide has been previously studied in model compounds suggesting that electronic effects are relevant but they report a high energy barrier. [32,33]

In the present work we have studied the key structural and chemical features that allow sulfenic acid to form the sulfenyl amide intermediate in proteins using *in silico* based tools. Our results show that: I) a specific and conserved beta-sheet-loop-helix motif is present across different protein folds, and positions the NH backbone, which reacts with the cysteine sulfur atom to yield the sulfenyl amide, in a constrained conformation required for the chemical reaction to occur. II) The intramolecular reaction occurs, in a concerted fashion, with S-OH bond breakage as the rate limiting process. III) Protein families having the constrained cysteines motif are reported to be involved in redox related process, strongly suggesting a functional relationship. IV) A database search for the motif shows its presence in other proteins, like the protein tyrosine phosphatase B from *Mycobacterium tuberculosis*, suggesting their possible role in redox related signaling.

## Materials and Methods

### Protein structure selection, search parameters and Cys environment characterization

In order to work with all available structures deposited in the Protein Data Bank, a relational database was built using MySQL [34] as the backend. This database stores information such as the UniProt ID, PFAM family (computed by HMMer [35]), primary, secondary and tertiary structural data like protein sequence, secondary structure (such as Alpha Helix or Beta sheet, computed by DSSP [36]), aminoacid-aminoacid contacts and phi/psi dihedral angles for every aminoacid. All these data can be used as search parameters in the database. For the current analysis, all unique proteins (as defined by UniProt ID [37] with a structure deposited in the PDB [38]) were considered. The use of UniProt Id significantly reduces the redundancy of the PDB but does not eliminate the bias due to differential representation of protein families or multiple structures of highly similar proteins. For this reason we applied also a sequence similarity filter, i.e., we considered only one structure for all sequences with over 95% identity. The total number of structures used in our study turns out to be 18,547. We removed all entries corresponding to short peptides, fully unstructured regions or those near to unresolved zones in the X-ray structure. NMR structures were selected only when the conformation was represented in at least 50% of the reported conformational modes. For the evaluation cysteine conformation, all crystal structures depicted above were filtered considering only proteins that have a cysteine residue whose psi dihedral angle is between -150 and -90 degrees (i.e. in the forbidden psi conformation). We also filtered crystals in which the constrained cysteine is involved in disulfide bonds and crystal structures with resolution of 2.5 Å or higher. This protein selection pipeline is described in S1 Fig.

**Figure 2:**
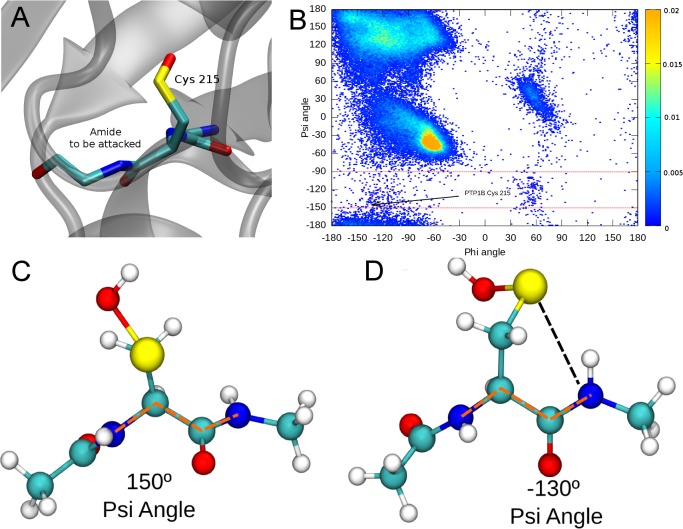
Cysteine psi conformation across the PDB. (A) PTP1B cysteine 215 in the forbidden-psi conformation, (B) Ramachandran plot of all the cysteine residues deposited in PDB using a frequency color code going from low (blue) to high frequency (orange) and the cysteines in the forbidden-psi conformation delimited by 2 red lines at -90 and -150 psi dihedral angle. Model peptide in (C) typical beta sheet conformation (angle shown in dashed orange lines) and (D) psi forbidden angle (also shown in dashed orange lines).

We found, and visually analyzed case by case, 145 proteins showing the presence of a Cys in the forbidden-psi conformation and the helix-beta-loop-helix motif. The schematic representation of each family's secondary structure, shown in Fig. 2, has been taken from the PDBsum website and done with HERA—A [39]. Analysis of secondary structure around Cys was performed using DSSP[36]. Tertiary structure analysis was performed by directly computing several structural parameters from the corresponding PBDs. In order to analyze the contribution of primary, secondary or tertiary structure to the stabilization of the Cys forbidden-psi conformation we performed two different strategies due to the fact that involved proteins belong to different families and are dissimilar in their sequences. A first approach was done by multiple sequence alignments (MSA) that were built harboring the Cysteine residue with 20 flanking residues at either side of the Cys. As a second approach, a structural alignment was computed with the whole secondary structure motif from each PDB structure, using the SALIGN algorithm [40]. The protein sequence for both alignments was done by choosing only one centroid sequence along a 95% clustering computed using CD-HIT [41]. Hidden Markov Models (HMM) were built using previously mentioned MSAs with HMMMER [42]. Each HMM was tested for their capacity to detect proteins harboring the forbidden-psi Cys. For visual analysis, HMM and frequency logos were built using Skylign [43].

**Figure 3:**
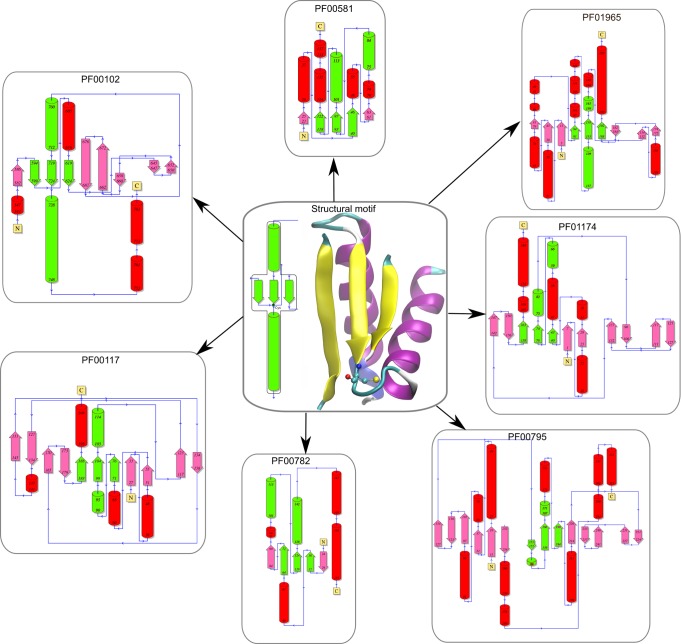
Protein topology for each relevant Pfam family (in parenthesis the PDBid of the corresponding protein) and protein fold. PF00102 (1P15), PF00117 (2VPI), PF00581 (3D1P), PF00782 (1D5R), PF00795 (2PLQ), PF01174 (2YWJ) and PF01965 (1PDW)

**Molecular Dynamics Simulations (MD)**. The starting structure for the MD simulations was retrieved from the Protein Data Bank [38], corresponding to PTP1B with Cys215 in the sulfenic acid state, (PDBid 1OET) with a crystal resolution of 2.3 Å. Using this structure three different systems were built varying in the protonation state of Histidine 214, which was either protonated in the delta Nitrogen (HID state), in the epsilon Nitrogen (HIE state) or in both nitrogens, resulting in a positively charged Histidine residue (HIP state). Standard protonation states were assigned to all other titrable residues, D and Q were negatively charged, K and R positively charged and Histidine protonation was assigned favoring formation of hydrogen bonds in the crystal structure, but in the case of the already mentioned Histidine 214. For the peptide simulations we built a small molecule containing the sulfenic acid and both the anterior and posterior peptide bonds, capped with acetamide (ACE) and N-methyl (NME) groups respectively, consisting of 24 atoms (See Fig. 3C).

For all residues, except the sulfenic acid, the AMBER99SB force field was used [44,45]. Sulfenic acid force field parameters were built using AMBER recommended procedure. Briefly, an electronic structure calculation using the HF/6–31G* method was performed, and partial atomic charges were subsequently derived using RESP procedure[46]. All bonded and VdW parameters were taken from the General AMBER Force Field[47]. Parameters for the resulting cysteine displaying a sulfenic acid side chain are shown in S2 Fig. Each protein was then immersed in a truncated octahedral box of TIP3P water consisting in 8,776 water molecules, which corresponds to a 10 Å distance between the protein surface and the box boundary [48]. Each system was optimized using a conjugate gradient algorithm for 2000 steps. This optimization was followed by 100 ps long constant volume MD, where the temperature of the system was slowly raised to 300 K. The heating was followed by a 100 ps long constant temperature and constant pressure MD simulation to equilibrate the system's density. During these processes the protein Cα atoms were restrained by a 1 kcal/mol harmonic restraint potential. Pressure and temperature were kept constant with the Berendsen barostat and thermostat respectively adjusting pressure every 1 ps. and temperature every 2 ps, using the Amber suggested default parameters. [49] All simulations were performed with periodic boundary conditions[50] using the SHAKE algorithm[51] to keep hydrogens at equilibrium bond lengths, and using a 2fs time step. Production simulations consisted in 10 ns long NTP simulations for the protein, and 100ns for the model peptide. Ewald sums were used to treat long range electrostatics, using AMBER default parameters, and with a 10 Å cutoff for direct interactions

**Constant pH molecular dynamics (CpHMD)**. For constant pH molecular dynamics, unless explicitly stated, simulation parameters were the same as detailed above. A detailed description of the parameters is presented in the original paper of CpHMD simulations [52]. Simulations were done with the Generalized Born implicit solvent representation [53]. A cutoff of 1000 Å was used for direct interactions. Temperature was kept constant (300 K) using Langevin dynamics with a collision frequency of 2.0 fs^-1^. [54] Each CpHMD simulation consisted of 10ns. In order to compute the pK_a,_ fitting to the Henderson-Hasselbalch equation was performed using non linear least square algorithm as implemented in R 3.0 package. [55].

## QM/MM methods

Determination of the reaction free energy profile using QM(DFTB)/MM and Multiple steered molecular dynamics (MSMD) strategy.

**MSMD strategy**. To determine the free energy of the reaction we used the MSMD method [56,57] which allows to link non equilibrium pulling trajectories with equilibrium properties like the free energy, and has been extensively used in our group to determine free energy profiles[57–60]. Briefly, H(r,λ) is the Hamiltonian of a system that is subject to an external time-dependent potential (λ = λ(t)). ∆G(λ) and W(λ) are the change in free energy and the external work performed on the system as it evolves from λ = λ_0_ to λ, respectively. The external work is performed by the guiding or steering force. Here r depicts a configuration of the whole system, while λ is the reaction coordinate. Then, ∆G(λ) and W(λ) are related to each other by the following equation, known as Jarzynski's relationship [56]

$$e^{-\beta \Delta G(\lambda )}=<e^{-\beta w(\lambda )}>$$ (1)

The brackets in equation (1) represent an average taken over an ensemble of molecular dynamics trajectories provided the initial ensemble is equilibrated. Thus, in practice, in order to obtain ∆G(λ), multiple trajectories are performed were the system is steered from reactants to products along λ, using an external force (which usually takes a harmonic potential form) and the work (λ) performed is measured along the trajectory. Once several trajectories and the corresponding work (λ) profiles have been determined, the free energy profile G(λ) is obtained using equation (1).

In order to perform each trajectory, equilibrated snapshots were taken from classical Molecular Dynamics simulations of the reactant state and used as starting point for the QM/MM steering simulations. In each case, systems were first optimized for 5000 steps and gently thermalized to 300 K for 50 ps. Another 50ps were done to allow equilibration of the system density while temperature was kept constant using Langevin thermostat [61] with a collision frequency of 5 ps^-1^, and pressure was adjusted using Berendsen barostat every picosecond. Finally, each production MSMD run was performed in two steps. The first step consists of the breakage of the S-OH bond (with OH leaving as a water molecule after proton transfer from H_2_O or H_3_O^+^) and the formation of the S-N bond using the following reaction coordinate

$$\lambda (r)=d(S-O_{S})-d(O_{S}-H_{H_{2}O})-d(S-N)-d(H_{N}-O_{WAT})$$ (2)

**Figure 4:**
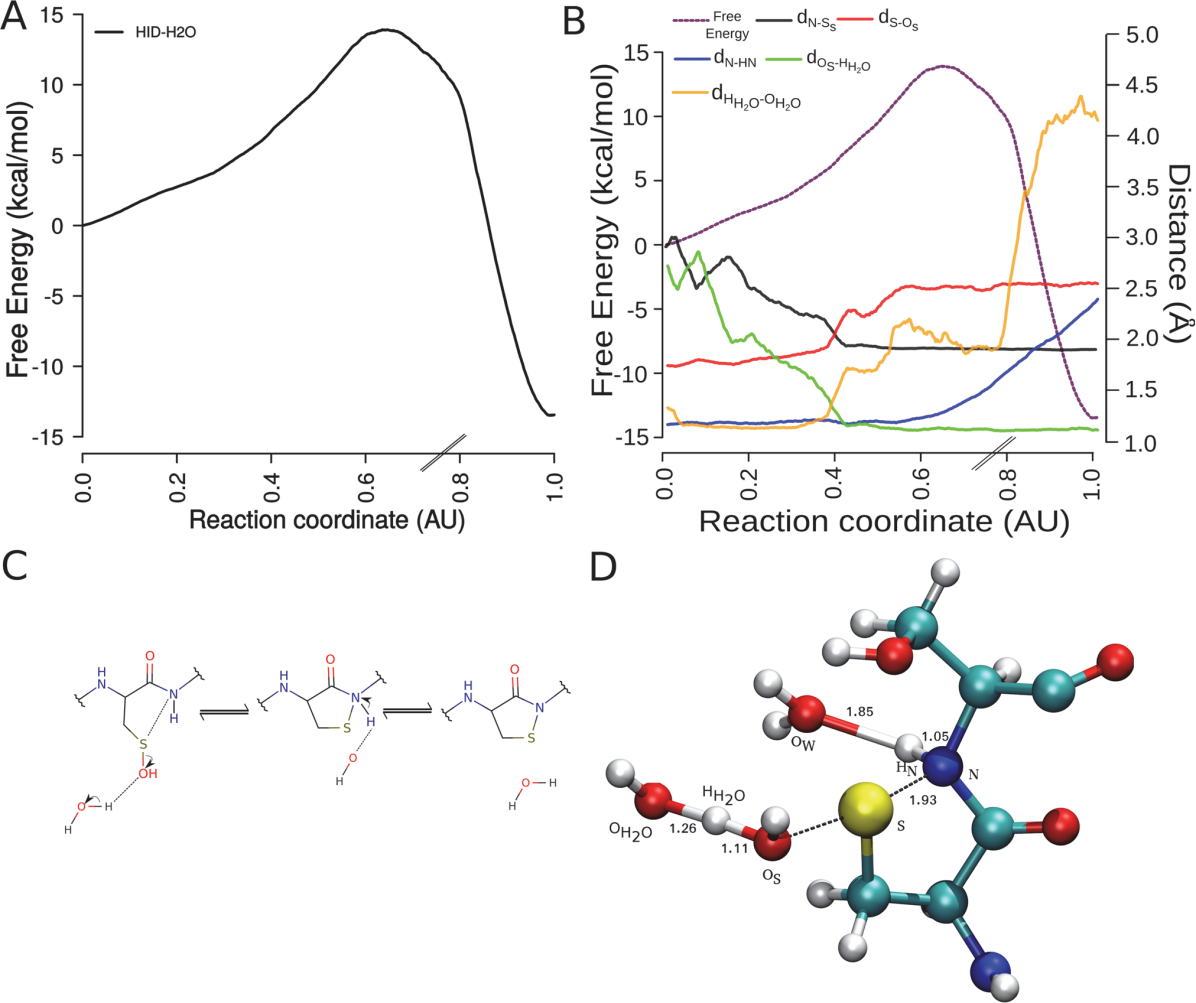
Free energy profile, distance and structural parameters of sulfenyl amide formation in PTP1B. A) Free energy profile of cyclic sulfenyl amide formation with H_2_O and His 214 in HID State (in black) (B) Running average on relevant distances for the reaction for H_2_O and His 214 in HID state plotted along free energy profile. (C) Proposed reaction scheme. Fine dashed lines on the left depict bonds to be formed. (D) Geometry of the TS1. Color code of atoms: Carbon (Cyan), Nitrogen (Blue), Oxygen (Red), Sulphur (Yellow) and Hydrogen (White)

The second step involves the transfer of the amide proton to a water molecule, regenerating the H_2_O or H_3_O^+^molecule. (Fig. 4C).

$$\lambda (r)=d(N-H_{N})-d(H_{N}-O_{WAT})$$ (3)

All simulations were performed with periodic boundary conditions [50] and a time step of 1fs. The first step steering dynamics was performed during 48ps and the second during 20ps. No link atoms were necessary for the peptide system whereas the protein had two link atoms.

**QM system and level of theory**. In the case of the peptide, the QM system comprises the whole peptide (ACE-Cys-SOH-NME), H_3_O^+^ molecule and the closest 9 water molecules to the system. For the protein, the QM system consists of His214—Cys-SOH 215—Ser216, a H_2_O or H_3_O^+^ molecule and, also, the closest 9 water molecules to the reacting atoms. We chose the Self-consistent charge density functional tight-binding (SCC-DFTB) [61] level of theory because it offers a balanced tradeoff between accuracy and computational cost and used as implemented in AMBER 12 package. [62–64] In order to test the adequacy and accuracy of the theory level we computed the energy profile of the reaction using restraint optimizations with a higher level of theory (see below).

**Restraint optimization**. To compare and determine the potential accuracy of the level of theory used to compute the free energy profiles, we determine the corresponding energy profile using restraint optimizations with the Hybrid program [65,66] which is based on the ab-initio SIESTA code working at the density functional level of theory using the generalized gradient approximation (GGA) functional proposed by Perdew, Burke, and Ernzerhof [67] and using for all atoms in the QM subsystem, basis sets of double-ζ plus polarization quality, with a pseudoatomic orbital energy shift of 25 meV and a grid cutoff of 150 Ry. The hybrid method has been extensively used to compute a diverse sample of enzymatic reaction mechanisms, showing an excellent performance [68–70]. The QM system and reaction coordinate were the same as those described above for the free energy calculations, but instead of MSDM a restraint energy minimization scheme was used. The results in S3 Fig. show that energy profile has a shape and barrier magnitude similar to those obtained with SCC-DFTB, thus justifying its choice for the analysis of the reaction mechanism.

### Statistics and graphics

Graphics were produced with R 3.0 statistical package[55]. VMD has been used to produce images of the protein or the peptide[71].

## Results

The results are organized as follows: In the first place we characterized the local sequence and structural properties of all reactive cysteines found in the PDB, and related these data with their possible implication in oxidative signaling. Secondly, we analyzed the “forbidden” conformation and finally, we determined the free energy profile of the sulfenic acid to cyclic sulfenyl amide reaction using QM/MM methods in human PTP1B.

### 1 Analyses of reactive, sulfenyl amide forming proteins

As mentioned in the introduction, PTP1B has been crystallized with Cys215 in both the oxidized, sulfenic acid state, as well as the cyclic sulfenyl amide state. A closer look at this residue sequence and structural environment shows one interesting observation. Cys215 displays, both in the reduced and sulfenic acid states, a position in the Ramachandran plot which usually constitutes a forbidden zone. This conformation results (as shown in Fig. 3A) in a configuration that orients or directs the side chain of the Cys residue in the same direction as the NH hydrogen of the Cysteine215-Serine216 peptide bond (i.e the NH of the following residue), which is the nitrogen required to form the cyclic sulfenyl amide. We will call this cysteine conformation the “forbidden-psi” conformation or constrained conformation. We also analyzed whether there are any other proteins displaying cyclic sulfenyl amide in the PDB. Apart from PTP1B, we found only one case, Phospho-2-dehydro-3-deoxyheptonate aldolase AroG from *Mycobacterium tuberculosis* (S1 Table), which seems to harbor a cyclic sulfenyl amide (S-N distance is 1.85A). In AroG the cysteine residue is located in a long unstructured loop with no clear catalytic function described. Moreover, the presence of the sulfenyl amide is not mentioned by the authors[72].

In order to analyze how often a Cysteine residue is found adopting the corresponding forbidden-psi conformation, we surveyed all PDB structures[38] and measured their psi and phi angles for all Cysteines. The resulting 2-dimensional histogram is shown in Fig. 3B. As expected, most Cys residues are found in the allowed zones (corresponding to alpha and beta secondary structures). However, there is a significant number of Cys residues displaying forbidden-psi values, i.e. falling in the zone delimited by the red lines in Fig. 3B. To characterize them further, we selected all *unique* protein structures (as defined in methods) with a Cys adopting the forbidden-psi value (corresponding to a range of psi values between -150 and -90 degrees, resulting in 270 structures (See S2 Table for a full list of the corresponding PDB entries). As an example, Fig. 3C and 3D show respectively an oxidized sulfenic acid Cys residue adopting a common beta structure conformation and the forbidden-psi conformation.

**Structural characteristics of the forbidden-psi Cys**. To analyze the structural surrounding of the relevant Cys, we used two different approaches. First, we characterized the immediate environment of the forbidden-psi Cys, by selecting all residues having at least one atom less than 8 Å away from the cysteine center of mass. However, we could not identify any over represented aminoacid (or aminoacid type) or any conserved set of interactions, not even Histidine, a residue that was proposed to form a hydrogen bond with the carbonyl group of the cysteine peptide bond in PTP1B and relevant for Cys reactivity.

Secondly, we thought about the possibility of the local protein fold being responsible for forcing the Cys to assume the forbidden-psi conformation. Strikingly, we found 53% (145/270) of the proteins displaying a forbidden-psi Cys adopt the same local fold around it, characterized by a beta strand-loop-helix secondary structural element with the relevant Cys located at the end of the beta-strand, which is also part of a parallel beta sheet motif, with at least three strands. Moreover, the Cys containing strand appeared to be always the one in the center of the three beta-strands. The corresponding fold is shown in Fig. 2.

The PFAM families were checked in order to see if the proteins having a Cys displaying the forbidden psi conformation but lacking the structural motif correspond to common protein functions. Only two families have a significant number of structures, more than three unique proteins, with a constrained cysteine: Retroviral aspartyl protease (PF00077) and Beta-lactamase2 (PF13354) (S3 Table). In the Retroviral aspartyl protease family, the cysteine in the forbidden psi conformation is in a turn between two beta sheets, thus a similar motif. Crystal structures of this protein family are generally homo-dimers, with a subunit presenting the Cys in the forbidden-psi conformation, while in the other one adopts a left handed helix conformation. Overoxidation of the cysteine residue has not been reported in this family. In the case of the Beta-lactamase2 family, many structures present a disulphide bond between the constrained Cys and a nearby Cys placed in a beta sheet. As in the case of Dual specificity phosphatases like PTEN, it is possible that disulphide bond formation is faster than sulfenyl amide formation. Overoxidation of these cysteine residues has also not been reported. All the other proteins identified with the constrained cysteine belong to families with only one structure. Taking this into account, from now on we concentrate our analysis in the 145 proteins that have the forbidden Cys and also have the same local fold.

**Sequence characteristics of the forbidden-psi Cys**. Initially, we looked for any conservation in the sequence surrounding the forbidden-psi Cys by analyzing two different length segments, one corresponding to 20 residues at each side of the Cys and another including the whole secondary structure motif harboring the Cys residue. We performed multiple sequence alignment (MSA) and built the corresponding HMM either fixing the alignment without gaps around the Cys for the short segments or performing structural alignment for the whole motif. We used the built HMMs to detect those proteins sequences harboring the forbidden-psi Cys in the whole SWISS-PROT sequence database [73]. The results are shown in S4 Fig. The fixed model is able to find 93 out of 145 proteins with the motif (64%) whereas the Structural model only recognizes 40% of the 145 proteins. Interestingly, the search also retrieves proteins (whose structure is unknown) with Phosphatase activity and GATase activity, which presumably could display a forbidden-psi Cys and/or the structural motif. The search also retrieves some false positives. Visual analysis of the HMM logo (S5 Fig.) shows some partially conserved residues like a His residue before the Cys, a rather conserved Glycine three residues ahead and an Arginine also rather conserved six residues ahead.

**Family assignment and analysis of the proteins containing the forbidden-psi Cys Fold**. Having identified a common structural fold around the forbidden-psi Cys, we looked at how this element is inserted in larger protein folds or domains. For this sake, we assigned all found structures to PFAM families[74]. Interestingly, most structures (104 out of 145 structures, 72%) with the forbidden-psi Cys are found in only seven protein families. Given that PFAM families usually define unique protein structural and functional domains, we analyzed how many of the reported structures from each family have a forbidden-psi Cys. As expected, most of the solved structures display the forbidden-psi. Remarkably, as shown in Fig. 2, global folds corresponding to the families harboring the reactive Cys are quite different, despite having the conserved forbidden-psi local fold. We identified two big families of proteins, phosphatases (with three PFAM families) and glutamine amido transferase (with two PFAM families). A structural alignment of the structural motif is shown in S6 Fig. These results are summarized in Table 1.

**Table 1:** Analyses of fold and oxidation detection in PFAM families.

Important Families	PFAM accession	% of Proteins in family	Exp Info Oxidation	Reference
Y_phosphatase	PF00102	97	Yes	[24,32]
Glutamine amidotransferase (GATase)	PF00117	100	No	
Rhodanese-like domain	PF00581	92	Yes	[75]
Dual specificity phosphatase	PF00782	95	Yes	[28,76]
Carbon-nitrogen hydrolase	PF00795	100	No	
SNO glutamine amidotransferase	PF01174	83	Yes (ortholog)	[77]
DJ-1/PfpI	PF01965	100	Yes	[77–79]

Statistical analysis of proteins in a given family (as defined by PFAM) that display the strand-loop-helix motif with the corresponding Cysteine in the forbidden-psi conformation. The last column “Exp. Info. Oxidation.” shows whether experimental information concerning Cysteine oxidation has been reported in any protein of the PFAM family.

Assignment of the forbidden-psi Cys containing proteins to families, prompted us to explore whether these proteins were reported to play a role in oxidative processes, and thus gain some insight on the likelihood that the cysteine, its sulfenic acid and/or cyclic sulfenyl amide, could be physiologically relevant. For this sake, we performed a systematic literature search for any information related to Cys oxidation in each of the relevant families reported in Table 1. Surprisingly, for five out of the seven families, we found reports relating the forbidden-psi Cys with either catalysis or a regulatory role, and a specific mention to a directly related oxidative process. (Table 1 and references therein). We now will comment on these families (Specific proteins with relevant data are presented in S4 Table):

**Protein Tyrosine Phosphatase (PF00102, Y_phosphatase)**. As commented above, PTPs are involved in a plethora of biological processes and are sensitive to oxidative stress. In this PFAM family 33 proteins have been found with the forbidden-psi Cys. The cysteine residue in the forbidden region is involved in the catalytic activity of these proteins and has been shown to be oxidized to sulfenic acid and to form cyclic sulfenyl amides (The already mentioned human PTP1B belongs to this group)[7].

**Glutamine amidotransferase (PF00117-GATase)**. Proteins from this group are involved in the transfer of the ammonia group of glutamine to an organic molecule. Detected Cysteine residues belong to the catalytic triad of these enzymes[80]. In analysis of the 18 unique proteins crystallized from this family all 18 have the constrained cysteine. Nevertheless, oxidation has not been observed in any of the crystallized proteins. In this sense, we foresee that redox agents could regulate proteins from this family, as they have the “constrained conformation”, the conserved motif, and a relatively exposed Cys residue.

**Rhodanese-like domain (PF00581)**. Members of this family include Cdc25 phosphatase catalytic domain, non-catalytic domains of eukaryotic dual-specificity MAPK-phosphatases, non-catalytic domains of yeast PTP-type MAPK-phosphatases and many bacterial cold-shock and phage-shock proteins. The cysteine residue is involved in catalysis and has been described in its oxidized state[81] (as sulfenic acid). In this case 92% crystallized proteins have the constrained cysteine.

**Dual specificity phosphatase catalytic domain (PF00782)**. These proteins are able to dephosphorylate proteins with both pTyr and pSer/pThr residues and a cysteine residue is involved in the reaction. Oxidation of the reactive cysteine has been observed in some of its members[76]. In this case, 95% proteins with crystal structure have the constrained cysteine.

**Carbon-nitrogen hydrolase (PF00795)**. These enzymes are involved in the breakage of a carbon-nitrogen bond in different compounds. Again, this group of proteins have a catalytic cysteine[82] involved in the reaction. Although oxidation of these cysteine residues has not been reported yet, all of the proteins have cysteines in the unfavorable region.

**SNO glutamine amidotransferase (PF01174)**. Members of this family are involved in the biosynthetic pathway of vitamin B6 (Pyridoxal phosphate) and are active in its hetero oligomer state. This oligomer is formed in an equimolar relationship of one amidotransferase chain (called Pdx2) and one synthase domain (called Pdx1).[83,84] Oxidation of the catalytic cysteine has been reported for pdxT from *Staphylococcus aureus*. [77] Only one member of this group does not have the cysteine contraint conformation.

**DJ-1/PfpI (PF01965)**. Proteins from this family include transcriptional regulators, proteases, chaperones and proteins with diverse roles such as DJ-1 which is involved in the development of Parkinson's disease. Because of its pathological relevance and protective role in oxidative stress DJ-1 has been intensively studied and oxidation of the active site cysteine has been described several times [78,85]. All the proteins from this group have the constrained cysteine.

In summary, global analysis of all available unique protein structures shows that there is a significant number of them harboring a Cys residue displaying a conformation with the psi angle in a forbidden region (-90° to-150° degrees), that orients the Cys side chain in the same direction as the next peptide bond NH moiety. Unexpectedly, structural domain analysis shows that the forbidden-psi Cys is in a large number of cases located in a motif consisting of a strand-(Cys)-loop-helix motif, inserted in several different global protein folds. They correspond to, at least, seven different protein families (according to PFAM) in which the Cys residue is important for catalysis and for five of these families. There have been reports on cysteine oxidation to sulfenic acid, implying that redox regulation may be associated with our findings.

**The pK**_**a**_ **of the Cys with the forbidden-psi**. Cys reactivity is tightly related to its pK_a_. In particular, Cys oxidation is promoted for those Cys with lower pK_a_s which display significant population of the charged state. Therefore, we decided to analyze whether forbid1den-psi conformation and secondary motif could affect it. We used constant pH MD simulations to determine Cys pK_a_ in both a constrained model peptide in the forbidden-psi conformation and a small peptide harboring the whole secondary structure motif taken from the crystal structure of PTP1B. The results show, as expected, that in the free peptide the reference pK_a_ for Cys is obtained (8.50). Imposing a psi angle restriction results in a slightly higher pK_a_ (9.04), a difference within the order of the error of the method which indicates that the constrained psi-conformation is not inducing a change in the pK_a_ for the Cys. Interestingly, in the case of the peptide mimicking the structural motif, the computed pK_a_ value is 4.82. Thus, it is clear that the secondary structural motif lowers the pK_a_ of the active cysteine. The extreme low pK_a_ could be an artifact which allows to take only part of a protein and to highlight the role that the local structure plays lowering the pK_a_.

We also decided to analyze Cys pK_a_ in PTP1B, which is our test case. Excitingly, CpHMD simulations show that in PTP1B the Cys protonation state is coupled to a small but significant conformational change that results in Cys displaying a conformational dependent pK_a_ yielding extreme values of 0 and 11.5. The unusually low value seems to be the result of several strong hydrogen bond interactions that the deprotonated Cys performs with the protein environment (Shown in S7 Fig.). Although in these cases obtaining the pK_a_ requires knowledge of the conformational equilibrium constant, previous experimental estimations yielded a value of 5.6 [86], which again shows that reactive Cys pK_a_ is lowered.

We now turn our attention to the chemical reaction of forbidden-psi Cys in the sulfenic acid state to yield cyclic sulfenyl amide, using human PTP1B as a test case. Our hypothesis is that the forbidden-psi conformation is directly responsible for the formation of cyclic sulfenyl amide.

### 2. Sulfenyl amide formation in model peptide and PTP 1B

**Energetic analysis of the forbidden-psi Cys conformation in a model peptide**. The results presented above highlight the relationship between the forbidden-psi conformation and the conserved beta strand-loop-helix motif with the functional relevance of Cys residues and its possible implication in redox regulation. We initially analyzed the free energy difference between the forbidden-psi conformation and allowed helix conformation. The data presented in Fig. 3B allows an estimation of how much energy proteins must pay to constraint the Cys in the reactive (forbidden-psi) conformation using the Ramachandran plot derived free energy, estimated it around 5.5 kcal/mol. We then conducted an independent estimation of the corresponding cost in the sulfenic acid form. For this sake, we built a small peptide containing a sulfenic acid oxidized cysteine capped with Acetyl and N-Methyl groups, in the N and C terminal respectively (as shown in Fig. 3C and 3D).

We then performed 100ns long MD simulations for the peptide containing Cys-OH in water. The MD results (shown in S8 Fig.), show that rotation along the psi dihedral angle has two minima, one at -30 degrees, spanning from 60° to -20° corresponding to helix like structures, and a second one with the minimum at 150°, spanning from 120° to 180° corresponding to structures in a sheet-like conformation. Interestingly, the peptide presents almost no conformations in the -60° to -160° range, during the whole simulation time scale. Free energy estimations show that the “forbidden psi conformations” are over 5 kcal/mol higher than the two minima, in agreement with the previous Ramachandran plot analysis and the results of Hornak et al for Ala tetrapeptides, where this region of the Ramachandran plot has a free energy higher than 5 kcal/mol [44]. Clearly, our results show that the protein must pay a considerable (free) energy cost to have a cysteine in the reactive or forbidden-psi structure, both in the Cys and sulfenic acid form.

Since potential SOH to backbone amide interaction could stabilize the constrained conformation, we analyzed the likelihood of internal hydrogen bond interactions between the amide hydrogen and either the sulfenic acid S or O atoms. Distances and angle measurement during the simulation show that there is not a strong interaction that could be accounted as a hydrogen bond during the simulation timescale (i.e. H_NH_-S and H_NH_-O_SOH_) distances are larger than 3.5 Å most of the time) (S3 Fig.).

**Protein environment effects on Cys conformation in PTPB1**. Environmental structural analysis revealed that there are not clear interactions around the Cys residues that could be favoring not only the constrained conformation but also the sulfenyl amide formation. However, as shown by S3 Fig. His214 (depending on its tautomeric state, see below) may establish a hydrogen bond with Cys215 carbonyl, an interaction which has been suggested to increase the partial charge on the backbone nitrogen enhancing its reactivity and supporting a nucleophilic substitution mechanism for PTP1B[87,88]. Taking this into account we decided to analyze the role of the Histidine tautomeric state. In order to analyze the cyclic sulfenyl amide reaction mechanism in PTP1B (see below) and the role played by His214 (in all possible tautomeric states) we performed 10ns long MD simulations starting from the Cys215-SOH modified PTP1B setting histidine tautomeric states either as HIE, HID or HIP (see Methods for details). The results show that the protein is stable in all three systems but significant differences are observed concerning the local structure of the Cys215 loop. S3E Fig. shows histograms for the Cys215 psi angle for all three states. As shown by the figure it is clear that Histidine protonation state affects Cys psi dihedral angle. When His214 is in the HIE state, no hydrogen bond interaction can be established and as consequence the psi angle shows values further from 180° (mean value is 126°). When His214 is simulated as HID, hydrogen bond between His214 and Cys215 carbonyl forms and breaks several times during the simulation (see S3B Fig.) with a population of ca 50%. Consequently, the Cys215 psi angle has an average value of 175°, whereas when His214 is protonated (HIP state) the His214-Cys215 hydrogen bond is present 90% of the time (See S3B Fig.) and the average psi value is -165 degrees. In order to have an estimation of each His tautomer population, we performed constant pH MD simulations using His214 as the titrable residue. The results, show that at pH = 7 HID is the most populated state, and pK_a_ is estimated to be around 4. These results show clear evidence linking the His protonation state with the Cys-SOH conformation. Being HID the most populated state at physiological pH and HIP the one which enhances the forbidden-psi conformation; we decided to perform QM/MM MSMD computations with PTP1B His 214 in both HID and HIP states

**QM/MM study of sulfenyl amide formation reaction mechanism**. In order to understand in detail the reaction mechanism of cyclic sulfenyl amide formation, we determined the corresponding free energy profile using a QM/MM strategy as explained in methods. The easiest reaction mechanism that can be envisaged requires the Cys-OH group to take a hydrogen atom or a proton from the backbone amide group of the previous residue (Ser216 in this case) to form the leaving water, leading to subsequent N-S bond formation. This mechanism has been tested in model systems by other groups [32] giving activation barriers ca. 50 kcal/mol, thus too high to account for a biological relevant process. Indeed we obtained similar values for the reaction using a model peptide in vacuum (See S9B Fig.). Therefore, we thought on possible alternative mechanisms. In proteins, the reaction occurs in water, and since the key event in the reaction seems to be the breakage of the S-O bond, we decided to test whether the presence of explicit waters in the QM system could yield smaller barriers.

To test this idea, we included in the QM system 10 water molecules and explicitly promoted proton transfer from the solvent to the S-OH group. The results (presented in Table 2 and Fig. 4) show that the presence of explicit waters is key for determining the reaction mechanism and barrier. The free energy barrier is 13.9 kcal/mol (Fig. 4A), which yields an intermediate with a broken S-O bond and a well formed S-N bond, but the N is still attached to the amide proton, thus having sp3 like character. In a second step, the amide proton is released to water, almost barrierless, yielding the cyclic sulfenyl amide product. The reaction is moderately exergonic by ca -14 kcal/mol. Distance analyses along the reaction (Fig. 4B), show that the first step occurs in a concerted fashion, as soon as the S-OH bond is broken (red line) the S-N bond forms (black line) and this process occurs simultaneously with proton transfer from the solvent to the S-OH group (green and yellow lines). The TS depicted in Fig. 4 shows a completely broken S-O bond, a well formed water molecule and the S and N atoms quite close at a distance of 1.93 angstroms. After the TS the key event is proton transfer from the NH to the solvent (blue line). During the reaction the leaving Oxygen increases its negative charge, while the NH proton slightly increases it. Also, as expected, along the reaction the psi dihedral angle does not change significantly, until the end of the reaction reaching a value of -150°.

**Table 2:** Structural and energetic parameters for the sulfenyl amide formation in PTP1B.

State	PTP1B + HID 214 + H_2_O		
ΔG	-14 kcal/mol		
ΔG^++^	13.9 kcal/mol		
	Reactive	TS	Product
N-CA-C-N+1	-155	-153	-150
dS-O_S_	1.63	1.98	2.5
dN-H_N_	0.98	1.05	2.54
dH_NH_-Ow	1.91	1.85	0.98
dO_S_-H_H2O_	2.71	1.11	0.97
dS-N	2.99	1.93	1.85
qS	-0.197	-0.187	-0.21
qO_S_	-0.244	-0.437	-0.572
qH_NH_	0.252	0.256	0.307
qN	-0.275	-0.105	-0.14
qO_W_	-0.619	-0.662	-0.54
qH_H2O_	0.451	0.382	0.356

The fact that the first and most important TS requires water release after proton transfer from the solvent, suggests that the reaction first step rate may be enhanced in acidic media. To analyze possible pH effect, we also computed the reaction free energy adding one hydronium ion hydrogen bonded to the S-OH group in the QM system. The resulting FEP and mechanistic analysis shows that reaction proceeds similarly as described above, but the barrier is slightly smaller 10.6 kcal/mol. This slight decrease in the barrier is due to the fact that transferring the proton from the hydronium ion is easier than from water. Lastly, we also computed the free energy setting His214 in the HIP state and using a hydronium ion (S10 Fig., blue curve), again mechanistic analysis shows similar results and the barrier is similar as in the previous case, thus His protonation state does not seem to affect significantly the reaction barrier. In summary, despite the second step is expected to decrease its rate when lowering the pH since the solvent must act as a base. Given the above mentioned results, and since the first barrier is significantly larger than the second, pH effects are expected to affect each barrier differently and possibly enhance the reaction rate.

In order to analyze whether the protein environment and the conformational restraining effect, we performed the reaction in a model peptide in a box of waters. Interestingly, the reaction occurs with a similar mechanism and with almost the same barrier as in the protein, but only if the peptide conformation is restrained to the forbidden-psi angle (See S9C Fig.). Trying to make the reaction to happen with Cys in a non forbidden conformation results in non reactive trajectories. As we stated in the methods section, to determine the accuracy of the level of theory used to compute the free energy profiles, we determined the reaction by using the Hybrid program [65,66]. Similar results were obtained with an activation barrier of 9kcal/mol (S9C Fig.), showing that DFTB yields good results and can be used with free energy scheme.

Analysis of the charge (Table 2) of the involved atoms during the reaction shows that most of the atoms do not have a relevant change in their atomic charge. We observe only an increase in the O_s_ atom that is due to its transfer from the sulfenic molecule to form a water molecule. There is also a slight decrease in the backbone nitrogen, as it binds the sulfur atom but keeps the hydrogen that is partially restored once the hydrogen is transferred to the solvent.

In summary, our results show that the reaction mechanism involves proton transfer from and back to the solvent, with the heterolyic breaking of the S-O bond and formation of the leaving water as a key process. The reaction has a moderate barrier and thus is expected to occur readily. Clearly, neglecting the presence of explicit waters, as in previous works [32,33] yielded barriers which are too high to be compatible with a physiological role.

**Figure 5:**
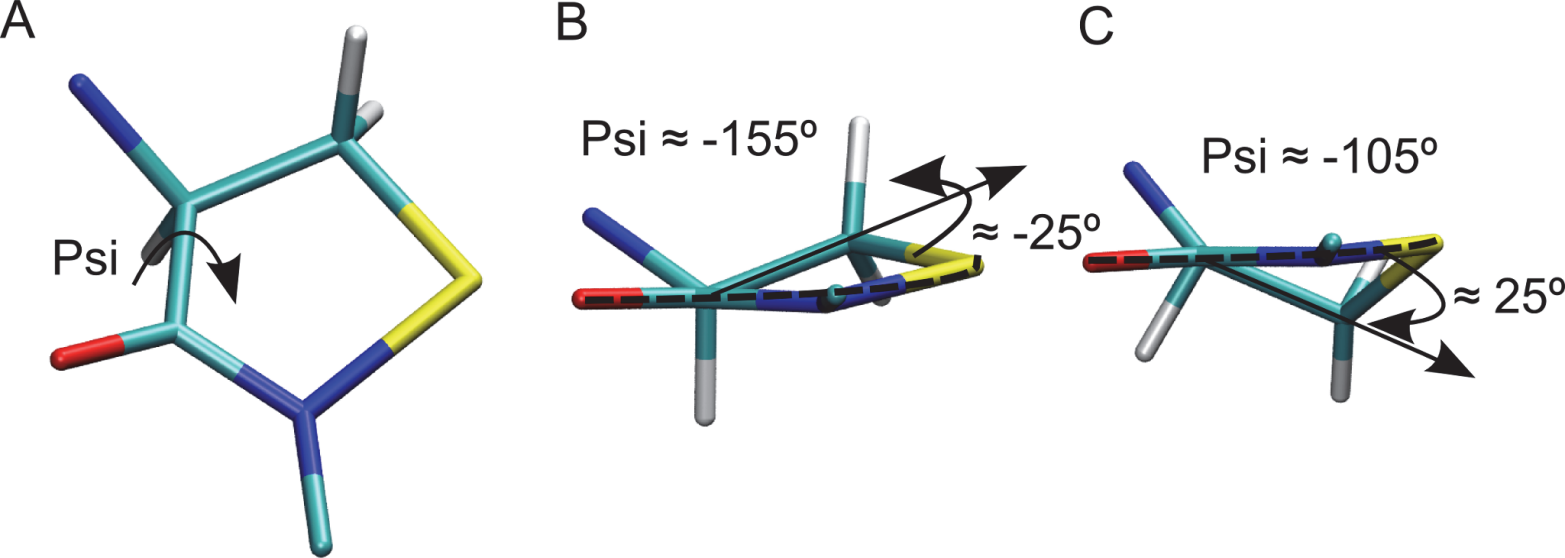
Cyclic sulfenyl amide product structure and its consequences to the reactions occurrence. A) Top view of the cyclic sulfenyl amide psi dihedral angle. (B) Lateral view of the cyclic sulfenyl amide product with a -155° psi dihedral angle. (C) Lateral view of the cyclic sulfenyl amide product with a -105° psi dihedral angle

**Product structure**. An important point should also be made concerning cyclic sulfenyl amide product structure and the Cys psi- dihedral angle. The analysis is similar to that of the phi-values of any Proline residue, due to the intra residue N-C bond. Briefly, given the non aromatic characteristic of the Cα and Cβ atoms of the five membered ring, the cyclic structure is non planar as shown in Fig. 5. As already discussed, the key parameter for the reaction is the psi angle, which involves rotation along the Cys Cα-C bond, and which in turn defines the relative orientation of the residue side chain, including Cβ. As a consequence, fixing the Cβ position in the heterocycle as in the product imposes a strong constraint in the Cys psi-angle. Our results show that using the Cys-Ser peptide bond plane as a reference, which also contains the Cys Cα (Dashed line in Fig. 5), the Cα-Cβ bond can be positioned establishing a ca. 20° to 30° angle to either side of this defined plane, as shown in Fig. 5B and C. As a result, when the angle is negative (counterclockwise) a psi angle of ca -155° is imposed to Cys, while for positive angles (Fig. 5C) the imposed Cys psi angle is ca. -105°. These results confirm that if the protein Cys cannot adopt any of the mentioned “forbidden psi” values, cyclic sulfenyl amide formation is impossible.

Taking all results together, it is clear that the reaction barrier is low, that the mechanism is clearly dissociative, and that there is no role for the protein in catalysis but to position the Cys psi angle in the constrained but reactive conformation compatible with the cyclic product structure.

## Discussion

### Protein topology and Cysteine reactivity

**Figure 6:**
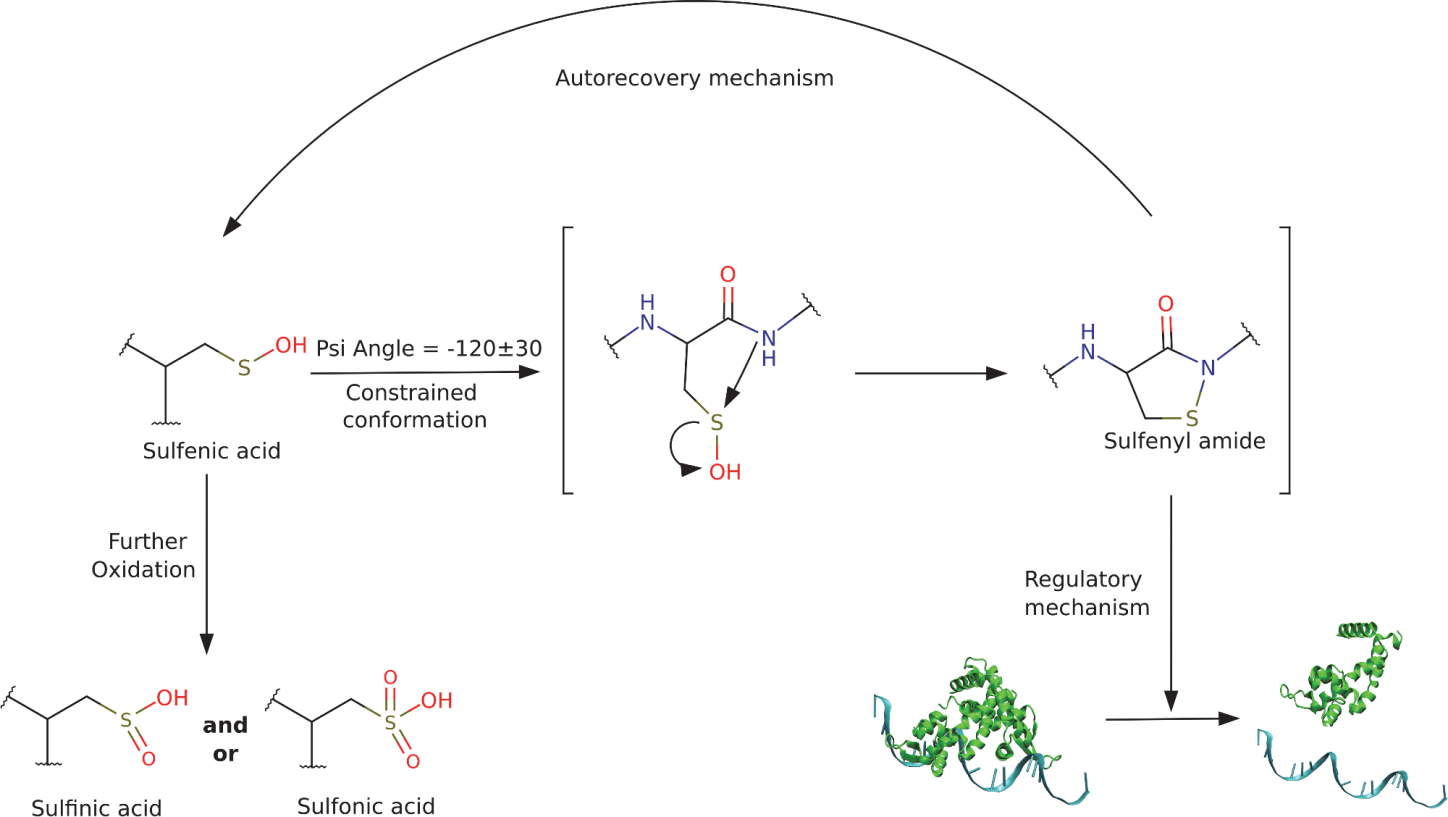
Role of cyclic sulfenyl amide formation in protein regulation and protection from overoxidation.

In this work we have shown that cysteine reactivity can be controlled by the protein topology thus acquiring a specific conformation that regulates the barrier to form cyclic sulfenyl amides. (Fig. 6) We started our analyses by identifying the presence of a Cys residue displaying a forbidden-psi angle in the -90° to -150° range in PTP1B known to form cyclic sulfenyl amide, and therefore performed a search across all protein structures found in the PDB. We were able to identify a set of protein families that have a significant number of members with the constrained cysteine that are involved in redox processes. Moreover, the identified proteins share a common topology that seems to be relevant for lowering the reactive Cysteine pK_a_ and therefore enhancing their catalytic activity. However, this also enhances Cysteine reactivity towards ROS, and inactivation of the proteins. According to our study, it seems that this motif has been selected by evolution to accelerate catalytic activity and also to protect the cysteine from further oxidation, once is oxidized to sulfenic acid, by catalyzing the formation of a cyclic sulfenyl amide that can then be recovered to cysteine.

We identified seven PFAM protein families with several members with the conserved structural motif as we pointed out before. The most important in terms of available experimental information is the protein tyrosine phosphatase family where the first cyclic sulfenyl amide was identified in the crystal structure of PTP1B[87]. This cyclic sulfenyl amide product has also been described in PTPAlpha after H_2_O_2_ treatment of the protein [24]. However, for some members of this family like the SH2 phosphatases, which have a constrained conformation and a conserved topology, some reports have detected disulfide bonds instead of sulfenyl amide [23]. Similar results have been published for Cdc25, Rhodanase-Like domain, [26], and for PTEN of the Dual specificity Phoshpatase domain[89]. Interestingly, all these proteins, and not other members of the family, have another Cys in the vicinity of the constrained Cys, usually referred as the backdoor cysteine residue. We believe that the formation of cyclic sulfenyl amide has a slower kinetic rate as compared to disulfide bonds formation in these cases. In this work we have also identified two PFAM families, Glutamine amidotransferase and Carbon-nitrogen hydrolase, that lack experimental evidence of cysteine oxidation but have a relevant Cys in the active site. [80,90] In this sense, it would be interesting to conduct experiments to analyze possible redox regulation of members of these families.

Besides the previous interesting findings we searched in our list for proteins that are exposed to stress conditions. One example of these cases is the protein tyrosine phosphatases ptpB from *Mycobacterium tuberculosis(Mt)*. This protein has been reported to be involved in bacterial resistance to oxidative stress conditions found inside the macrophage, by modulating the activity of several cytosolic proteins. The role of ptpB is not completely clear, although one study points to the blocking of ERK1/2 and p38 IL-6 production pathways and Akt activation in the host cell [91]. On the other hand, the ptpA phosphatase of *Mt*, has the same fold but the cysteine was reported to be in a beta-sheet conformation near the forbidden zone, which could be a bias towards a more likely psi-dihedral angle. PtpA has been shown to dephosphorylate VPS33B, a component of the phagosome-lysosome fusion machinery [92], and has also been reported to bind to a proton ATPase subunit preventing the acidification of the phagosome[93]. Both proteins are key elements of the mycobacteria nitrosative stress response, and thus both proteins must act in an oxidative environment where Cys oxidation would be favored. In this scenario, the presence of a key cysteine in the forbidden-psi conformation would protect ptpA/B from oxidative damage, through the formation of the cyclic sulfenyl amide. Interestingly, we found that ptpA could be regulated by cyclic sulfenyl amide formation although it has not been detected. On the other hand, ptpB has an extra domain called “lid domain” which acts as a gate to the active site of this enzyme, protecting it from oxidative stress. [94]

### The formation of sulfenic acid and the following cyclic sulfenyl amide reaction mechanism

Cysteines residues have a rich chemistry and are involved in a plethora of redox reactions. Initial oxidation to sulfenic acid has been shown to be dependent on the cysteine pK_a_ [96–97]. It has been previously shown experimentally that in PTP1B the reactive cysteine is predominantly deprotonated at physiological pH [86], something necessary for the phosphatase activity, but also makes the cysteine susceptible to fast oxidation. In agreement, our simulations show that the pK_a_ decreases because the cysteinate is stabilized by the structural motif present in PTP1B (Also in other proteins identified in our study). We found that several NH groups from the backbone are able to perform hydrogen bonds with the negative sulfur atom due to the constrained cysteine and the beta-loop-helix motif. However, we found that the forbidden psi angle is not sufficient to lower the pK_a_ as in the model peptide its value is similar to the one of free cysteine in water.

Proteins that have a reactive cysteine in their active site that has a low pK_a_ are susceptible to inactivation by radical species like H_2_O_2_. The first step in this oxidation is the formation of sulfenic acid. In this work we found that a constrained conformation helps, once the sulfenic acid is formed, to protect its irreversible oxidation by forming a cyclic sulfenyl amide. According to our results the reaction mechanism that converts the sulfenic acid to a cyclic sulfenyl amide occurs through a seemingly dissociative mechanism, with a relative small free energy barrier. There is also a key role of the solvent that needs to be treated explicitly. Our findings indicate that the role of the protein in catalyzing the reaction is not due to the presence of nearby residues but to promote a constrained conformation necessary for the reaction to occur, as similar activation barriers are obtained in the protein and in a model peptide in water.

Experimentally, the reaction yielding the cyclic sulfenyl amide has been shown to occur in PTP1B as well as in several small model compounds.[32,33,95] In the protein, it is clear that the mechanism goes directly from the sulfenic acid to the cyclic product, and although its rate has not been measured, it should be able to compete with further Cys oxidation (to sulfonic acid) and thus protect the Cys in a physiological context. The relative small barrier obtained in the present work is consistent with this idea and underscores the likelihood of the presented mechanism. Last but not least, our results allow us to propose that the reaction should be promoted in acidic media, and thus show a pH dependent rate. Moreover, in the biological context, the presence of oxidative stress is usually accompanied by acidic conditions, and thus protection of key Cys through the present mechanism could be promoted.

We have also identified two families of proteins that have the constrained cysteine in several of its members but lack the beta-loop-helix motif. According to our QM/MM results on a model peptide those proteins could form the cyclic sulfenyl amide if the cysteine is oxidized to sulfenic acid. However, in the Retroviral aspartyl protease family no oxidation of the constrained cysteine has been reported while in the Beta-lactamase2 family oxidation has been reported but only to disulphide bond. As we have previously proposed for the PTEN family, if a backdoor cysteine is present, disulphide formation seems to be preferred to sulfenyl amide. Despite the fact that we have identified 125 proteins that only have the constrained cysteine, there is no experimental evidence, mainly due to the lack of available structural information. Moreover, the formation of sulfenic acid, has not been reported in these proteins.

Overall we have identified a group of proteins (270) that have a constrained Cysteine, located in a “forbidden” region of the Ramachandran plot (psi angle: -150 to -90°), that according to our QM/MM results, enhances the formation of sulfenyl amide when the Cysteine is oxidized to sulfenic acid. We also describe a subset of proteins (145) that have a beta-loop-helix motif which allows them to lower the pK_a_ enhancing their catalytic activity and also their reactivity towards ROS. In this subset, the constrained cysteine seems to be necessary for protection of the Cys residue from further oxidation as the cyclic sulfenyl amide can be then be recovered.

## Supporting Information

S1 Fig Computational workflow used for the detection of the constrained conformation of cysteine. (TIFF)

S2 Fig Cysteine Sulfenic Acid classical parameters. (TIFF)

S3 Fig Structural details of PTP1B Cys215-SOH. (A) Structure in the vicinity of Cys 215. ND and NE are Nitrogen Delta and Nitrogen Epsilon respectively. Dashed lines represent putative hydrogen bonds. (B) Histidine H-delta to Cysteine CO distance from His in the HID (Blue) and HIP (Green) states for the last 10ns of MD. (C) Density functions plot for Cys 215-S and Ser 216 H_N_ distance taken from PTP1B MD simulations with His 214 in the HIE (Red), HID (Blue) and HIP (Green) tautomer. (D) Same as C but with Cys 215-Os and Ser 216 H_N_ distance (E) Density function for Cys 215 dihedral angle when His 214 is in the HIE (red), HID (blue) and HIP (green) tautomer Average values are 126, 175 and -165 degrees respectively. Atoms names next to them. Color code of atoms: Carbon (Cyan), Nitrogen (Blue), Oxygen (Red), Sulphur (Yellow) and Hydrogen (White).
(TIFF)

S4 Fig Histogram plot of HMM search results score for: (A) Hidden Markov Model using sequences aligned at fixed cysteine position. (B) Hidden Markov Model using Aligned Structural Motif sequences.
(TIFF)

S5 Fig HMM models for (A) the Cysteine residue fixed covering 20 residues to the N and C terminal sides. (B) Structural alignment of the helix-beta-loop-helix.
(TIFF)

S6 Fig Structural alignment of the helix-beta-loop-helix in seven representantive structures of the different relevant PFam families. (A) PF00102 (1P15), (B) PF00117 (2VPI), (C) PF00581 (3D1P), (D) PF00782 (1D5R), (E) PF00795 (2PLQ), (F) PF01174 (2YWJ) and (G) PF01965 (1PDW)
(TIFF)

S7 Fig PTP1B Cysteine 215 pK_a_ lowering conformation. (A) Cys 215 forming hydrogen bonds between Cys 215-S and Ser 216-N Ala 217-N, Gly 218-N.
(TIFF)

S8 Fig psi Density function for a Cys-SOH containing peptide from molecular dynamics simulations. (TIFF)

S9 Fig Peptide energy profile. (A) Reaction schematics for the formation of cyclic sulfenyl amide in vacuum. (B) Energy profile for the peptide in vaccum (C) Energy profile for the sulfenamide formation reaction using reaction depicted in Fig. 2. (D) Structure of the transition state (TS) for the reaction in C. Distances are represented next to bonds or dashed lines. Atoms names next to them. Color code of atoms: Carbon (Cyan), Nitrogen (Blue), Oxygen (Red), Sulphur (Yellow) and Hydrogen (White).
(TIFF)

S10 Fig Peptide cyclic sulfenyl amide formation free energy profile. (A) First reaction coordinate. (B) Second reaction coordinate.
(TIFF)

S11 Fig PTP1B cyclic sulfenyl amide formation free energy profile. PTP1B with Histidine 214 in HIP tautomer state and H_3_O^+^ as proton donor shown in blue, PTP1B with Histidine 214 in HID tautomer and H_3_O^+^ as proton donor shown in red and PTP1B with Histidine in HID tautomer and H_2_O as proton donor in green.
(TIFF)

S1 Table Protein Crystal structures with sulfenyl amide deposited in Protein Data Bank. (PDF)

S2 Table Protein Crystal structures with cysteine sulfenic acid. (PDF)

S3 Table Protein Crystal structures with the cysteine between -150 and -90 psi angle (PDF)

S4 Table Proteins in the PDB in forbidden conformation Cys with the beta-loop-helix motif. (PDF)

S5 Table Proteins with cysteine in “forbidden conformation” and without the beta-loop-helix motif. (PDF)

S6 Table HMM search results against Swiss-Prot database. Highlighted in bold are the seven families described in the main text.
(PDF)

S7 Table Structural and energetic parameters for the cyclic sulfenyl amide formation in water (PDF)
