# Supplementary figures and images for: Protein Topology Determines Cysteine Oxidation Fate: The Case of Sulfenyl Amide Formation among Protein Families
Source: PLoS Comput Biol. 2015 Mar 5;11(3):e1004051. doi: 10.1371/journal.pcbi.1004051 (PMC4351059; doi:10.1371/journal.pcbi.1004051)

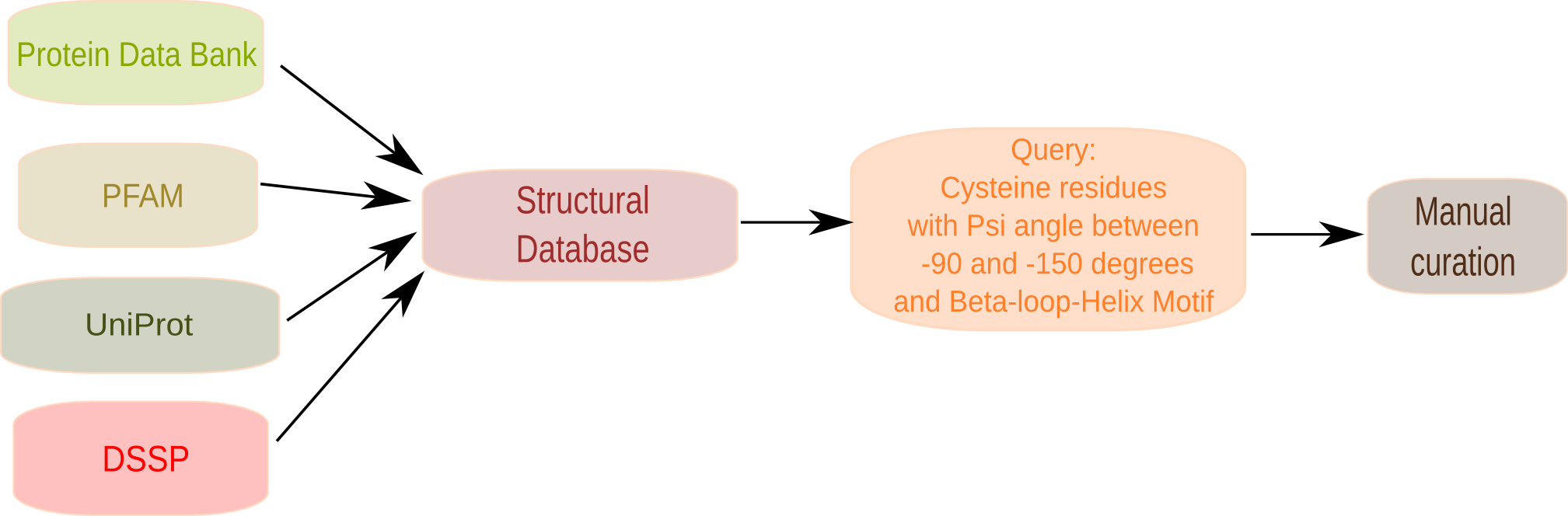

Supplement: S1 Fig — (TIFF) [file pcbi.1004051.s001.tiff]

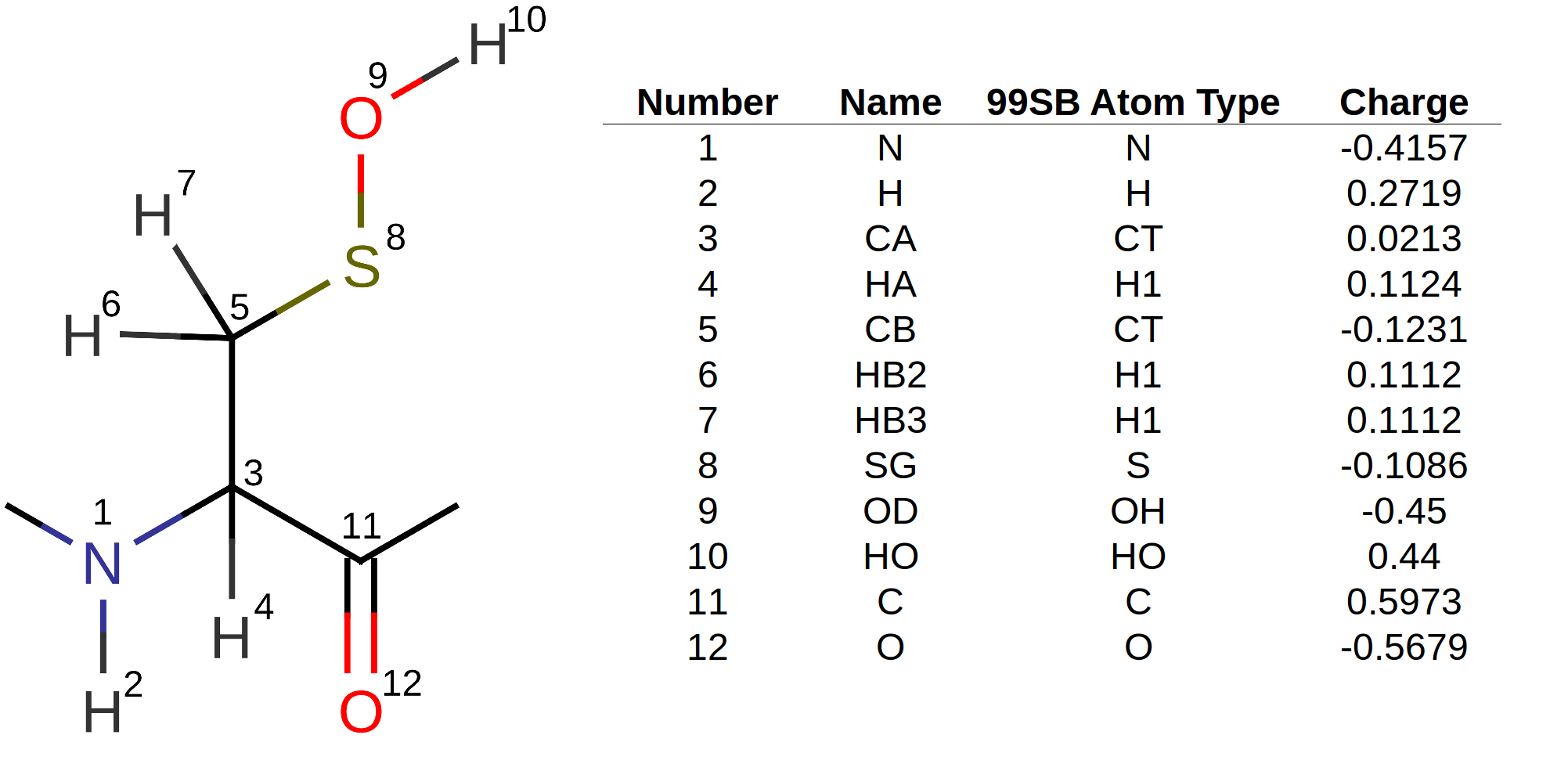

Supplement: S2 Fig — (TIFF) [file pcbi.1004051.s002.tiff]

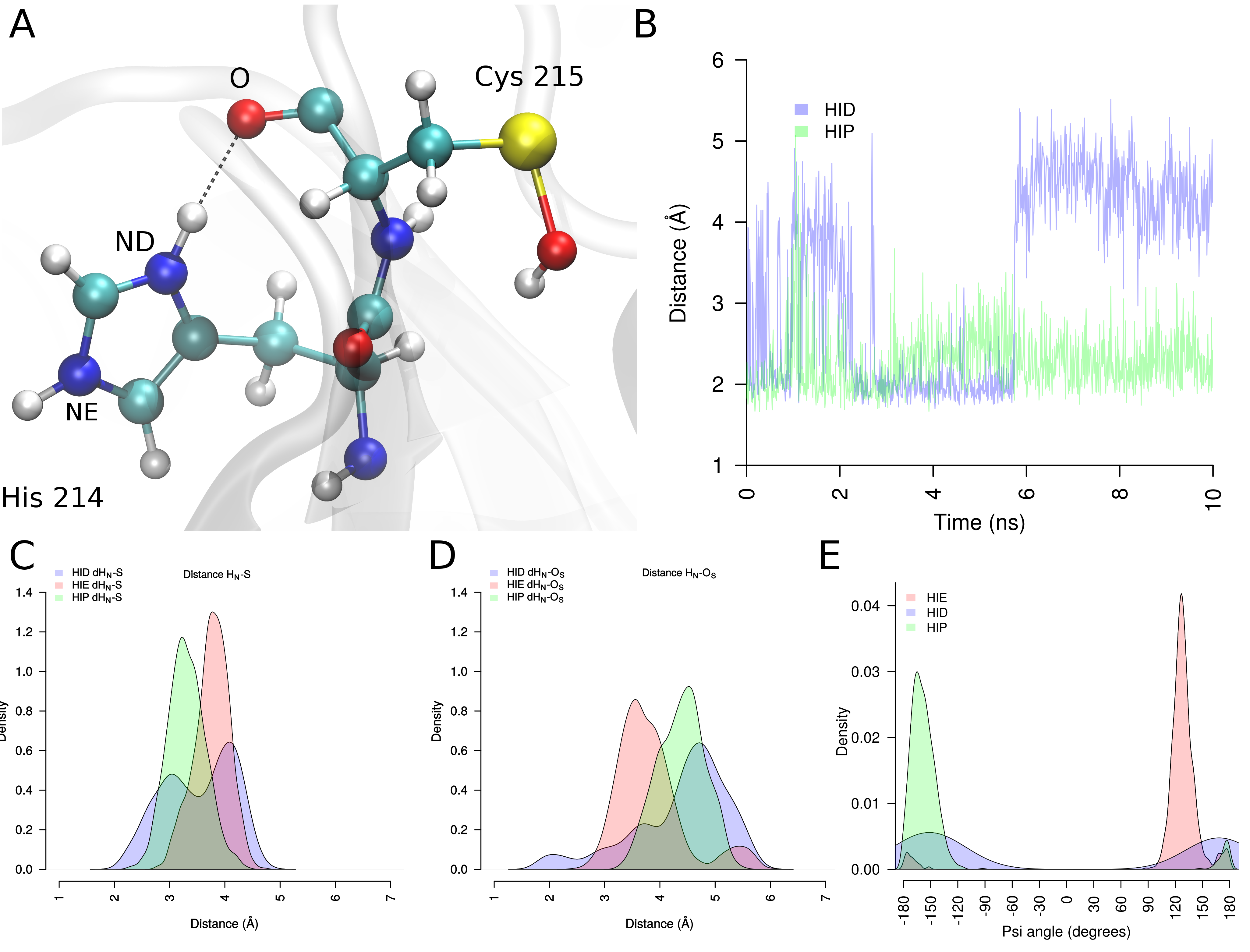

Supplement: S3 Fig — (A) Structure in the vicinity of Cys 215. ND and NE are Nitrogen Delta and Nitrogen Epsilon respectively. Dashed lines represent putative hydrogen bonds. (B) Histidine H-delta to Cysteine CO distance from His in the HID (Blue) and HIP (Green) states for the last 10ns of MD. (C) Density functions plot for Cys 215-S and Ser 216 HN distance taken from PTP1B MD simulations with His 214 in the HIE (Red), HID (Blue) and HIP (Green) tautomer. (D) Same as C but with Cys 215-Os and Ser 216 HN distance (E) Density function for Cys 215 dihedral angle when His 214 is in the HIE (red), HID (blue) and HIP (green) tautomer Average values are 126, 175 and -165 degrees respectively. Atoms names next to them. Color code of atoms: Carbon (Cyan), Nitrogen (Blue), Oxygen (Red), Sulphur (Yellow) and Hydrogen (White). (TIFF) [file pcbi.1004051.s003.tiff]

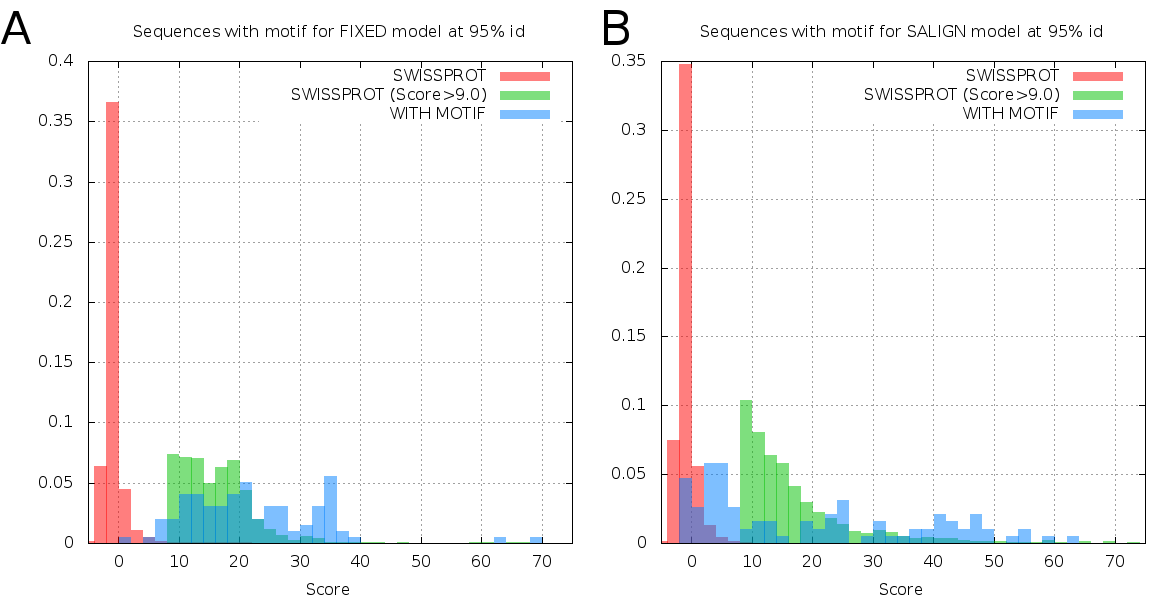

Supplement: S4 Fig — (A) Hidden Markov Model using sequences aligned at fixed cysteine position. (B) Hidden Markov Model using Aligned Structural Motif sequences. (TIFF) [file pcbi.1004051.s004.tiff]

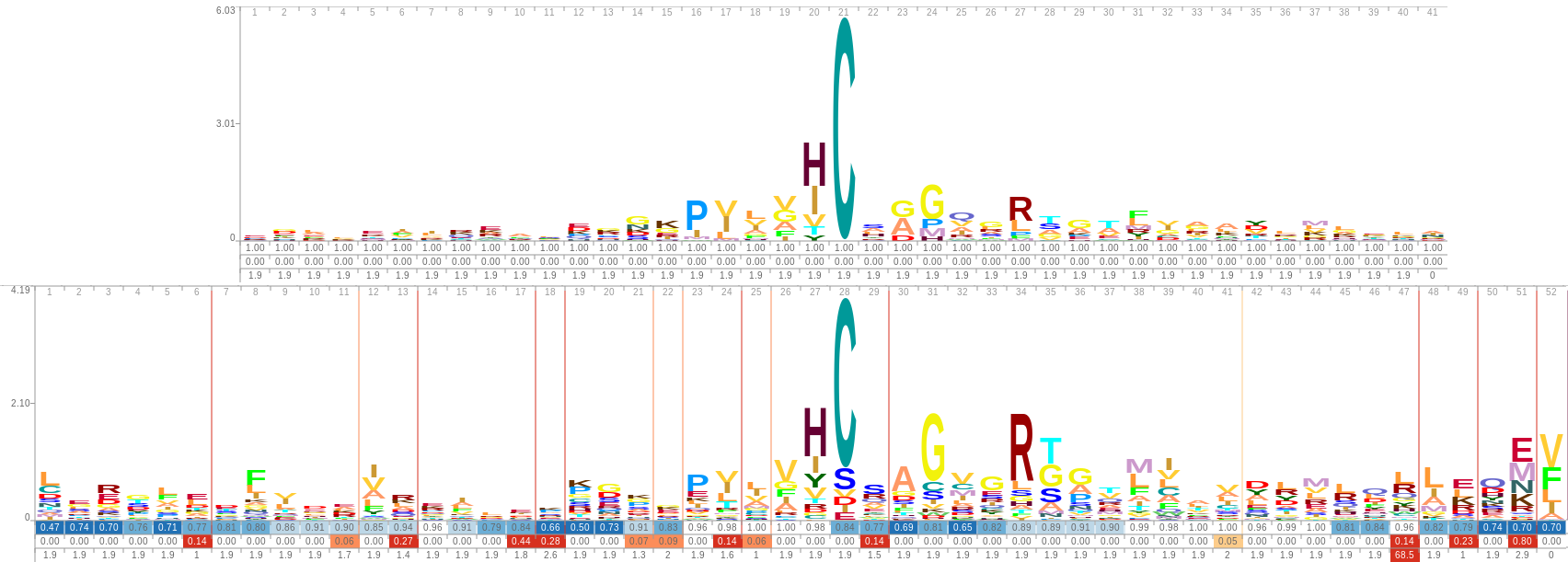

Supplement: S5 Fig — (B) Structural alignment of the helix-beta-loop-helix. (TIFF) [file pcbi.1004051.s005.tiff]

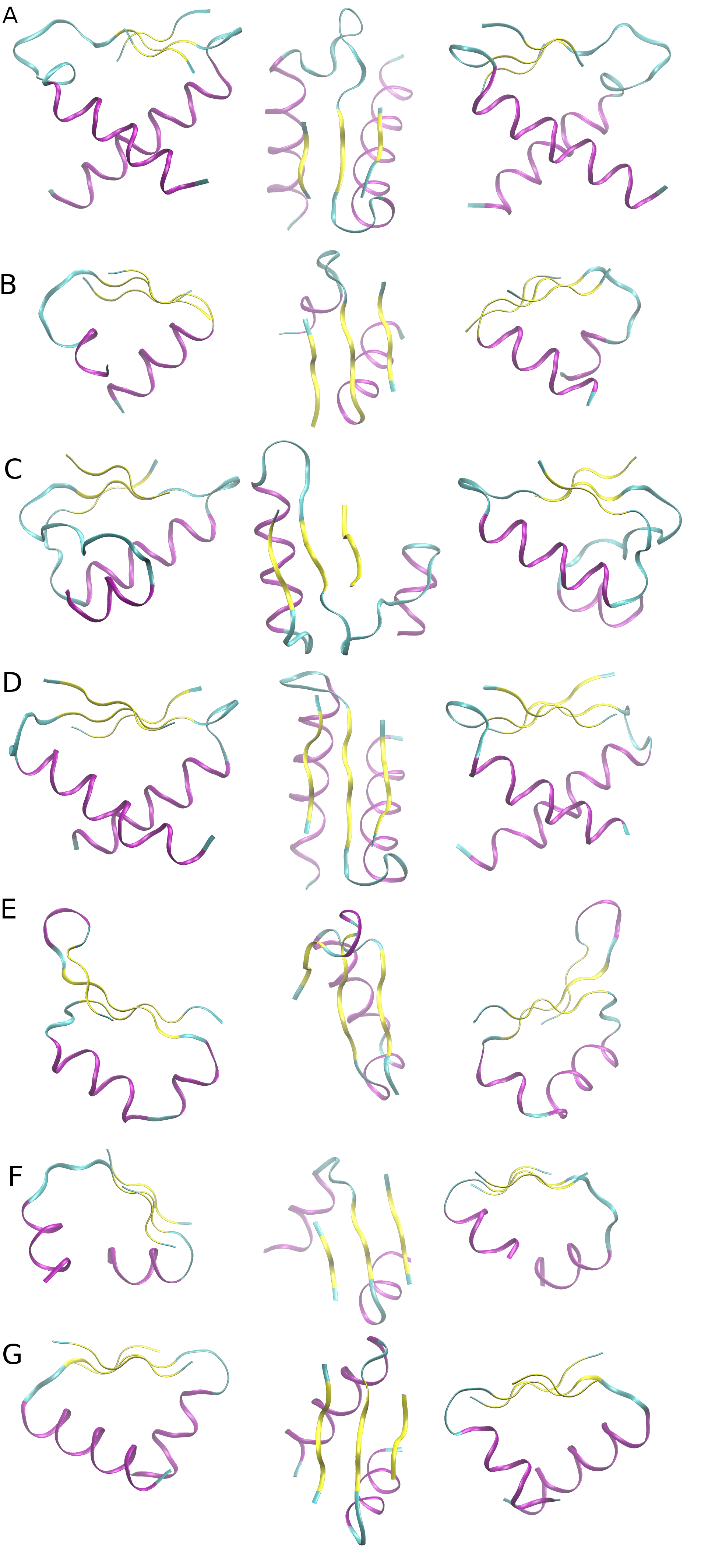

Supplement: S6 Fig — (A) PF00102 (1P15), (B) PF00117 (2VPI), (C) PF00581 (3D1P), (D) PF00782 (1D5R), (E) PF00795 (2PLQ), (F) PF01174 (2YWJ) and (G) PF01965 (1PDW) (TIFF) [file pcbi.1004051.s006.tiff]

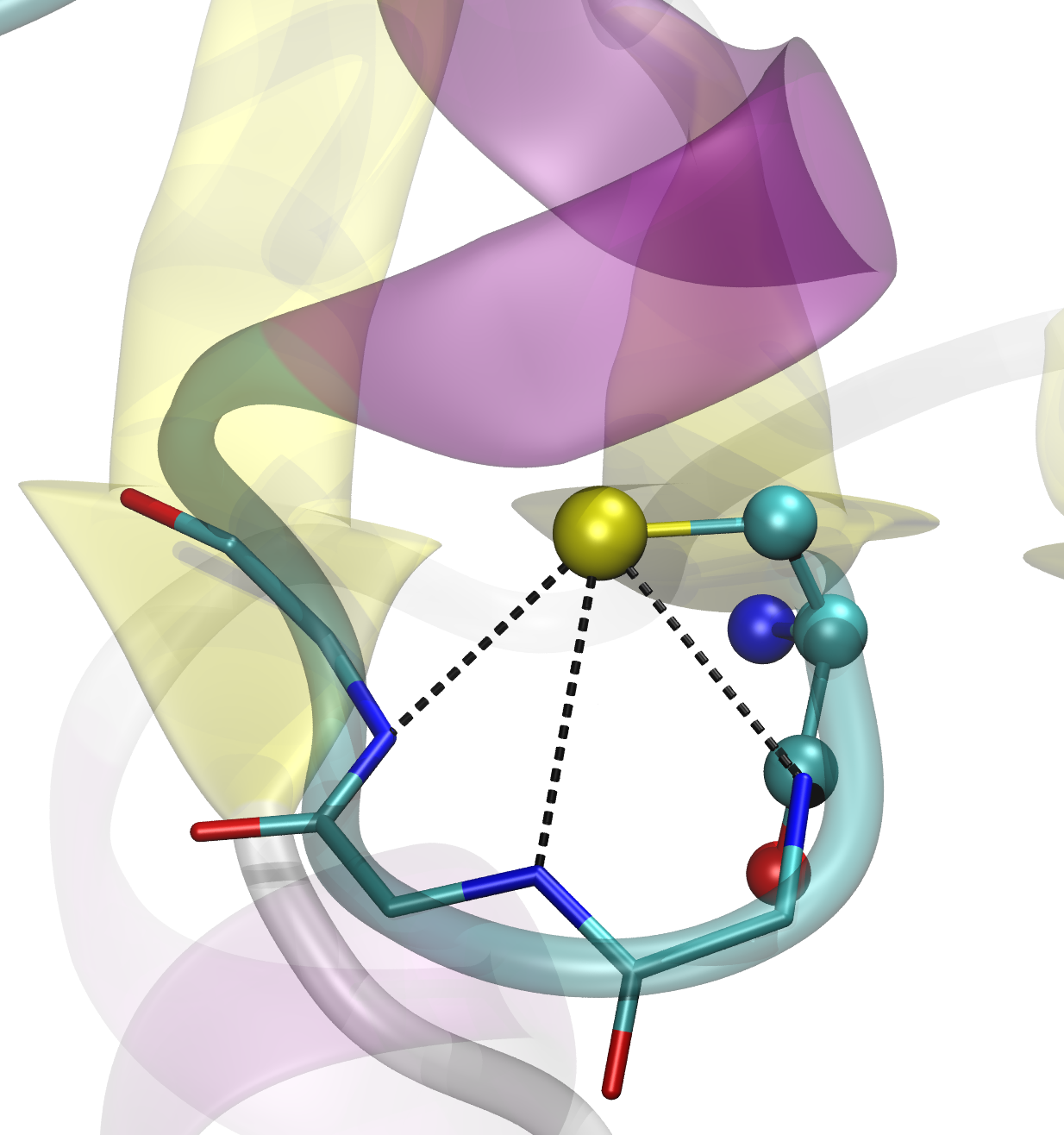

Supplement: S7 Fig — (A) Cys 215 forming hydrogen bonds between Cys 215-S and Ser 216-N Ala 217-N, Gly 218-N. (TIFF) [file pcbi.1004051.s007.tiff]

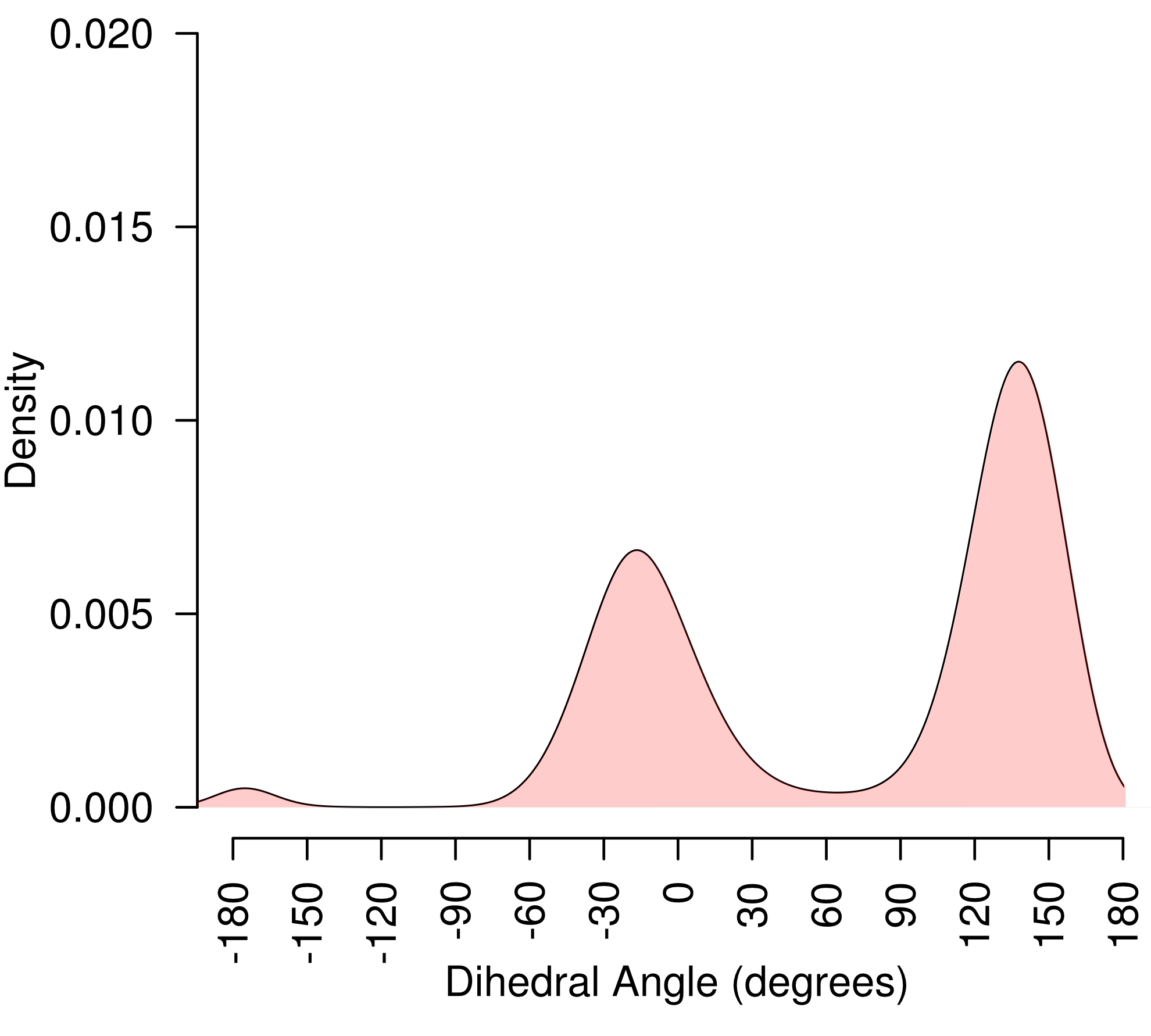

Supplement: S8 Fig — (TIFF) [file pcbi.1004051.s008.tiff]

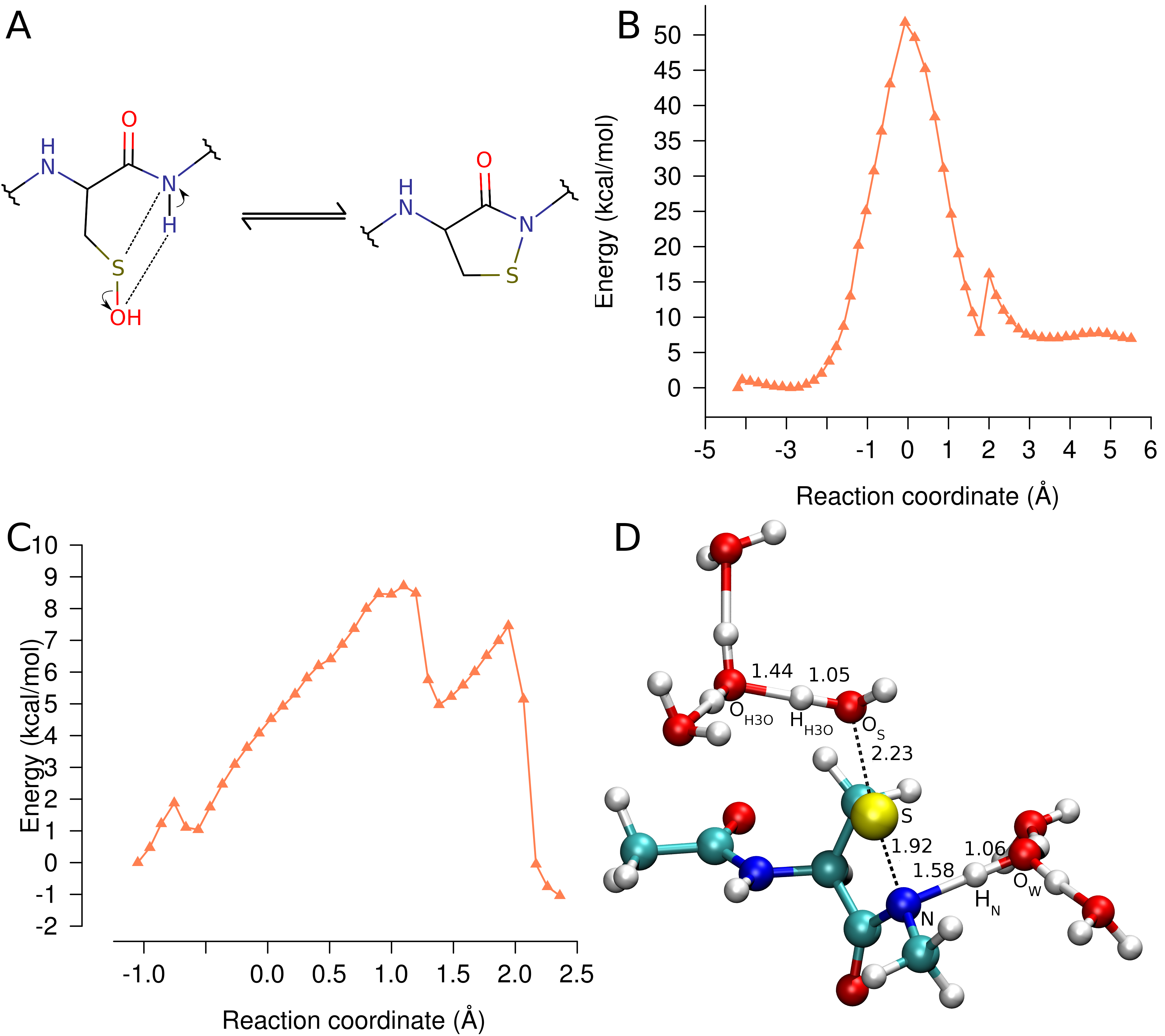

Supplement: S9 Fig — (A) Reaction schematics for the formation of cyclic sulfenyl amide in vacuum. (B) Energy profile for the peptide in vaccum (C) Energy profile for the sulfenamide formation reaction using reaction depicted in Fig. 2. (D) Structure of the transition state (TS) for the reaction in C. Distances are represented next to bonds or dashed lines. Atoms names next to them. Color code of atoms: Carbon (Cyan), Nitrogen (Blue), Oxygen (Red), Sulphur (Yellow) and Hydrogen (White). (TIFF) [file pcbi.1004051.s009.tiff]

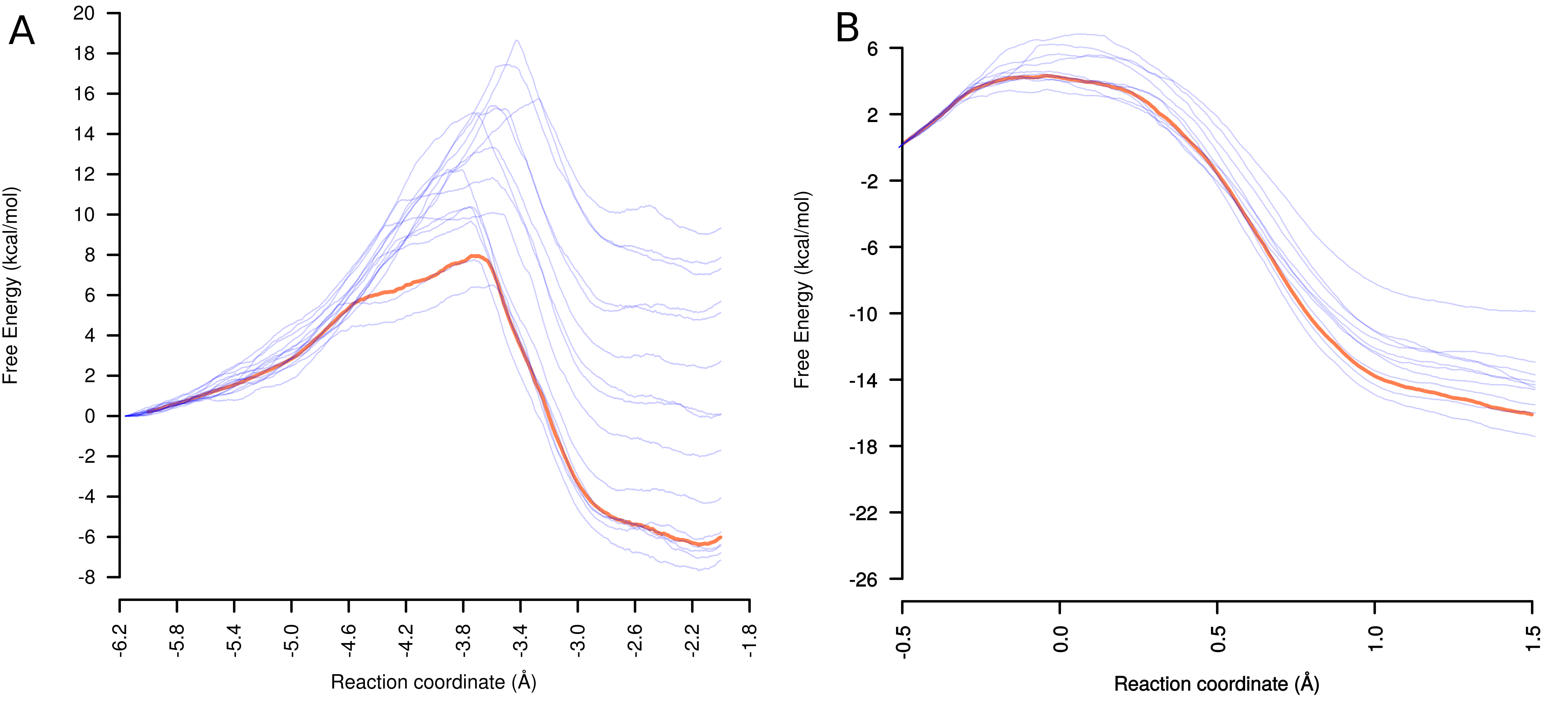

Supplement: S10 Fig — (A) First reaction coordinate. (B) Second reaction coordinate. (TIFF) [file pcbi.1004051.s010.tiff]

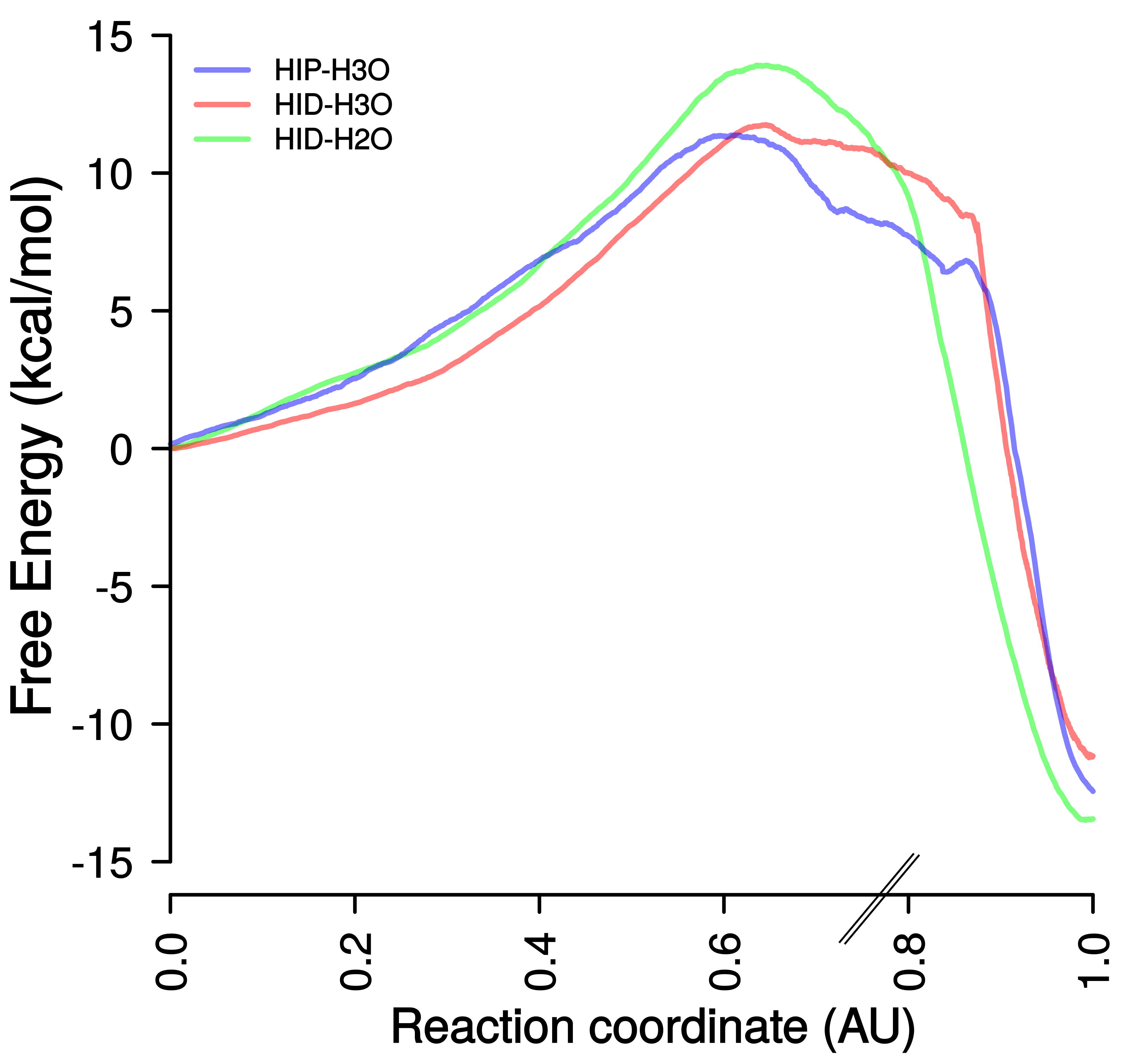

Supplement: S11 Fig — PTP1B with Histidine 214 in HIP tautomer state and H3O+ as proton donor shown in blue, PTP1B with Histidine 214 in HID tautomer and H3O+ as proton donor shown in red and PTP1B with Histidine in HID tautomer and H2O as proton donor in green. (TIFF) [file pcbi.1004051.s011.tiff]
