# Supplementary material for: Protein Topology Determines Cysteine Oxidation Fate: The Case of Sulfenyl Amide Formation among Protein Families
Source: PLoS Comput Biol. 2015 Mar 5;11(3):e1004051. doi: 10.1371/journal.pcbi.1004051 (PMC4351059; doi:10.1371/journal.pcbi.1004051)
Supplement: S1 Table — (PDF) [file pcbi.1004051.s012.pdf]

Table S1. Protein Crystal structures with sulfenyl amide deposited in Protein Data Bank.

UniProt	UniProt Name	PDBid	Chain	Residue
P18031	Protein-tyrosine phosphatase 1B	1oes	A	215
P18031	Protein-tyrosine phosphatase 1B	1oem	X	215
P18031	Protein-tyrosine phosphatase 1B	3sme	A	215
O53512	Phospho-2-dehydro-3-deoxyheptonate aldolase AroG	3nue	A	440
