# Supplementary material for: Protein Topology Determines Cysteine Oxidation Fate: The Case of Sulfenyl Amide Formation among Protein Families
Source: PLoS Comput Biol. 2015 Mar 5;11(3):e1004051. doi: 10.1371/journal.pcbi.1004051 (PMC4351059; doi:10.1371/journal.pcbi.1004051)
Supplement: S2 Table — (PDF) [file pcbi.1004051.s013.pdf]

Table S2. Protein Crystal structures with the cysteine between -150 and -90 psi angle

PFAM Family	UniProt	PDB	Chain	ResID	Phi	Psi
PF00102	M0R726	2nv5	C	1424	-132,5	-121,7
PF00102	M0R726	2nv5	B	1424	-131,4	-129
PF00102	M0R726	2nv5	A	1424	-127,8	-125
PF00102	O68720	1qz0	A	403	-124,5	-118,4
PF00102	O68720	1qz0	B	403	-123,9	-119,1
PF00102	A3QMF6	3f41	B	250	-128,2	-124,5
PF00102	A3QMF6	3f41	A	250	-136,8	-140,2
PF00102	O14522	2ooq	A	1106	-129,4	-122,9
PF00102	O14522	2ooq	B	1106	-130,7	-125
PF00102	P10586	1lar	A	1813	-140	-124,2
PF00102	P10586	1lar	A	1522	-134,6	-113,4
PF00102	P10586	1lar	B	1522	-141,7	-115,1
PF00102	P10586	1lar	B	1813	-134,6	-120,1
PF00102	P15273	1xxv	A	403	-117,7	-114,1
PF00102	P15273	1ypt	A	403	-110,3	-123
PF00102	P15273	1ypt	B	403	-112,3	-120,4
PF00102	P15273	3f9b	A	403	-111,9	-124,4
PF00102	P15273	1xxv	B	403	-119,1	-114,9
PF00102	P15273	2i42	A	403	-109,1	-120,9
PF00102	P15273	1ytn	A	403	-124,1	-118,7
PF00102	P15273	3blt	A	403	-176,5	-113,3
PF00102	P15273	3f9a	A	403	-109,6	-121,1
PF00102	P15273	3blu	A	403	-156,1	-129,9
PF00102	P15273	3f99	A	403	-106,9	-123,7
PF00102	P15273	1ytw	A	403	-99,9	-113,7
PF00102	P18031	2zmm	A	215	-122,1	-129,8
PF00102	P18031	2f6y	A	215	-128,1	-130,6
PF00102	P18031	3cwe	A	715	-136,6	-129
PF00102	P18031	2qbq	A	215	-106	-118,6
PF00102	P18031	1ecv	A	215	-125	-142,6
PF00102	P18031	1pxh	A	215	-140	-131
PF00102	P18031	1c83	A	215	-119,7	-144,8
PF00102	P18031	1q6s	A	715	-138,1	-130,3
PF00102	P18031	3eb1	A	215	-137,7	-124,3
PF00102	P18031	1bzj	A	215	-141,2	-138,8
PF00102	P18031	1l8g	A	215	-120,7	-132,8
PF00102	P18031	1lqf	C	715	-139,6	-124
PF00102	P18031	2f6t	A	215	-132,4	-132,6
PF00102	P18031	1sug	A	215	-134,6	-135,5
PF00102	P18031	2qbr	A	215	-125	-128,8
PF00102	P18031	1q6n	A	715	-137,2	-125,2
PF00102	P18031	1q6t	B	1215	-135,2	-137,8
PF00102	P18031	1q6p	A	715	-139	-131,2
PF00102	P18031	2fjm	A	715	-130,1	-133
PF00102	P18031	2f6w	A	215	-121,8	-130
PF00102	P18031	2fjn	A	715	-140,8	-128,7
PF00102	P18031	1t48	A	215	-137	-136,1
PF00102	P18031	1q6t	A	715	-134,8	-129,2
PF00102	P18031	1bzc	A	215	-140	-138,5
PF00102	P18031	2azr	A	215	-104,5	-125,5
PF00102	P18031	2qbs	A	215	-124,3	-124,2

PF00102	P18031	1q6s	B	1215	-136,9	-131,7
PF00102	P18031	3i80	A	215	-125,5	-129,3
PF00102	P18031	1bzh	A	215	-137,8	-129,3
PF00102	P18031	2f6f	A	215	-138,6	-141,1
PF00102	P18031	1c88	A	215	-129,7	-143,2
PF00102	P18031	2f6v	A	215	-131,5	-134
PF00102	P18031	1lqf	D	715	-142,4	-127,9
PF00102	P18031	1c87	A	215	-125,4	-139,2
PF00102	P18031	2hb1	A	215	-133,7	-133,4
PF00102	P18031	1g7g	A	215	-140,7	-141,4
PF00102	P18031	3eax	A	215	-136,6	-125,9
PF00102	P18031	1jf7	A	215	-141,1	-132,1
PF00102	P18031	3i7z	A	215	-132,5	-137,4
PF00102	P18031	2h4k	A	215	-126,3	-128,6
PF00102	P18031	2f6z	A	215	-133,4	-133,4
PF00102	P18031	2f71	A	215	-128,4	-135,3
PF00102	P18031	2nta	A	215	-132,4	-129,6
PF00102	P18031	2fjm	B	715	-127,6	-131,2
PF00102	P18031	1jf7	B	215	-137,8	-137,6
PF00102	P18031	1lqf	A	715	-138,3	-123,2
PF00102	P18031	1gfy	A	215	-123,8	-136,7
PF00102	P18031	1kav	A	215	-129,4	-126,2
PF00102	P18031	2zn7	A	215	-128,1	-131,3
PF00102	P18031	1g7f	A	215	-137,8	-128,7
PF00102	P18031	1lqf	B	715	-139,7	-129,7
PF00102	P18031	3a5k	A	215	-139,5	-133,6
PF00102	P18031	3sme	A	215	-139,6	-140,9
PF00102	P18031	2b07	A	215	-100,7	-122,8
PF00102	P18031	1kak	A	215	-130,9	-118,6
PF00102	P18031	1q6p	B	1215	-133,1	-134,7
PF00102	P18031	2fjn	B	715	-137,9	-130,5
PF00102	P18031	1t49	A	215	-132,8	-138,6
PF00102	P18031	1c86	A	215	-128,2	-136,4
PF00102	P18031	2f70	A	215	-128,5	-134,2
PF00102	P18031	2nt7	A	215	-133,9	-128,1
PF00102	P18031	1q6n	B	1215	-139,1	-131,3
PF00102	P18031	3a5j	A	215	-136,7	-134,7
PF00102	P18031	2h4g	A	215	-95	-123,6
PF00102	P18031	1nwl	A	215	-125,4	-133,5
PF00102	P18031	2qbp	A	215	-107,3	-110,9
PF00102	P18052	1yfo	B	433	-132,1	-128,7
PF00102	P18052	1p15	B	724	-134,4	-97,3
PF00102	P18052	1yfo	A	433	-136,3	-138,6
PF00102	P23467	2i4g	A	1904	-126	-125,3
PF00102	P23467	2i4h	A	1904	-128,2	-121,3
PF00102	P23467	2hc1	A	1904	-113,1	-137
PF00102	P23467	2ahs	B	1904	-131,1	-127,9
PF00102	P23467	2i5x	A	1904	-130,7	-127,1
PF00102	P23467	2i5x	B	1904	-130,2	-125,8
PF00102	P23467	2i4e	A	1904	-126,5	-130,9
PF00102	P23467	2i4e	B	1904	-129,3	-130,9
PF00102	P23467	2i3u	A	1904	-109,3	-146,4
PF00102	P23467	2i3r	A	1904	-128,5	-127,8
PF00102	P23467	2i3r	B	1904	-122	-124,9

PF00102	P23467	2hc2	A	1904	-121,2	-136,4
PF00102	P23467	2ahs	A	1904	-131,1	-123,3
PF00102	P23470	3qci	A	1060	-120,9	-116,7
PF00102	P23470	3qcf	A	1060	-132,4	-111,4
PF00102	P23470	3qcj	A	1060	-120,2	-117,6
PF00102	P23470	3qcb	A	1060	-124,4	-119,1
PF00102	P23470	3qcl	A	1060	-132,9	-122,4
PF00102	P23470	3qce	A	1060	-125,8	-120,7
PF00102	P23470	2h4v	A	1060	-130,6	-122,2
PF00102	P23470	3qcb	B	1060	-125,6	-118,3
PF00102	P23470	3qcm	A	1060	-132,7	-122
PF00102	P23470	3qce	B	1060	-122,5	-120,7
PF00102	P23470	3qck	A	1060	-125,1	-121,6
PF00102	P23470	2pbn	A	1060	-131,7	-122
PF00102	P23470	2h4v	B	1060	-131,1	-125,9
PF00102	P23470	3qch	A	1060	-121,7	-120,9
PF00102	P23470	3qcm	B	1060	-132,4	-121,8
PF00102	P23470	3qcf	B	1060	-116,6	-119,9
PF00102	P23470	3qcc	B	1060	-116,3	-124,7
PF00102	P23470	3qcg	A	1060	-123,4	-124,7
PF00102	P23470	3qcn	A	1060	-134,9	-126,8
PF00102	P23470	3qcd	A	1060	-125,7	-122
PF00102	P23470	3qcc	A	1060	-122,2	-124
PF00102	P26045	2b49	A	842	-129,4	-137,3
PF00102	P28827	1rpm	B	1095	-125,3	-124,9
PF00102	P28827	1rpm	A	1095	-125,6	-124,7
PF00102	P29074	2i75	A	852	-122,1	-121,2
PF00102	P35992	3s3e	B	242	-119,6	-125,9
PF00102	P35992	3s3e	A	242	-129	-115,9
PF00102	P74873	1g4w	R	481	-118,8	-129,8
PF00102	Q06124	2shp	A	459	-133,2	-127,5
PF00102	Q06124	2shp	B	459	-132,7	-130,9
PF00102	Q12923	1wch	A	2408	-143,2	-122,6
PF00102	Q13332	2fh7	A	1880	-123	-117
PF00102	Q13332	2fh7	A	1589	-129	-115,2
PF00102	Q15256	2a8b	A	588	-126,5	-110,6
PF00102	Q15262	2c7s	A	1083	-123,1	-119
PF00102	Q15678	2bzl	A	1121	-133,7	-124,1
PF00102	Q16827	2gjt	A	1136	-127,7	-115,6
PF00102	Q16827	2g59	A	225	-132	-121,7
PF00102	Q16827	2gjt	B	1136	-123,7	-122,9
PF00102	Q16849	2i1y	A	909	-129,7	-114,9
PF00102	Q16849	2i1y	B	909	-128,8	-115,9
PF00102	Q3MIV7	2h04	A	1904	-133,1	-125,9
PF00102	Q3MIV7	2h03	A	1904	-131,5	-125,6
PF00102	Q3MIV7	2h02	A	1904	-132	-127,4
PF00102	Q3MIV7	2h02	B	1904	-134,2	-125,8
PF00102	Q59EE0	2nlk	A	1060	-135,4	-124
PF00102	Q62132	1jln	A	480	-135,5	-122,9
PF00102	Q62884	3i36	A	1118	-127,5	-120,5
PF00102	Q7ARH8	2y2f	A	403	-115,9	-122
PF00102	Q7ARH8	2ydu	A	403	-118,2	-119,1
PF00102	Q7WUJ1	3d1o	B	252	-130,8	-138,4
PF00102	Q7WUJ1	1u26	B	241	-141,8	-141,2

PF00102	Q7WUJ1	2psz	A	252	-131	-144,3
PF00102	Q7WUJ1	3d1q	A	252	-129,2	-136,6
PF00102	Q7WUJ1	2psz	B	252	-131	-143
PF00102	Q7WUJ1	2b4p	A	252	-129,8	-144,1
PF00102	Q7WUJ1	2b4p	B	252	-128	-139,6
PF00102	Q7WUJ1	3d1h	B	252	-126,7	-136,8
PF00102	Q7WUJ1	1u26	A	241	-121,9	-142,4
PF00102	Q7WUJ1	3d1q	B	252	-129	-138,1
PF00102	Q7WUJ1	3d1o	A	252	-127,9	-142,3
PF00102	Q7WUJ1	3d1h	A	252	-134,4	-146
PF00102	Q92932	2qep	B	945	-142,9	-102,9
PF00102	Q92932	2qep	A	945	-140,2	-101,6
PF00102	Q97VZ7	2i6j	A	96	-129,9	-132,7
PF00102	Q97VZ7	2i6i	A	96	-125,2	-138,8
PF00102	Q97VZ7	3ro1	A	96	-132,7	-138,1
PF00102	Q97VZ7	2i6m	A	96	-129,4	-136,8
PF00102	Q99952	2oc3	A	229	-129,7	-139,2
PF00102	Q9Y2R2	2p6x	B	227	-125,2	-138
PF00102	Q9Y2R2	2p6x	A	227	-128	-132,3
PF00117	O59071	1wl8	A	79	57,9	-102,4
PF00117	O59071	2d7j	A	79	55,4	-103,1
PF00117	P00900	1i7q	B	85	57,3	-94,5
PF00117	P00900	1i7s	B	85	58	-112,3
PF00117	P00900	1i7q	D	85	58,5	-95,3
PF00117	P00900	1i7s	D	85	50,7	-102,7
PF00117	P00905	1i1q	B	83	59,9	-115
PF00117	P00907	1jdb	F	269	58,3	-107,7
PF00117	P00907	1jdb	C	269	57,6	-95,6
PF00117	P00907	1jdb	I	269	53,4	-109,2
PF00117	P00907	1m6v	B	269	60,8	-112,4
PF00117	P00907	1m6v	H	269	59,5	-108,8
PF00117	P00907	1t36	H	269	56,2	-110,3
PF00117	P00907	1m6v	D	269	52,6	-108,3
PF00117	P00907	1m6v	F	269	56,8	-119,4
PF00117	P00907	1t36	B	269	63,9	-106,7
PF00117	P00907	1t36	F	269	57,9	-113,9
PF00117	P00907	1jdb	L	269	68,5	-110
PF00117	P00907	1t36	D	269	65,3	-110,6
PF00117	P04079	1gpm	C	86	56,8	-111,8
PF00117	P04079	1gpm	D	86	51,9	-112,9
PF00117	P04079	1gpm	B	86	56,8	-110,7
PF00117	P04079	1gpm	A	86	51,8	-104,6
PF00117	P0A6F1	1bxr	H	269	55	-97,4
PF00117	P0A6F1	1bxr	B	269	67,3	-99,9
PF00117	P0A6F1	1ce8	F	269	58,6	-103,8
PF00117	P0A6F1	1bxr	D	269	56,6	-99,7
PF00117	P0A6F1	1ce8	H	269	51,5	-93,5
PF00117	P0A6F1	1bxr	F	269	62,1	-113,4
PF00117	P0A6F1	1ce8	D	269	60,3	-107,3
PF00117	P0A6F1	1ce8	B	269	62,4	-103,8
PF00117	P0A7E5	1s1m	A	379	52,1	-114,1
PF00117	P0A7E5	1s1m	B	379	56,5	-111,4
PF00117	P33734	1ox6	B	83	58,2	-111,8
PF00117	P33734	1ox4	A	83	59,6	-99,2

PF00117	P33734	1ox6	A	83	55,4	-110,4
PF00117	P49915	2vxo	B	104	55,2	-98,7
PF00117	P49915	2vpi	B	104	54,6	-111,5
PF00117	P49915	2vxo	A	104	73,3	-105,5
PF00117	P49915	2vpi	A	104	56,2	-108,7
PF00117	Q06129	1qdl	B	84	60,5	-110,7
PF00117	Q1H479	3m3p	A	89	58,2	-114,4
PF00117	Q5SI28	2ywc	C	78	50,5	-106,3
PF00117	Q5SI28	2ywc	A	78	54,9	-100,1
PF00117	Q5SI28	2ywc	D	78	50	-101,6
PF00117	Q5SI28	2ywb	C	78	56,2	-112,8
PF00117	Q5SI28	2ywc	B	78	53,5	-113
PF00117	Q5SI28	2ywb	B	78	47,9	-108,8
PF00117	Q5SI28	2ywb	D	78	58,4	-108
PF00117	Q5SI28	2ywb	A	78	55,3	-108,9
PF00117	Q5SIA8	1vcm	A	391	52,4	-106,2
PF00117	Q5SIA8	1vco	A	391	51,2	-98,2
PF00117	Q5SIA8	1vcn	A	391	53	-98,1
PF00117	Q7SIC0	1ka9	H	82	55,8	-100,4
PF00117	Q980S6	3nva	B	386	59,3	-111,3
PF00117	Q980S6	3nva	A	386	66,8	-117,5
PF00117	Q9HJM3	2a9v	B	80	59,2	-109,3
PF00117	Q9HJM3	2a9v	C	80	55,4	-110,6
PF00117	Q9HJM3	2a9v	A	80	53,7	-107,8
PF00117	Q9HJM3	2a9v	D	80	57,3	-110,2
PF00117	Q9NRF8	2vkt	A	399	65,9	-110,9
PF00117	Q9X0C8	1gpw	B	84	67	-132,9
PF00117	Q9X0C8	1k9v	F	384	59,1	-120,8
PF00117	Q9X0C8	1gpw	F	84	46,2	-117,1
PF00117	Q9X0C8	1gpw	D	84	53,4	-103,4
PF00581	E2QRA0	3p3a	B	246	-144,3	-126,5
PF00581	E2QRA0	3p3a	A	246	-144,2	-133,6
PF00581	O05793	3hwi	A	233	-131,3	-137,6
PF00581	O05793	3hwi	B	233	-135,6	-138,6
PF00581	O05793	3aax	A	233	-143,5	-143,9
PF00581	O05793	3aax	B	233	-160	-142,6
PF00581	P00586	1orb	A	247	-161	-128,6
PF00581	P25325	3olh	A	248	-165,2	-148,8
PF00581	P30304	1c25	A	430	-134,4	-128,6
PF00581	P30305	2ifd	A	473	-135,9	-140,3
PF00581	P42937	3f4a	A	90	-130,2	-137,4
PF00581	P42937	3f4a	B	90	-129,8	-141,5
PF00581	P52197	1h4m	X	230	-139,7	-125,3
PF00581	P52197	1h4k	X	230	-139,7	-139,1
PF00581	P78067	3ipp	B	385	-143,8	-130,2
PF00581	P78067	3ipp	A	385	-156,2	-127,9
PF00581	P96888	3hzu	A	245	-143,1	-119,4
PF00581	Q12305	3d1p	A	98	-134,3	-124,1
PF00581	Q5SHV8	2eg4	A	191	-157,2	-131,1
PF00581	Q6Q1Q5	2j6p	F	75	-125,4	-138,4
PF00581	Q6Q1Q5	2j6p	E	75	-126,5	-132,3
PF00581	Q6Q1Q5	2j6p	D	75	-134,4	-133,5
PF00581	Q6Q1Q5	2j6p	C	75	-137,2	-137,4
PF00581	Q6Q1Q5	2j6p	B	75	-137,5	-136,1

PF00581	Q6Q1Q5	2j6p	A	75	-135,4	-134,4
PF00581	Q81UT5	3icr	A	514	-119,5	-147,3
PF00581	Q81UT5	3ics	A	514	-124,1	-144,9
PF01965	A9CJS5	2fex	B	101	67,8	-137,1
PF01965	A9CJS5	2fex	A	101	62,4	-131,2
PF01965	A9CJS5	2fex	C	101	67,4	-135,6
PF01965	B6YXG0	3l18	A	100	63,7	-120,6
PF01965	B6YXG0	3l18	B	100	61,7	-116,7
PF01965	O59413	1g2i	A	100	59,7	-115,1
PF01965	O59413	1g2i	C	500	61,3	-118,5
PF01965	O59413	1g2i	B	300	60,9	-119,3
PF01965	P31658	1n57	A	185	65,5	-116
PF01965	P31658	1ons	A	184	56,9	-115,3
PF01965	P96658	3f5d	A	100	54,8	-127,8
PF01965	Q04432	1rw7	A	138	70,4	-121,9
PF01965	Q08914	3kkl	A	138	75,3	-116
PF01965	Q08914	3kkl	B	138	72,7	-123,4
PF01965	Q2GI86	3l3b	A	137	64,5	-113,8
PF01965	Q2GI86	3l3b	B	137	67,7	-113,6
PF01965	Q46948	2ab0	A	106	55,7	-112,2
PF01965	Q46948	2ab0	B	106	72,7	-115
PF01965	Q81PY3	3efe	B	110	65,3	-130,9
PF01965	Q81PY3	3efe	F	110	60,1	-131,2
PF01965	Q81PY3	3efe	C	110	64,4	-121
PF01965	Q81PY3	3efe	A	110	66,6	-133,8
PF01965	Q81PY3	3efe	E	110	60	-121,6
PF01965	Q81PY3	3efe	D	110	71,3	-127,2
PF01965	Q8A8A4	3cne	D	111	52,7	-106,4
PF01965	Q8A8A4	3cne	C	111	51,7	-104,1
PF01965	Q8A8A4	3cne	A	111	58,6	-111,9
PF01965	Q8A8A4	3cne	B	111	59,9	-111,8
PF01965	Q99497	1pdw	H	3306	68	-108,5
PF01965	Q99497	1pdw	C	1106	64,1	-104,8
PF01965	Q99497	2or3	A	106	72,5	-107,4
PF01965	Q99497	2r1u	A	106	64,9	-108,6
PF01965	Q99497	3cz9	A	106	78,5	-102,7
PF01965	Q99497	3cza	A	106	65,9	-104,7
PF01965	Q99497	1pdv	A	106	66,4	-114,2
PF01965	Q99497	3cy6	A	106	72,3	-110,5
PF01965	Q99497	3sf8	B	106	75,6	-105,6
PF01965	Q99497	3bwe	D	706	63,1	-114,5
PF01965	Q99497	1q2u	A	106	66,5	-113
PF01965	Q99497	1pe0	B	306	65,4	-107,2
PF01965	Q99497	2or3	B	106	72	-106,4
PF01965	Q99497	1pdw	F	2306	60,6	-111,6
PF01965	Q99497	1pdw	B	306	63,1	-108,4
PF01965	Q99497	1ucf	A	106	66,1	-110,9
PF01965	Q99497	3bwe	G	1306	62,2	-110,5
PF01965	Q99497	1pe0	A	106	66,3	-106,2
PF01965	Q99497	3bwe	A	106	70,5	-105,6
PF01965	Q99497	3b38	A	106	74,7	-107,1
PF01965	Q99497	1pdw	D	1306	60,1	-109,1
PF01965	Q99497	1pdw	G	3106	57,9	-106,8
PF01965	Q99497	2r1u	B	106	65,5	-107,8

PF01965	Q99497	1p5f	A	106	77	-101
PF01965	Q99497	3bwe	E	906	73,3	-107,6
PF01965	Q99497	3bwe	B	306	69,8	-106,5
PF01965	Q99497	1j42	A	106	64,5	-113,4
PF01965	Q99497	3b3a	A	106	73,2	-113,1
PF01965	Q99497	3b36	A	106	73,7	-112,1
PF01965	Q99497	1ucf	B	106	65	-107,9
PF01965	Q99497	1pdw	E	2106	69,3	-95,9
PF01965	Q99497	1pdw	A	106	65,5	-104,1
PF01965	Q99497	3bwe	C	506	65,1	-108,2
PF01965	Q9HEU1	3n7t	A	141	79,7	-100,6
PF01965	Q9KPQ8	3ot1	A	108	64,8	-125,8
PF00782	O95147	2wgp	A	111	-128,9	-139
PF00782	O95147	2wgp	B	111	-123,2	-140,3
PF00782	P24656	1yn9	C	119	-124,3	-148,1
PF00782	P24656	1yn9	B	119	-123,6	-146,4
PF00782	P33064	2p4d	A	110	-126,1	-142,7
PF00782	P51452	1vhr	B	124	-141,6	-143,6
PF00782	P51452	3f81	B	124	-138,5	-143,3
PF00782	P51452	1vhr	A	124	-146,7	-148,1
PF00782	P51452	3f81	A	124	-137,2	-146,8
PF00782	P60484	1d5r	A	124	-129,3	-126,3
PF00782	Q4QEZ7	3s4o	A	112	-131,5	-103,5
PF00782	Q66GT5	3rgo	A	102	-124,9	-103,6
PF00782	Q68J44	2y96	A	147	-134,4	-132,9
PF00782	Q68J44	2y96	B	147	-139,7	-149,2
PF00782	Q8NEJ0	2esb	A	104	-138,2	-143,3
PF00782	Q9BVJ7	2img	A	95	-137,6	-146,4
PF00782	Q9NRW4	1wrm	A	88	-131,4	-143,8
PF00782	Q9UII6	2pq5	C	138	-134,9	-133,8
PF00782	Q9UII6	2pq5	D	138	-131,8	-129,8
PF00782	Q9UII6	2pq5	B	138	-127,2	-141,6
PF00782	Q9UII6	2gwo	D	138	-123	-147,4
PF00782	Q9UII6	2pq5	A	138	-136,6	-129,7
PF00782	Q9Y6W6	1zzw	B	408	-139,6	-119,4
PF00782	Q9Y6W6	1zzw	A	408	-135,1	-136,3
PF00795	O25836	2dyv	B	166	53	-106,1
PF00795	O25836	2dyv	A	166	52,2	-107,2
PF00795	O25836	2dyu	B	166	50,6	-114,9
PF00795	O25836	2dyu	A	166	53	-116,8
PF00795	P49954	1f89	B	469	32,4	-97,5
PF00795	P49954	1f89	A	169	33,3	-98,3
PF00795	P60327	1erz	A	171	43,7	-117,3
PF00795	P60327	1erz	B	171	43,3	-116,2
PF00795	Q44185	1fo6	C	172	47,3	-117,8
PF00795	Q44185	2ggl	C	172	45,6	-117,3
PF00795	Q44185	1fo6	A	172	45,4	-116,6
PF00795	Q44185	2ggl	B	172	53,3	-110,2
PF00795	Q44185	2ggk	D	172	39	-105,6
PF00795	Q44185	1fo6	D	172	48,1	-119,8
PF00795	Q44185	2ggl	A	172	47,9	-113,3
PF00795	Q44185	2ggk	B	172	52,1	-109,2
PF00795	Q44185	2ggk	A	172	47,7	-112,4
PF00795	Q44185	1fo6	B	172	47,4	-106,6

PF00795	Q44185	2ggl	D	172	37	-106,7
PF00795	Q44185	2ggk	C	172	49,1	-116,1
PF00795	Q82AV7	3n05	B	161	36,2	-94,6
PF00795	Q82AV7	3n05	A	161	34,6	-103,7
PF00795	Q8P8M3	2,00E+011	D	143	41,8	-107,9
PF00795	Q8P8M3	2,00E+011	A	143	37,2	-102,6
PF00795	Q8P8M3	2,00E+011	C	143	41,7	-104,9
PF00795	Q8P8M3	2,00E+011	B	143	37,6	-107,1
PF00795	Q9L543	2plq	A	166	45,6	-109,1
PF01174	Q5ND68	2abw	A	87	53,2	-109,5
PF01174	Q5ND68	2abw	B	87	54,7	-110,4
PF01174	P37528	2nv2	P	79	66,3	-99,6
PF01174	P37528	2nv2	B	79	73,9	-100
PF01174	P37528	2nv2	V	79	68,4	-95,1
PF01174	P37528	2nv2	R	79	70	-102,8
PF01174	P37528	1r9g	B	79	50,1	-134,8
PF01174	P37528	2nv2	N	79	60,9	-94,7
PF01174	P37528	2nv2	J	79	67	-102
PF01174	P37528	2nv0	B	79	49,4	-130,5
PF01174	P37528	2nv2	X	79	66,5	-101
PF01174	P37528	2nv2	H	79	63,1	-101,1
PF01174	P37528	2nv2	L	79	66,5	-99,1
PF01174	P37528	2nv2	D	79	63,5	-103,6
PF01174	P37528	2nv0	A	79	53,2	-120,8
PF01174	P37528	1r9g	A	79	47,4	-124,4
PF01174	P37528	2nv2	F	79	58,4	-103,1
PF01174	P37528	2nv2	T	79	58,5	-99,8
PF01174	P83813	1q7r	A	78	54,2	-136
PF01174	Q59055	2ywj	A	75	56,5	-99,4
PF01174	Q5SKD6	2ywd	A	81	61,2	-108,7
PF13278	A3QGX4	3bhn	A	100	47,9	-124,8
PF13278	Q4K977	3nor	A	104	65,2	-134,3
PF13278	Q4K977	3nov	A	104	49,9	-131,6
PF13278	Q881P0	3ewn	A	154	59,8	-143
PF13278	Q97FB4	3mgk	A	102	60,1	-142,5
PF13278	Q97FB4	3mgk	B	102	56,7	-137,1
PF00300	P07953	1tip	A	142	-128,5	-137,3
PF00300	P07953	1tip	B	142	-131,4	-135,8
PF00300	P07953	1c80	A	142	-125,6	-140,5
PF00300	P07953	1fbt	B	141	-125,9	-135,8
PF00300	P07953	1c80	B	142	-124,2	-136,3
PF00300	P07953	1fbt	A	141	-132,8	-132,9
PF00300	P07953	1c81	A	391	-124,2	-132,6
PF00300	P16118	1k6m	B	391	-133	-128,5
PF00300	P16118	1k6m	A	391	-132,8	-127,6
PF00300	P25114	1bif	A	389	-132,9	-134,3
PF00300	P25114	2bif	B	389	-132,1	-133
PF00300	P25114	3bif	A	389	-132,3	-138,7
PF00300	P25114	2bif	A	389	-132,4	-130,2
PF00300	Q16875	2axn	A	386	-133,1	-134,8
PF00300	Q16875	3qpw	A	386	-140,5	-134,3
PF00300	Q16875	2dwo	A	386	-131,5	-138,4
PF00300	Q16875	3qpu	A	386	-138	-135,2
PF00300	Q16875	2i1v	B	386	-138,6	-131,9

PF13350	A1QMT0	2oz5	A	160	-130,8	-124,5
PF13350	A1QMT0	2oz5	B	160	-131,9	-119,2
PF13350	A3QMF6	3f41	A	548	-124,9	-134,8
PF13350	A3QMF6	3f41	B	548	-131,6	-134,9
PF13350	P96830	1ywf	A	160	-127,3	-126,5
PF00106	A4VVQ2	3cxr	A	149	-93,5	-139,6
PF00106	A4VVQ2	3o03	A	149	-92,5	-140,5
PF00106	P10807	1b16	B	137	-99,5	-114,8
PF00106	P10807	1b2l	A	137	-103,6	-111,6
PF00106	P10807	1a4u	B	137	-108,9	-118,1
PF00106	P10807	1b16	A	137	-99	-114,3
PF00106	P10807	1a4u	A	137	-109	-118,2
PF00106	P10807	1sby	A	137	-114,9	-115
PF00106	P10807	1b15	B	137	-105,2	-112,9
PF00106	P10807	1b15	A	137	-95,4	-113,3
PF00106	P10807	1sby	B	137	-101,1	-115,4
PF00106	P10807	1b14	A	137	-102	-108,8
PF00106	P10807	1b14	B	137	-101,6	-109,5
PF00106	Q48436	1geg	A	138	-110,1	-141,8
PF00106	Q48436	1geg	G	138	-109,4	-141,9
PF00106	Q48436	1geg	F	138	-109,9	-140,8
PF00106	Q48436	1geg	B	138	-109,4	-139,7
PF00106	Q48436	1geg	C	138	-110	-141,7
PF00106	Q48436	1geg	D	138	-112	-142,8
PF00106	Q48436	1geg	E	138	-110,7	-141,5
PF00106	Q48436	1geg	H	138	-109,5	-142,1
PF01909	P06766	2van	A	178	-105,8	-140,9
PF01909	P06766	1zqx	A	178	-81,9	-140,3
PF01909	P06766	1zqy	A	178	-94,2	-145,3
PF01909	P06766	1bpb	A	178	-88,1	-145,5
PF01909	P06766	1jn3	A	178	-103,5	-143,8
PF01909	Q5SJ64	3au2	A	186	-113,5	-138,9
PF01909	Q9UGP5	2pfn	A	415	-111	-141,3
PF01909	Q9UGP5	1xsp	A	415	-110,3	-136,7
PF01909	Q9UGP5	2gws	M	415	-106,4	-133,8
PF01909	Q9UGP5	3mda	A	415	-114,9	-137,4
PF01909	Q9UGP5	1xsl	I	415	-107,9	-134,4
PF01909	Q9UGP5	3pnc	A	415	-104,5	-134,2
PF01909	Q9UGP5	2gws	E	415	-97,6	-136,9
PF01909	Q9UGP5	3c5f	B	415	-106,3	-137,4
PF01909	Q9UGP5	2gws	I	415	-103,2	-136,7
PF01909	Q9UGP5	1rzt	E	415	-110,2	-136,4
PF01909	Q9UGP5	3c5f	A	415	-107,9	-136,6
PF01909	Q9UGP5	2pfo	A	415	-110,6	-132,5
PF01909	Q9UGP5	2gws	A	415	-103,7	-134,1
PF01909	Q9UGP5	3mgh	C	415	-108,6	-134,5
PF01909	Q9UGP5	3c5g	A	415	-109,1	-141,4
PF01909	Q9UGP5	1rzt	I	415	-106,1	-137,4
PF01909	Q9UGP5	1rzt	A	415	-106,6	-133,9
PF01909	Q9UGP5	1xsn	A	415	-109,7	-138
PF01909	Q9UGP5	2bcv	A	415	-117,1	-142,4
PF01909	Q9UGP5	2bcu	A	415	-114,2	-140,8
PF01909	Q9UGP5	3mgh	A	415	-108,9	-138,7
PF01909	Q9UGP5	2bcs	A	415	-115,6	-142

PF01909	Q9UGP5	1xsl	M	415	-108,7	-138,8
PF01909	Q9UGP5	2pfp	A	415	-118,4	-134
PF01909	Q9UGP5	3pmn	A	415	-102,3	-131,2
PF01909	Q9UGP5	1rzt	M	415	-107,2	-135,8
PF01909	Q9UGP5	1xsl	E	415	-104,2	-139
PF01909	Q9UGP5	2pfq	A	415	-104,7	-137,4
PF01909	Q9UGP5	2bcq	A	415	-110,6	-138,7
PF01909	Q9UGP5	3c5g	B	415	-112,5	-139,2
PF01909	Q9UGP5	3hwt	A	415	-111,3	-140,4
PF01909	Q9UGP5	2bcr	A	415	-114,6	-136,9
PF01909	Q9UGP5	3hw8	A	415	-101,1	-136,6
PF01909	Q9UGP5	3mdc	A	415	-113,6	-143,1
PF01909	Q9UGP5	1xsl	A	415	-106,4	-131,5
PF00384	P81186	2nap	A	307	-139,2	-135,9
PF00384	P81186	2jio	A	307	-141,2	-125,8
PF00384	P81186	2jip	A	307	-143,5	-125,1
PF00384	P81186	2jim	A	307	-148,7	-130,8
PF00384	P81186	2v45	A	307	-139,7	-127,3
PF00384	P81186	2v3v	A	307	-144,6	-121,4
PF00384	P81186	2jir	A	307	-138,8	-126,1
PF00384	P81186	2jiq	A	307	-137	-126,6
PF02574	Q93088	1lt8	A	299	-123,9	-144,2
PF02574	Q9WYA5	1q85	B	272	-114,3	-149,8
PF02574	Q9WYA5	1q7z	B	272	-108,5	-125,4
PF02574	Q9WYA5	1q85	A	272	-111,7	-140,1
PF02574	Q9WYA5	3bol	A	272	-111,8	-148,7
PF02574	Q9WYA5	1q7z	A	272	-111	-128,5
PF07722	Q92820	1l9x	B	110	53,3	-101
PF07722	Q92820	1l9x	D	110	54,5	-96,6
PF07722	Q92820	1l9x	C	110	54,5	-102,7
PF07722	Q92820	1l9x	A	110	51,9	-100
PF07722	Q92AL3	3fij	F	115	47,4	-108,7
PF07722	Q92AL3	3fij	E	115	49	-109,2
PF07722	Q92AL3	3fij	G	115	45,2	-110,3
PF07722	Q92AL3	3fij	C	115	57,6	-117,6
PF07722	Q92AL3	3fij	A	115	50	-115,6
PF07722	Q92AL3	3fij	D	115	58,4	-124
PF07722	Q92AL3	3fij	H	115	45,5	-109,9
PF07722	Q92AL3	3fij	B	115	55,3	-118,1
PF12695	Q01609	2hdw	A	179	52,7	-128,2
PF12695	Q01609	2hdw	B	179	48	-128
PF12850	Q9QZ88	1z2w	A	41	82,2	-140
PF00857	Q82NB5	3kl2	D	180	-122,9	-90
PF00857	Q82NB5	3kl2	B	180	-112,4	-100,3
PF00857	Q82NB5	3kl2	F	180	-122,3	-104,2
PF00857	Q82NB5	3kl2	L	180	-113,2	-92,1
PF00857	Q82NB5	3kl2	H	180	-116,7	-94,6
PF00857	Q82NB5	3kl2	A	180	-113,4	-101,9
PF00857	Q82NB5	3kl2	G	180	-115,6	-96,2
PF00857	Q82NB5	3kl2	E	180	-116,3	-95,2
PF00857	Q82NB5	3kl2	K	180	-125,5	-92,7
PF00142	P00459	1g20	E	151	-143,6	-149,5
PF02979	O66188	2dd4	L	131	-172,3	-122,6
PF02979	O66188	2dd4	I	131	-171	-121,2

PF02979	O66188	2dd4	F	131	-174,2	-123,4
PF02979	O66188	2dd4	C	131	-173,1	-120,1
PF00162	P00560	3pgk	A	97	-130,2	-99
PF01329	P61459	1dco	G	82	-95,8	-91,1
PF02550	Q9RM86	3gk7	B	32	57,7	-113,9
PF02550	Q9RM86	3gk7	A	32	52,4	-115,1
PF04204	Q72X44	2ghr	A	142	44,8	-116,9
PF04204	Q72X44	2vdj	A	142	47,7	-112,8
PF02826	P60802	3oet	G	207	-101,9	-90,4
PF03372	P00639	2a42	B	173	60,7	-129,7
PF03372	P00639	2dnj	A	173	49,9	-128,8
PF03372	P00639	1dnk	A	173	44,9	-125
PF01738	A6TGL0	3f67	A	120	65,3	-118,9
PF11486	O34626	2euc	A	29	-70,9	-120,8
PF11486	O34626	2euc	B	29	-70,4	-121,5
PF00680	P12916	1xr6	A	289	-161,3	-137,3
PF01008	Q5JFM9	3a11	B	133	55,8	-149,6
PF01008	Q5JFM9	3a11	D	133	56,3	-150
PF02774	P44801	1oza	A	136	42,8	-138,4
PF02776	P0CH62	2pgn	B	77	-8	-91,4
PF01083	P00590	1cuj	A	120	54,6	-123
PF04273	A4Y1H6	3gxh	A	125	-145,5	-137,7
PF04273	A4Y1H6	3gxg	C	125	-145,6	-135,9
PF04273	A4Y1H6	3gxg	B	125	-146	-137,9
PF04273	A4Y1H6	3gxh	B	125	-143,5	-135,4
PF04273	A4Y1H6	3gxg	A	125	-146,9	-137,5
PF04273	A4Y1H6	3gxg	D	125	-144,5	-135,9
PF13354	A8DS27	3dw0	B	69	47,2	-144,5
PF13354	A8DS27	3c5a	A	69	51,4	-146
PF13354	A8DS27	3dw0	A	69	46,1	-142,5
PF13354	P0C5C1	3n6i	A	83	43,8	-144,2
PF13354	P0C5C1	3m6b	A	83	46,3	-139,7
PF13354	P0C5C1	3dwz	A	83	39,8	-139,9
PF13354	P0C5C1	3n8r	A	83	44,6	-141,7
PF13354	P0C5C1	3n7w	A	83	45,4	-146,7
PF13354	P0C5C1	3cg5	A	83	44	-133,2
PF13354	P0C5C1	3ndg	A	83	47,3	-147,2
PF13354	P0C5C1	3n8l	A	83	44,7	-143,3
PF13354	P0C5C1	3nbl	A	83	46,6	-142,2
PF13354	P0C5C1	3iqa	A	83	46,6	-145,1
PF13354	P0C5C1	3ny4	A	83	45,8	-144,1
PF13354	P0C5C1	3m6h	A	83	42,6	-140,4
PF13354	P0C5C1	3nde	A	83	45,6	-144,5
PF13354	P0C5C1	3nc8	A	83	44	-140
PF13354	P0C5C1	3n8s	A	83	47,5	-143,6
PF13354	P22391	3byd	A	72	51,4	-139,3
PF13354	Q47066	2zqc	A	69	49,3	-139,7
PF13354	Q47066	2xqz	A	69	56,4	-135,7
PF13354	Q47066	2wyx	A	69	54,1	-148,8
PF13354	Q47066	1we4	A	69	57,2	-142,8
PF13354	Q47066	2zqa	A	69	51,1	-141,2
PF13354	Q47066	2zq8	A	69	51,7	-139,3
PF13354	Q47066	1iyo	A	69	57,1	-143,2
PF13354	Q47066	2xr0	A	69	49,6	-137,4

PF13354	Q47066	2zqd	A	69	49,4	-140
PF13354	Q47066	1bza	A	69	56,3	-135
PF13354	Q47066	2zq9	A	69	50,5	-140,3
PF13354	Q47066	2zq7	A	69	52,4	-144,2
PF13354	Q47066	1iyq	A	69	56,7	-145,3
PF13354	Q47066	1iyp	A	69	60,6	-141,4
PF13354	Q47066	1iys	A	69	54,3	-141,5
PF13354	Q5NH60	3p09	A	60	55	-145,8
PF13354	Q5NH60	3p09	B	60	55,5	-145
PF13354	Q840M4	1ylp	A	69	46	-134,8
PF13354	Q939N4	1ylw	A	69	58	-140,8
PF13354	Q93F76	3nia	A	63	37,9	-134,7
PF13354	Q93F76	3ni9	A	63	52,8	-146
PF13354	Q93F76	3ni9	B	63	46,9	-138,3
PF13354	Q93LQ9	2ov5	B	69	51,7	-136
PF13354	Q93LQ9	3e2k	A	69	49,2	-144,3
PF13354	Q93LQ9	3e2k	B	69	54,6	-142,2
PF13354	Q93LQ9	2ov5	A	69	54,1	-134,9
PF13354	Q93LQ9	2ov5	C	69	47,3	-141,3
PF13354	Q93PQ0	3bfe	A	69	60,5	-135,8
PF13354	Q93PQ0	3bff	C	69	55,5	-137,4
PF13354	Q93PQ0	3bff	D	69	52,6	-138
PF13354	Q93PQ0	3bfg	C	69	53,1	-138,1
PF13354	Q93PQ0	3bfg	A	69	53,7	-139,9
PF13354	Q93PQ0	3bfd	C	69	54,5	-142,8
PF13354	Q93PQ0	3bfc	C	69	53	-137,4
PF13354	Q93PQ0	3bff	A	69	56,5	-137,1
PF13354	Q93PQ0	3bfe	C	69	63,2	-132
PF13354	Q93PQ0	3bfd	D	69	54,2	-142,6
PF13354	Q93PQ0	3bfd	B	69	54,3	-141,4
PF13354	Q93PQ0	3bfc	A	69	34,4	-126,1
PF13354	Q93PQ0	3bff	B	69	55,7	-137,3
PF13354	Q93PQ0	3bfe	D	69	59,8	-135,5
PF13354	Q93PQ0	3bfc	D	69	52,6	-136,8
PF13354	Q93PQ0	3bfc	B	69	55,6	-137,8
PF13354	Q93PQ0	3bfg	D	69	53,1	-140,6
PF13354	Q93PQ0	3bfd	A	69	55,6	-140,9
PF13354	Q93PQ0	3bfe	B	69	61,4	-131,5
PF13354	Q93PQ0	3bfg	B	69	55,9	-139,5
PF13354	Q9KJY7	2qpn	A	63	55,4	-144
PF13354	Q9KJY7	2qpn	B	63	53,2	-148,7
PF13354	Q9L5C7	1ylz	A	69	47	-140,2
PF13354	Q9L5C7	1ylt	A	69	46,6	-136,8
PF13354	Q9L5C7	1ylz	B	69	46,2	-139,8
PF13354	Q9L5C7	1yly	B	69	47,7	-139,6
PF13354	Q9L5C7	1yly	A	69	45,8	-139,2
PF13354	Q9L5C8	3g2z	A	69	48,8	-139,9
PF13354	Q9L5C8	3q07	B	69	50,8	-137,7
PF13354	Q9L5C8	1ymx	B	69	51,7	-142,9
PF13354	Q9L5C8	2p74	B	69	50,9	-141
PF13354	Q9L5C8	2p74	A	69	50,8	-140,4
PF13354	Q9L5C8	3q1f	A	69	49,7	-138,3
PF13354	Q9L5C8	3g2y	B	69	49,3	-138,9
PF13354	Q9L5C8	3g35	B	69	46,9	-140,8

PF13354	Q9L5C8	3q1f	B	69	48,4	-137,3
PF13354	Q9L5C8	3huo	A	69	50,7	-140,7
PF13354	Q9L5C8	3g34	B	69	50	-139,6
PF13354	Q9L5C8	1ym1	B	69	48,5	-137,9
PF13354	Q9L5C8	3hre	A	69	54,3	-139,8
PF13354	Q9L5C8	3hlw	A	69	49,8	-136,8
PF13354	Q9L5C8	1ym1	A	69	47,8	-139,3
PF13354	Q9L5C8	3g35	A	69	45,9	-140,9
PF13354	Q9L5C8	3g2z	B	69	48,5	-140,9
PF13354	Q9L5C8	3g34	A	69	47,8	-139
PF13354	Q9L5C8	1ymx	A	69	50,7	-139,7
PF13354	Q9L5C8	1yms	B	69	52,1	-142,3
PF13354	Q9L5C8	3huo	B	69	49,7	-140,2
PF13354	Q9L5C8	3q07	A	69	51,3	-134,8
PF13354	Q9L5C8	1yms	A	69	55,2	-141,5
PF13354	Q9L5C8	3hlw	B	69	48,3	-137,6
PF13354	Q9L5C8	3g2y	A	69	49,1	-138,6
PF13354	Q9L5C8	3g31	B	69	49,8	-139,8
PF13354	Q9L5C8	3hvf	B	69	49,8	-140,3
PF13354	Q9L5C8	3hvf	A	69	50,5	-139,9
PF13354	Q9L5C8	3hre	B	69	53,3	-140,4
PF13354	Q9L5C8	3g32	A	69	48,9	-139,2
PF13354	Q9L5C8	3g30	A	69	50,1	-138,8
PF13354	Q9L5C8	1ylj	A	69	49,8	-139,1
PF13354	Q9L5C8	3g31	A	69	46,9	-136,8
PF13354	Q9L5C8	3g32	B	69	48,7	-139,6
PF13354	Q9RBQ1	1o7e	B	69	49,8	-141,1
PF13354	Q9RBQ1	1n4o	B	69	46,9	-142,6
PF13354	Q9RBQ1	1o7e	A	69	48,9	-143,5
PF13354	Q9RBQ1	1o7e	B	220	-100,9	-128,9
PF13354	Q9RBQ1	1n4o	B	220	-104,3	-127,4
PF13354	Q9RBQ1	1n4o	A	69	49,7	-142,9
PF13354	Q9RBQ1	1o7e	A	220	-101,9	-130,1
PF13354	Q9RBQ1	1n4o	A	220	-99,7	-127,8
PF00077	Q7SPG9	2fdd	B	67	63	-113,2
PF00077	O38732	2nxl	A	67	55,8	-124,1
PF00077	P03366	1mes	A	67	51,6	-137,6
PF00077	P03366	1mes	B	67	47,5	-132,6
PF00077	P03366	1mer	A	67	54,3	-137
PF00077	P03366	1met	B	67	45,2	-141,6
PF00077	P03366	1met	A	67	53,6	-138,8
PF00077	P03366	1meu	B	67	44,5	-148,3
PF00077	P03367	1zj7	A	67	56	-125,6
PF00077	P03369	3em3	A	67	60,4	-128,5
PF00077	P04585	1bv9	B	67	46,8	-128,4
PF00077	P04585	1bv7	A	67	55,4	-130,9
PF00077	P04585	1bwb	B	67	48,6	-137,8
PF00077	P04585	1bv7	B	67	51,2	-137,7
PF00077	P04585	1qbu	B	67	53,3	-97,5
PF00077	P04585	1bwa	B	67	49,4	-137,9
PF00077	P04585	1qbu	A	67	64,4	-103,9
PF00077	P04585	1qbr	B	67	50,9	-140,9
PF00077	P04585	1hvh	B	67	50,7	-135,5
PF00077	P04585	3kt5	A	67	55,4	-135

PF00077	P04587	1hpv	A	67	63,5	-129,5
PF00077	Q993Q5	3u7s	B	67	57,2	-121,4
PF00077	Q9WEZ1	3ixo	A	67	61,9	-128,3
PF00795	D0VWZ1	3hkx	A	165	41	-116,4
PF00795	O58376	1j31	D	146	36,1	-114
PF00795	O58376	1j31	C	146	38,4	-115
PF00795	O58376	1j31	A	146	39,2	-117,3
PF00795	O58376	1j31	B	146	35,7	-115,7
PF00795	P0A5L6	3dla	B	176	34,8	-95,5
PF00795	P0A5L6	3dla	D	176	32,7	-94,8
PF00795	P0A5L6	3dla	C	176	32,2	-96,2
PF00795	P0A5L6	3dla	A	176	33	-106,4
PF00795	Q5HEG7	3p8k	B	146	33,9	-103,1
PF00795	Q5HEG7	3p8k	A	146	35,1	-101,3
PF00795	Q9JHW2	2w1v	A	191	41,9	-103
PF00795	Q9JHW2	2w1v	B	191	44,3	-102,7
PF00235	P35080	1d1j	C	25	-109,2	-111,6
PF00235	P35080	1d1j	B	25	-101	-108,7
PF00235	P35080	1d1j	A	25	-119,9	-100,7
PF00235	P35080	1d1j	D	25	-109,9	-107,5
PF00235	Q3V171	2v8f	A	25	-105,7	-108,8
PF00235	Q3V171	2v8f	B	25	-113,3	-108,9
PF00235	Q9EPC6	2vk3	A	25	-117,6	-108,7
PF00069	P53779	3da6	A	117	-44,3	-94,3
PF00069	P63086	2fys	B	252	-62,9	-148,3
PF00069	Q16539	3ctq	A	119	-75,9	-114,1
PF01058	F2Z6J5	3ayx	B	126	49,2	-149,8
PF01058	F2Z6J5	3ayy	D	126	47,1	-147
PF01058	P31892	3rgw	S	120	48,6	-148,2
PF01451	P45947	1z2e	A	15	-134,3	-93,8
PF01451	Q5SJ34	2cwd	B	16	-121,7	-90
PF01134	O66962	2zxi	C	48	-123,3	-127,2
PF01134	O66962	2zxi	B	48	-123	-128,1
PF01134	O66962	2zxi	D	48	-119,2	-123,2
PF01134	O66962	2zxi	A	48	-119,8	-123,6
PF01134	P0A6U3	3ces	A	47	-119,2	-132,7
PF01134	P0A6U3	3ces	D	47	-103,7	-120,1
PF01134	P0A6U3	3ces	B	47	-111,9	-131,4
PF01134	P0A6U3	3ces	C	47	-114,8	-135,3
PF01301	P16278	3thc	B	127	66,2	-101,8
PF01301	P16278	3thd	B	127	63,3	-99,7
PF01301	P16278	3thc	A	127	62,6	-104,9
PF01301	P16278	3thd	D	127	67,3	-106
PF01301	P16278	3thc	D	127	69,6	-104,7
PF01301	P16278	3thc	C	127	66,5	-108,1
PF01301	P16278	3thd	C	127	64,7	-103,2
PF01301	P16278	3thd	A	127	64,1	-102,7
PF01301	Q8AB22	3d3a	A	121	65,2	-98,1
PF00104	P03372	2iog	A	530	80	-147,1
PF00104	P03372	1yim	A	530	-112,9	-126,9
PF00104	P03372	1xp1	A	530	26,2	-91
PF00104	P15207	1i37	A	844	-178,6	-115,3
PF11550	Q5NEC5	2qwu	A	194	-97	-132,9
PF11550	Q5NEC5	2qwu	B	194	-95,1	-135,3

PF12160	P02672	2baf	A	51	50,8	-100,7
PF01327	P0A6K3	2def	A	90	-92,5	-124,2
PF02146	O28597	1m2k	A	148	-123,8	-142,2
PF00320	P17429	4gat	A	33	-85,7	-145,5
PF00320	P17429	5gat	A	33	-86,9	-138,9
PF01432	P52888	2o36	A	350	64,6	-110,2
PF01536	P17707	3dz5	A	82	-135,6	-142,1
PF00240	Q9BZL1	1p0r	A	18	-146	-149,5
PF13894	O35615	1srk	A	13	-95,2	-128,5
PF13419	O31156	2iof	A	22	70,1	-104,1
PF02033	P75589	1pa4	A	53	-71,1	-148,7
PF10013	Q5SHP9	3kit	Y	79	-120,7	-148,2
PF00443	Q93009	1nb8	B	223	51,3	-104,5
PF00443	Q93009	1nb8	A	223	65,1	-120,9
PF00443	Q93009	1nbf	E	223	58,6	-110,2
PF13499	P79880	2r2i	A	124	-34,8	-102,1
PF01123	Q5HHK0	3ea6	A	73	47,9	-114,2
PF02810	Q4FPZ7	2i9w	A	28	-130,8	-94,3
PF02733	Q9CIW0	2iu6	B	298	43,5	-120,4
PF02733	Q9CIW0	2iu4	A	298	45,7	-123,9
PF02733	Q9CIW0	2iu4	B	298	43,7	-126,6
PF03718	P48845	1ogo	X	415	-87,9	-105,3
PF03718	P48845	1ogm	X	415	-89,5	-104,9
PF00133	P56690	1jzs	A	464	-86,2	-114,7
PF00254	Q14318	2awg	A	178	-115,5	-119,5
PF00254	Q14318	3ey6	A	121	-117,8	-112,9
PF00180	O14104	3ty3	B	131	63,7	-141,4
PF00180	O14104	3ty3	A	131	66,9	-142,2
PF00180	O14104	3ty4	A	131	62,4	-140,8
PF00180	O14104	3ty4	B	131	60,1	-142,3
PF00498	P66799	2fez	A	372	53,2	-114,3
PF00498	P66799	2ff4	A	372	56	-113,5
PF03331	D5CV28	3ps1	A	63	60,2	-141,2
PF03331	D5CV28	3ps2	A	63	56,9	-145,3
PF03331	D5CV28	3p3g	A	63	61,1	-146,5
PF03331	D5CV28	3ps3	A	63	58,9	-142,8
PF03643	Q8C0E2	3lh9	A	171	-106,8	-102,4
PF13465	P25490	1ubd	C	360	-86,3	-90,3
PF00403	O32220	1opz	A	17	51,3	-136,3
PF01636	P0A3Y5	3tm0	A	156	65,6	-113,4
PF02597	O32583	1zud	2	12	-124,3	-97,6
PF10431	P43773	1ofh	A	263	-106,8	-144,9
PF02217	P03070	2ntc	A	216	-58,5	-149,4
PF04879	Q56223	3iam	C	263	-67,2	-146
PF04879	Q56223	3iam	3	263	-67,2	-143,4
PF01780	P60619	1vq9	Z	42	-100,8	-148,7
PF01780	P60619	1yhq	Z	42	-75	-128,7
PF01780	P60619	1vqo	Z	42	-85,3	-140,5
PF01780	P60619	1vqk	Z	42	-85,8	-138,7
PF01780	P60619	1vqm	Z	42	172,8	-135,6
PF01780	P60619	1vql	Z	42	175,6	-136,7
PF01780	P60619	1vqp	Z	42	-91,2	-107,7
PF01780	P60619	3cc2	Z	66	-80	-125,2
PF00793	P0A715	1phw	A	1249	-140,9	-112,8

PF00018	P62993	1gfd	A	42	-145	-94,3
PF00076	Q9Y3B4	2f9d	B	83	47,5	-145,3
PF00079	P05543	2riw	A	66	51	-137,7
PF00079	P05543	2xn3	A	66	55,8	-133,6
PF00079	P05543	2riv	A	66	49,7	-139,9
PF03114	Q62420	1zww	A	108	-114,7	-104,7
PF06393	P70444	1ddb	A	30	-56,1	-110
PF04135	Q8U1R4	2ey4	F	23	-74,8	-145,9
PF00144	Q9ZBA9	1ei5	A	61	48,8	-124,6
PF02458	A4PHY4	2e1u	A	421	-82,6	-90,8
PF02458	A4PHY4	2e1u	B	421	-81,6	-113,2
PF00579	P00951	1wq3	A	116	-146,7	-149,4
PF02745	P11558	3m32	A	218	-114	-124,7
PF02745	P11558	1mro	A	218	-113,7	-126,8
PF02745	P11558	3m1v	A	218	-111,4	-126,9
PF02745	P11558	3m30	D	218	-108,2	-123,1
PF02745	P11558	1hbm	D	218	-113,9	-123,4
PF02745	P11558	1hbo	D	218	-109,8	-122,9
PF02745	P11558	3m1v	D	218	-109,6	-127,6
PF02745	P11558	3pot	D	218	-112,4	-126,5
PF02745	P11558	1hbn	D	218	-112,6	-124,8
PF02745	P11558	3m2u	D	218	-111,4	-125,6
PF02745	P11558	3m30	A	218	-114,7	-124
PF02745	P11558	1hbm	A	218	-114	-125,7
PF02745	P11558	3m2r	A	218	-114,5	-126,7
PF02745	P11558	1mro	D	218	-113	-124,9
PF02745	P11558	1hbo	A	218	-111,5	-125,2
PF02745	P11558	3pot	A	218	-111,9	-124,5
PF02745	P11558	1hbu	A	218	-113,1	-123,1
PF02745	P11558	3m2v	D	218	-109,5	-126,1
PF02745	P11558	1hbu	D	218	-108,9	-126,5
PF02745	P11558	3m32	D	218	-110,4	-125,9
PF02745	P11558	3m2v	A	218	-109,8	-124,6
PF02745	P11558	3m2u	A	218	-115,1	-125,7
PF02745	P11558	3m2r	D	218	-109,3	-127,3
PF02745	P11558	1hbn	A	218	-109,5	-132,3
PF00042	P68871	1bij	B	93	-93,7	-100,9
PF07653	Q96B97	2k6d	A	301	-64,1	-133,5
PF00680	Q82122	1tp7	A	229	-165,3	-147,7
PF00685	P52839	2q3m	A	155	-65,6	-119,1
PF01474	O53512	3rzi	B	440	-154	-106,5
PF01474	O53512	2w1a	A	440	-151,6	-105,6
PF01474	O53512	2w1a	B	440	-145,8	-104,7
PF01474	O53512	3kgf	A	440	-146,6	-118,2
PF01474	O53512	3rzi	A	440	-143,5	-108,6
PF01474	O53512	3pfp	B	440	-154,6	-102,1
PF01474	O53512	3nv8	A	440	-152,2	-110
PF01474	O53512	3nv8	B	440	-145,9	-104
PF01926	Q9X1F8	1mky	A	57	47,1	-115,3
PF01641	Q78J03	2l1u	A	43	-110	-103,5
PF12888	P23890	3lya	A	208	-17,2	-124,9
PF00936	B1VB78	3pac	A	38	45	-149,7
PF00175	P83686	1ndh	A	255	-83,2	-149,8
PF00217	Q6AW42	3l2f	O	292	-117,1	-146,9

PF02979	Q7SID2	1ugs	A	111	-171,1	-98,3
PF02979	Q7SID2	1ugq	A	111	-163,5	-95,5
PF01182	B8E528	3nwp	A	143	46,9	-137,1
PF01182	B8E528	3nwp	B	143	49,7	-134,4
PF10569	P01024	3g6j	B	988	-99,6	-109,7
PF00696	Q60352	3k56	A	230	-124,1	-147,2
PF03171	P18548	1w28	A	155	-134	-140,6
PF03171	P18548	1rxg	A	155	-134	-141,3
PF03171	P18548	2jb8	A	155	-134,8	-141,4
PF03171	P18548	1uo9	A	155	-131,9	-143,9
PF03171	P18548	1e5h	A	155	-120,7	-139,3
PF03171	P18548	1uob	A	155	-133,2	-140,8
PF03171	P18548	1dcs	A	155	-134,5	-140,4
PF03171	P18548	1e5i	A	155	-118,2	-142,3
PF03171	P18548	1hjg	A	155	-122,1	-140,8
PF05592	Q93RE7	2okx	A	896	54,9	-121,6
PF05592	Q93RE7	2okx	B	896	57	-121,5
PF00596	Q9RQ12	2opi	B	25	53,5	-116,2
PF00596	Q9RQ12	2opi	A	25	47,9	-123,5
PF04909	C8N232	3nur	A	219	-97,7	-121,3
PF08601	P19880	1sse	B	598	-59	-105,4
PF00607	P14077	1g03	A	63	-107,3	-143
PF07925	P17378	1n35	A	1064	-124,5	-138,2
PF07925	P17378	1mwh	A	1064	-120	-141,4
PF07925	P17378	1muk	A	1064	-121,1	-138,4
PF04199	Q58193	2b0a	A	42	58,9	-139,1
PF01179	P46881	3amo	B	315	67,7	-92,7
PF07714	P43403	1u59	A	346	-157	-144,9
PF02901	P09373	1h18	A	418	61	-112,8
PF02901	P09373	1h18	B	418	62,6	-106,8
PF02901	P09373	1h16	A	418	58,5	-109,9
PF02901	P09373	1h17	A	418	61,2	-104,4
PF03372	A7LAI8	3i41	A	122	-120,8	-132,6
PF00516	Q8QDX5	2ny4	A	268	-117,8	-99,9
PF00186	Q27793	3irm	A	187	-97,7	-149,3
PF00225	Q02224	1t5c	A	144	-123,6	-132,5
PF00022	P68135	3b5u	B	285	-149,6	-146,7
PF00022	P68135	3b5u	F	285	-156,7	-101,5
PF05996	Q58MU6	2vgr	D	159	-136,5	-126,7
PF00027	P00516	3shr	B	174	-71,6	-114,5
PF03063	Q9F8A8	3b51	X	201	-116	-121,2
PF03063	Q9F8A8	1su7	A	201	-111,2	-124
PF03063	Q9F8A8	1suf	A	201	-112	-123,6
PF03063	Q9F8A8	1su6	A	201	-115,2	-122,7
PF03063	Q9F8A8	1su8	A	201	-112,8	-124,7
PF03063	Q9F8A8	3b53	X	201	-115,7	-121,5
PF03063	Q9F8A8	3b52	X	201	-117,3	-123,5
PF03063	Q9F8A8	3i39	X	201	-111,4	-122,1
PF00367	P69786	1o2f	B	335	-135,2	-130,9
PF00367	P69786	3bp3	B	33	-109,6	-131,9
PF00367	P69786	3bp3	A	33	-110	-132
PF00613	O02697	1e7u	A	524	-68,9	-122
PF00425	Q81QQ0	3os6	C	306	-123,4	-93,1
PF00425	Q81QQ0	3os6	B	306	-125,8	-92

PF00425	Q81QQ0	3os6	D	306	-117,5	-98,3
PF00425	Q81QQ0	3os6	A	306	-115,5	-96,7
PF07823	O04147	1jh7	A	86	-89,1	-92
