# Supplementary material for: Protein Topology Determines Cysteine Oxidation Fate: The Case of Sulfenyl Amide Formation among Protein Families
Source: PLoS Comput Biol. 2015 Mar 5;11(3):e1004051. doi: 10.1371/journal.pcbi.1004051 (PMC4351059; doi:10.1371/journal.pcbi.1004051)
Supplement: S3 Table — (PDF) [file pcbi.1004051.s014.pdf]

Table S3. Proteins with cysteine in "forbidden conformation" and without the beta-loop-helix motif.

PFAM Family	UniProt	PDB	Chain	ResID	Phi	Psi
PF00018	P62993	1gfd	A	42	-145	-94,3
PF00022	P68135	3b5u	B	285	-149,6	-146,7
PF00027	P00516	3shr	B	174	-71,6	-114,5
PF00042	P68871	1bij	B	93	-93,7	-100,9
PF00069	P53779	3da6	A	117	-44,3	-94,3
PF00069	P63086	2fys	B	252	-62,9	-148,3
PF00069	Q16539	3ctq	A	119	-75,9	-114,1
PF00076	Q9Y3B4	2f9d	B	83	47,5	-145,3
PF00077	O38732	2nxl	A	67	55,8	-124,1
PF00077	P03366	1mes	A	67	51,6	-137,6
PF00077	P03367	1zj7	A	67	56	-125,6
PF00077	P03369	3em3	A	67	60,4	-128,5
PF00077	P04585	1bv9	B	67	46,8	-128,4
PF00077	P04587	1hpv	A	67	63,5	-129,5
PF00077	Q7SPG9	2fdd	B	67	63	-113,2
PF00077	Q993Q5	3u7s	B	67	57,2	-121,4
PF00077	Q9WEZ1	3ixo	A	67	61,9	-128,3
PF00079	P05543	2riw	A	66	51	-137,7
PF00104	P03372	2iog	A	530	80	-147,1
PF00104	P15207	1i37	A	844	-178,6	-115,3
PF00133	P56690	1jzs	A	464	-86,2	-114,7
PF00144	Q9ZBA9	1ei5	A	61	48,8	-124,6
PF00175	P83686	1ndh	A	255	-83,2	-149,8
PF00180	O14104	3ty3	B	131	63,7	-141,4
PF00186	Q27793	3irm	A	187	-97,7	-149,3
PF00217	Q6AW42	3l2f	O	292	-117,1	-146,9
PF00225	Q02224	1t5c	A	144	-123,6	-132,5
PF00235	P35080	1d1j	C	25	-109,2	-111,6
PF00235	Q3V171	2v8f	A	25	-105,7	-108,8
PF00235	Q9EPC6	2vk3	A	25	-117,6	-108,7
PF00240	Q9BZL1	1p0r	A	18	-146	-149,5
PF00254	Q14318	2awg	A	178	-115,5	-119,5
PF00320	P17429	4gat	A	33	-85,7	-145,5
PF00367	P69786	1o2f	B	335	-135,2	-130,9
PF00403	O32220	1opz	A	17	51,3	-136,3
PF00425	Q81QQ0	3os6	C	306	-123,4	-93,1
PF00443	Q93009	1nb8	B	223	51,3	-104,5
PF00498	P66799	2fez	A	372	53,2	-114,3
PF00516	Q8QDX5	2ny4	A	268	-117,8	-99,9
PF00579	P00951	1wq3	A	116	-146,7	-149,4
PF00596	Q9RQ12	2opi	B	25	53,5	-116,2
PF00607	P14077	1g03	A	63	-107,3	-143
PF00613	O02697	1e7u	A	524	-68,9	-122
PF00680	Q82122	1tp7	A	229	-165,3	-147,7
PF00685	P52839	2q3m	A	155	-65,6	-119,1
PF00696	Q60352	3k56	A	230	-124,1	-147,2
PF00793	P0A715	1phw	A	1249	-140,9	-112,8
PF00795	D0VWZ1	3hkx	A	165	41	-116,4

PF00795	O58376	1j31	D	146	36,1	-114
PF00795	P0A5L6	3dla	B	176	34,8	-95,5
PF00795	Q5HEG7	3p8k	B	146	33,9	-103,1
PF00795	Q9JHW2	2w1v	A	191	41,9	-103
PF00936	B1VB78	3pac	A	38	45	-149,7
PF01058	F2Z6J5	3ayx	B	126	49,2	-149,8
PF01058	P31892	3rgw	S	120	48,6	-148,2
PF01123	Q5HHK0	3ea6	A	73	47,9	-114,2
PF01134	O66962	2zxi	C	48	-123,3	-127,2
PF01134	P0A6U3	3ces	A	47	-119,2	-132,7
PF01179	P46881	3amo	B	315	67,7	-92,7
PF01182	B8E528	3nwp	A	143	46,9	-137,1
PF01301	P16278	3thc	B	127	66,2	-101,8
PF01301	Q8AB22	3d3a	A	121	65,2	-98,1
PF01327	P0A6K3	2def	A	90	-92,5	-124,2
PF01432	P52888	2o36	A	350	64,6	-110,2
PF01451	P45947	1z2e	A	15	-134,3	-93,8
PF01451	Q5SJ34	2cwd	B	16	-121,7	-90
PF01474	O53512	3rzi	B	440	-154	-106,5
PF01536	P17707	3dz5	A	82	-135,6	-142,1
PF01636	P0A3Y5	3tm0	A	156	65,6	-113,4
PF01641	Q78J03	2l1u	A	43	-110	-103,5
PF01780	P60619	1vq9	Z	42	-100,8	-148,7
PF01926	Q9X1F8	1mky	A	57	47,1	-115,3
PF02033	P75589	1pa4	A	53	-71,1	-148,7
PF02146	O28597	1m2k	A	148	-123,8	-142,2
PF02217	P03070	2ntc	A	216	-58,5	-149,4
PF02458	A4PHY4	2e1u	A	421	-82,6	-90,8
PF02597	O32583	1zud	2	12	-124,3	-97,6
PF02733	Q9CIW0	2iu6	B	298	43,5	-120,4
PF02745	P11558	3m32	A	218	-114	-124,7
PF02810	Q4FPZ7	2i9w	A	28	-130,8	-94,3
PF02901	P09373	1h18	A	418	61	-112,8
PF02979	Q7SID2	1ugs	A	111	-171,1	-98,3
PF03063	Q9F8A8	3b51	X	201	-116	-121,2
PF03114	Q62420	1zww	A	108	-114,7	-104,7
PF03171	P18548	1w28	A	155	-134	-140,6
PF03331	D5CV28	3ps1	A	63	60,2	-141,2
PF03372	A7LAI8	3i41	A	122	-120,8	-132,6
PF03643	Q8C0E2	3lh9	A	171	-106,8	-102,4
PF03718	P48845	1ogo	X	415	-87,9	-105,3
PF04135	Q8U1R4	2ey4	F	23	-74,8	-145,9
PF04199	Q58193	2b0a	A	42	58,9	-139,1
PF04879	Q56223	3iam	C	263	-67,2	-146
PF04909	C8N232	3nur	A	219	-97,7	-121,3
PF05592	Q93RE7	2okx	A	896	54,9	-121,6
PF05996	Q58MU6	2vgr	D	159	-136,5	-126,7
PF06393	P70444	1ddb	A	30	-56,1	-110
PF07653	Q96B97	2k6d	A	301	-64,1	-133,5
PF07714	P43403	1u59	A	346	-157	-144,9
PF07823	O04147	1jh7	A	86	-89,1	-92

PF07925	P17378	1n35	A	1064	-124,5	-138,2
PF08601	P19880	1sse	B	598	-59	-105,4
PF10013	Q5SHP9	3kit	Y	79	-120,7	-148,2
PF10431	P43773	1ofh	A	263	-106,8	-144,9
PF10569	P01024	3g6j	B	988	-99,6	-109,7
PF11550	Q5NEC5	2qwu	A	194	-97	-132,9
PF12160	P02672	2baf	A	51	50,8	-100,7
PF12888	P23890	3lya	A	208	-17,2	-124,9
PF13354	A8DS27	3dw0	B	69	47,2	-144,5
PF13354	P0C5C1	3n6i	A	83	43,8	-144,2
PF13354	P22391	3byd	A	72	51,4	-139,3
PF13354	Q47066	2zqc	A	69	49,3	-139,7
PF13354	Q5NH60	3p09	A	60	55	-145,8
PF13354	Q840M4	1ylp	A	69	46	-134,8
PF13354	Q939N4	1ylw	A	69	58	-140,8
PF13354	Q93F76	3nia	A	63	37,9	-134,7
PF13354	Q93LQ9	2ov5	B	69	51,7	-136
PF13354	Q93PQ0	3bfe	A	69	60,5	-135,8
PF13354	Q9KJY7	2qpn	A	63	55,4	-144
PF13354	Q9L5C7	1ylz	A	69	47	-140,2
PF13354	Q9L5C8	3g2z	A	69	48,8	-139,9
PF13354	Q9RBQ1	1o7e	B	69	49,8	-141,1
PF13419	O31156	2iof	A	22	70,1	-104,1
PF13465	P25490	1ubd	C	360	-86,3	-90,3
PF13499	P79880	2r2i	A	124	-34,8	-102,1
PF13894	O35615	1srk	A	13	-95,2	-128,5
