# Supplementary material for: Protein Topology Determines Cysteine Oxidation Fate: The Case of Sulfenyl Amide Formation among Protein Families
Source: PLoS Comput Biol. 2015 Mar 5;11(3):e1004051. doi: 10.1371/journal.pcbi.1004051 (PMC4351059; doi:10.1371/journal.pcbi.1004051)
Supplement: S4 Table — (PDF) [file pcbi.1004051.s015.pdf]

Table S4. Proteins in the PDB in forbidden conformation Cys with the beta-loop-helix motif.

PFAM Family	UniProt	PDB	Chain	ResID	Phi	Psi
PF00102	A3QMF6	3f41	B	250	-128,2	-124,5
PF00102	M0R726	2nv5	C	1424	-132,5	-121,7
PF00102	O14522	2ooq	A	1106	-129,4	-122,9
PF00102	O68720	1qz0	A	403	-124,5	-118,4
PF00102	P10586	1lar	A	1813	-140	-124,2
PF00102	P15273	1xxv	A	403	-117,7	-114,1
PF00102	P18031	2zmm	A	215	-122,1	-129,8
PF00102	P18052	1p15	B	724	-134,4	-97,3
PF00102	P23467	2i4g	A	1904	-126	-125,3
PF00102	P23470	3qci	A	1060	-120,9	-116,7
PF00102	P26045	2b49	A	842	-129,4	-137,3
PF00102	P28827	1rpm	B	1095	-125,3	-124,9
PF00102	P29074	2i75	A	852	-122,1	-121,2
PF00102	P35992	3s3e	B	242	-119,6	-125,9
PF00102	P74873	1g4w	R	481	-118,8	-129,8
PF00102	Q06124	2shp	A	459	-133,2	-127,5
PF00102	Q12923	1wch	A	2408	-143,2	-122,6
PF00102	Q13332	2fh7	A	1880	-123	-117
PF00102	Q15256	2a8b	A	588	-126,5	-110,6
PF00102	Q15262	2c7s	A	1083	-123,1	-119
PF00102	Q15678	2bzl	A	1121	-133,7	-124,1
PF00102	Q16827	2gjt	A	1136	-127,7	-115,6
PF00102	Q16849	2i1y	A	909	-129,7	-114,9
PF00102	Q3MIV7	2h04	A	1904	-133,1	-125,9
PF00102	Q59EE0	2nlk	A	1060	-135,4	-124
PF00102	Q62132	1jln	A	480	-135,5	-122,9
PF00102	Q62884	3i36	A	1118	-127,5	-120,5
PF00102	Q7ARH8	2y2f	A	403	-115,9	-122
PF00102	Q7WUJ1	3d1o	B	252	-130,8	-138,4
PF00102	Q92932	2qep	B	945	-142,9	-102,9
PF00102	Q97VZ7	2i6j	A	96	-129,9	-132,7
PF00102	Q99952	2oc3	A	229	-129,7	-139,2
PF00102	Q9Y2R2	2p6x	B	227	-125,2	-138
PF00106	A4VVQ2	3cxr	A	149	-93,5	-139,6
PF00106	P10807	1b16	B	137	-99,5	-114,8
PF00106	Q48436	1geg	A	138	-110,1	-141,8
PF00117	O59071	1wl8	A	79	57,9	-102,4
PF00117	P00900	1i7q	B	85	57,3	-94,5
PF00117	P00905	1i1q	B	83	59,9	-115
PF00117	P00907	1jdb	F	269	58,3	-107,7
PF00117	P04079	1gpm	C	86	56,8	-111,8
PF00117	P0A6F1	1bxr	H	269	55	-97,4
PF00117	P0A7E5	1s1m	A	379	52,1	-114,1
PF00117	P33734	1ox6	B	83	58,2	-111,8
PF00117	P49915	2vpi	A	104	56,2	-108,7
PF00117	Q06129	1qdl	B	84	60,5	-110,7
PF00117	Q1H479	3m3p	A	89	58,2	-114,4
PF00117	Q5SI28	2ywc	C	78	50,5	-106,3
PF00117	Q5SIA8	1vcm	A	391	52,4	-106,2
PF00117	Q7SIC0	1ka9	H	82	55,8	-100,4
PF00117	Q980S6	3nva	B	386	59,3	-111,3

PF00117	Q9HJM3	2a9v	B	80	59,2	-109,3
PF00117	Q9NRF8	2vkt	A	399	65,9	-110,9
PF00117	Q9X0C8	1gpw	B	84	67	-132,9
PF00142	P00459	1g20	E	151	-143,6	-149,5
PF00162	P00560	3pgk	A	97	-130,2	-99
PF00300	P07953	1tip	A	142	-128,5	-137,3
PF00300	P16118	1k6m	B	391	-133	-128,5
PF00300	P25114	1bif	A	389	-132,9	-134,3
PF00300	Q16875	2axn	A	386	-133,1	-134,8
PF00384	P81186	2nap	A	307	-139,2	-135,9
PF00581	E2QRA0	3p3a	B	246	-144,3	-126,5
PF00581	O05793	3hwi	A	233	-131,3	-137,6
PF00581	P00586	1orb	A	247	-161	-128,6
PF00581	P25325	3olh	A	248	-165,2	-148,8
PF00581	P30304	1c25	A	430	-134,4	-128,6
PF00581	P30305	2ifd	A	473	-135,9	-140,3
PF00581	P42937	3f4a	A	90	-130,2	-137,4
PF00581	P52197	1h4m	X	230	-139,7	-125,3
PF00581	P78067	3ipp	B	385	-143,8	-130,2
PF00581	P96888	3hzu	A	245	-143,1	-119,4
PF00581	Q12305	3d1p	A	98	-134,3	-124,1
PF00581	Q5SHV8	2eg4	A	191	-157,2	-131,1
PF00581	Q6Q1Q5	2j6p	F	75	-125,4	-138,4
PF00581	Q81UT5	3icr	A	514	-119,5	-147,3
PF00680	P12916	1xr6	A	289	-161,3	-137,3
PF00782	O95147	2wgp	A	111	-128,9	-139
PF00782	P24656	1yn9	C	119	-124,3	-148,1
PF00782	P33064	2p4d	A	110	-126,1	-142,7
PF00782	P51452	1vhr	B	124	-141,6	-143,6
PF00782	P60484	1d5r	A	124	-129,3	-126,3
PF00782	Q4QEZ7	3s4o	A	112	-131,5	-103,5
PF00782	Q66GT5	3rgo	A	102	-124,9	-103,6
PF00782	Q68J44	2y96	A	147	-134,4	-132,9
PF00782	Q8NEJ0	2esb	A	104	-138,2	-143,3
PF00782	Q9BVJ7	2img	A	95	-137,6	-146,4
PF00782	Q9NRW4	1wrm	A	88	-131,4	-143,8
PF00782	Q9UII6	2pq5	C	138	-134,9	-133,8
PF00782	Q9Y6W6	1zzw	B	408	-139,6	-119,4
PF00795	O25836	2dyv	B	166	53	-106,1
PF00795	P49954	1f89	B	469	32,4	-97,5
PF00795	P60327	1erz	A	171	43,7	-117,3
PF00795	Q44185	1fo6	C	172	47,3	-117,8
PF00795	Q82AV7	3n05	B	161	36,2	-94,6
PF00795	Q8P8M3	2,00E+011	D	143	41,8	-107,9
PF00795	Q9L543	2plq	A	166	45,6	-109,1
PF00857	Q82NB5	3kl2	D	180	-122,9	-90
PF01008	Q5JFM9	3a11	B	133	55,8	-149,6
PF01083	P00590	1cuj	A	120	54,6	-123
PF01174	P37528	2nv2	P	79	66,3	-99,6
PF01174	P83813	1q7r	A	78	54,2	-136
PF01174	Q59055	2ywj	A	75	56,5	-99,4
PF01174	Q5ND68	2abw	A	87	53,2	-109,5
PF01174	Q5SKD6	2ywd	A	81	61,2	-108,7
PF01329	P61459	1dco	G	82	-95,8	-91,1

PF01738	A6TGL0	3f67	A	120	65,3	-118,9
PF01909	P06766	2van	A	178	-105,8	-140,9
PF01909	Q5SJ64	3au2	A	186	-113,5	-138,9
PF01909	Q9UGP5	2pfn	A	415	-111	-141,3
PF01965	A9CJS5	2fex	B	101	67,8	-137,1
PF01965	B6YXG0	3l18	A	100	63,7	-120,6
PF01965	O59413	1g2i	A	100	59,7	-115,1
PF01965	P31658	1n57	A	185	65,5	-116
PF01965	P96658	3f5d	A	100	54,8	-127,8
PF01965	Q04432	1rw7	A	138	70,4	-121,9
PF01965	Q08914	3kkl	A	138	75,3	-116
PF01965	Q2GI86	3l3b	A	137	64,5	-113,8
PF01965	Q46948	2ab0	A	106	55,7	-112,2
PF01965	Q81PY3	3efe	B	110	65,3	-130,9
PF01965	Q8A8A4	3cne	D	111	52,7	-106,4
PF01965	Q99497	1pdw	H	3306	68	-108,5
PF01965	Q9HEU1	3n7t	A	141	79,7	-100,6
PF01965	Q9KPQ8	3ot1	A	108	64,8	-125,8
PF02550	Q9RM86	3gk7	B	32	57,7	-113,9
PF02574	Q93088	1lt8	A	299	-123,9	-144,2
PF02574	Q9WYA5	1q85	B	272	-114,3	-149,8
PF02774	P44801	1oza	A	136	42,8	-138,4
PF02776	P0CH62	2pgn	B	77	-8	-91,4
PF02826	P60802	3oet	G	207	-101,9	-90,4
PF02979	O66188	2dd4	L	131	-172,3	-122,6
PF03372	P00639	2a42	B	173	60,7	-129,7
PF04204	Q72X44	2ghr	A	142	44,8	-116,9
PF04273	A4Y1H6	3gxh	A	125	-145,5	-137,7
PF07722	Q92820	1l9x	B	110	53,3	-101
PF07722	Q92AL3	3fij	F	115	47,4	-108,7
PF11486	O34626	2euc	A	29	-70,9	-120,8
PF12695	Q01609	2hdw	A	179	52,7	-128,2
PF12850	Q9QZ88	1z2w	A	41	82,2	-140
PF13278	A3QGX4	3bhn	A	100	47,9	-124,8
PF13278	Q4K977	3nor	A	104	65,2	-134,3
PF13278	Q881P0	3ewn	A	154	59,8	-143
PF13278	Q97FB4	3mgk	A	102	60,1	-142,5
PF13350	A1QMT0	2oz5	A	160	-130,8	-124,5
PF13350	A3QMF6	3f41	A	548	-124,9	-134,8
PF13350	P96830	1ywf	A	160	-127,3	-126,5
