# Supplementary material for: Protein Topology Determines Cysteine Oxidation Fate: The Case of Sulfenyl Amide Formation among Protein Families
Source: PLoS Comput Biol. 2015 Mar 5;11(3):e1004051. doi: 10.1371/journal.pcbi.1004051 (PMC4351059; doi:10.1371/journal.pcbi.1004051)
Supplement: S5 Table — (PDF) [file pcbi.1004051.s016.pdf]

Table S5. Protein Crystal structures with cysteine sulfenic acid.

PDBid	Chain	ResID
3A8G	A	114
3A8H	A	114
3A8L	A	114
3A8O	A	114
3AAY	A	233
3AAY	B	233
2AF4	C	159
2AF4	D	159
2AHJ	A	114
2AHJ	C	114
4AL3	A	90
2AOT	A	217
2AOT	A	248
2AOT	B	82
2AOT	B	248
2AOU	A	217
2AOU	B	82
2AQ5	A	78
2AQ5	A	90
2AQ5	A	152
2AQ5	A	332
4ASC	A	340
4AYC	B	456
4AZ4	A	129
3B4Y	A	95
3B4Y	B	95
3B8B	A	133
3BB0	A	69
3BE9	A	89
2BFZ	B	221
2BG7	A	221
2BG7	B	221
2BG8	A	221
2BJA	A	322
2BJA	B	322
3BOO	B	1
3BQG	A	207
2BWS	A	249
2BWV	A	249
3C6B	A	60
2C9S	A	111
2C9S	F	111
2CFH	C	32
2CFH	D	32
2CIR	A	287
2CIS	A	287
3CIW	A	183
3CIW	A	322
3CIX	A	183
3CJF	A	860

3CJF	A	1005
3CJF	A	1022
3CJF	A	1043
3CJF	A	1114
3CJG	A	860
3CJG	A	1022
3CKC	A	322
3CKC	B	322
3CUS	Q	543
3CUS	R	543
3CUS	S	543
2CVO	A	145
2CVO	B	145
2CVO	C	145
2CVO	D	145
3CV2	A	438
3CV2	B	438
1CXP	C	150
1CXP	D	150
2CYZ	A	114
2CZ1	A	114
2CZ6	A	114
2CZ7	A	114
2D1Q	A	63
2D1Q	A	64
2D1R	A	63
2D1R	A	64
2D1S	A	64
2D1T	A	64
1D2V	C	150
1D2V	D	150
3D3W	A	138
2D4L	A	126
2D4M	A	126
1D5L	C	150
1D5L	D	150
1D7W	C	150
1D7W	D	150
3D7Z	A	119
3D83	A	119
3D83	A	162
2DD5	C	133
2DD5	F	133
2DD5	I	133
2DD5	L	133
4DFE	A	123
4DFE	B	123
4DFE	C	123
4DFE	D	123
4DGN	A	89
4DL1	C	150
4DL1	D	150
4DL1	G	150
4DL1	H	150

4DL1	K	150
4DL1	L	150
4DL1	O	150
4DL1	P	150
1DMP	A	67
1DMP	B	67
1DNU	C	150
1DNU	D	150
1DNW	C	150
1DNW	D	150
2DPP	A	121
3DQY	A	69
2DXB	C	133
2DXB	F	133
2DXB	I	133
2DXB	L	133
2DXB	O	133
2DXB	R	133
2DXB	U	133
2DXB	X	133
2DXC	C	133
2DXC	F	133
2DXC	I	133
2DXC	L	133
######	A	48
######	A	162
######	A	162
2EG4	B	191
2EJD	A	322
2EJD	B	322
2EJL	A	322
2EJL	B	322
4EOC	A	188
1EQ2	A	78
1EQ2	B	78
1EQ2	C	78
1EQ2	D	78
1EQ2	E	78
1EQ2	F	78
1EQ2	G	78
1EQ2	H	78
1EQ2	I	78
1EQ2	J	78
2ES4	D	92
2ES4	E	92
4ESY	B	346
4EVX	A	12
4EVX	B	12
2F2L	A	414
1F3B	A	111
1F3B	B	111
3F71	A	106
3F9P	C	150
3F9P	D	150

3FE5	A	23
3FE5	A	134
3FE5	A	182
2FHJ	D	58
2FHX	B	221
3FL5	A	89
1FNJ	A	75
3FSG	A	35
3FSG	B	35
3FSG	C	35
4FST	A	168
4FSW	A	168
4FSY	A	57
4FSZ	A	57
4FSZ	A	168
4FT3	A	168
4FT3	A	215
4FTA	A	168
4FTC	A	168
4FTN	A	168
4FTQ	A	168
1FZJ	A	121
1FZK	A	121
1FZM	A	121
1FZO	A	121
4G22	A	142
4G22	A	364
4G22	B	142
4G22	B	364
4G2M	A	364
1G55	A	140
1G55	A	287
4GH2	A	168
4GH2	A	215
1GNS	A	221
1GR7	A	26
1GR7	B	26
1GR7	C	26
1GR7	D	26
1GSN	A	63
1GSN	A	234
1GSN	A	284
1GSN	A	423
4GSB	A	63
1GZW	A	16
2HCJ	B	81
4HHC	A	669
1HIV	A	67
1HIV	B	67
1HKU	A	27
2HP0	B	271
2HPR	A	83
3HRM	A	13
3HRM	B	13

1HVR	A	67
1HVR	B	67
1I9T	A	126
2IBU	A	126
2IBU	B	126
2IBU	C	126
2IBU	D	126
2IBW	A	126
2IBW	B	126
2IBW	C	126
2IBW	D	126
2IBY	A	126
2IBY	B	126
2IBY	C	126
2IBY	D	126
3IB4	A	80
2ID4	A	190
2ID4	B	190
3IG5	A	70
3IIX	A	183
3IIX	A	322
3IIZ	A	183
3IL4	A	13
3IL4	A	22
3IL4	B	13
3IL4	B	22
3IL4	C	13
3IL4	C	22
3IL4	D	13
3IL4	D	22
3IPH	A	119
3IPH	A	162
3IQJ	A	259
3IQU	A	38
3IQV	A	38
1IRE	A	113
2ISY	A	102
2ISY	B	102
3IS2	A	71
3IV0	A	191
2J40	A	322
2J40	B	322
2J89	A	81
1JOA	A	42
3JWA	A	4
3JWB	A	4
1JZ7	A	247
1JZ7	B	247
1JZ7	C	247
1JZ7	D	247
1K3I	A	228
3K94	A	26
3K94	A	95
3K94	A	176

3K94	A	185
3K94	B	26
3K94	B	95
3K94	B	176
3K94	B	185
3KEV	A	58
3KMZ	C	2056
3KMZ	D	2056
3KXG	A	89
3KXH	A	89
3LAC	A	143
3LAC	B	143
1LEG	A	121
1LEK	A	121
1LG7	A	135
1LOQ	A	65
3LW1	A	38
3M2M	A	88
3M2M	A	130
3M2M	B	88
3M2M	B	130
3M2M	C	88
3M2M	C	130
3M2M	D	88
3M2M	D	130
3M2M	E	88
3M2M	E	130
3M2M	F	88
3M2M	F	130
3M2M	G	88
3M2M	G	130
3M2M	H	88
3M2M	H	130
1ME8	A	319
1ME9	A	319
1MEH	A	319
3MHR	A	38
3MII	A	138
3MII	B	138
1MJB	A	304
3MKJ	A	4
3MKJ	A	115
3MKJ	A	193
3MKJ	A	245
1ML6	A	111
1ML6	B	411
3N80	A	302
3N80	B	302
3N80	C	302
3N80	D	302
3N80	E	302
3N80	F	302
3N80	G	302
3N80	H	302

3NA4	A	25
3NA8	A	211
3NA8	B	211
3NA8	C	211
3NA8	D	211
3NJQ	A	16
3NJQ	B	16
3NJQ	B	179
3NON	A	101
3NON	B	104
2NQA	A	54
2NQA	A	82
2NQA	A	98
2NQA	A	191
2NQA	A	233
2NQA	B	54
2NQA	B	82
2NQA	B	98
2NQA	B	191
2NQA	B	233
1O4C	A	44
1O4F	A	44
1O4G	A	44
2O7Q	A	391
2O7Q	A	424
2O7S	A	391
2O7S	A	424
1O8V	A	63
1O9Q	A	155
1O9Q	B	155
1OET	A	215
3OP0	A	182
3OP0	A	202
3OP0	B	182
3OP0	B	202
2ORA	A	247
3OT4	A	150
3OT4	B	150
3OT4	C	150
3OT4	D	150
3OT4	E	150
3OT4	F	150
3OT4	G	150
3OT4	H	150
1OU6	A	89
1OU6	B	89
1OU6	C	89
1OU6	D	89
3OY8	A	16
3OY8	A	88
3OY8	B	16
3OYW	A	16
3OYW	A	88
3OYW	B	16

3P1N	A	38
3P1O	A	38
3P5V	A	25
3P5X	A	25
1P7T	A	617
1P7T	B	617
1P7T	B	688
3PEF	A	52
3PEF	B	52
3PEF	C	52
3PEF	D	52
3PEF	E	52
3PEF	F	52
3PEF	G	52
3PEF	H	52
4PGT	A	101
4PGT	B	101
1PKW	A	112
1PKW	B	112
1PKZ	A	112
1PKZ	B	112
1PL1	A	112
1PL1	B	112
1PL2	A	112
1PL2	B	112
1PRX	A	47
1PRX	B	47
3PR6	A	6
2PVH	A	89
2PVJ	A	89
2PVK	A	89
2PVL	A	89
2PVM	A	89
2PVN	A	89
3PVG	A	89
1Q79	A	160
2Q9J	A	73
1QBS	A	67
1QBS	B	67
2QC6	A	89
3QD5	A	76
3QD5	B	76
1QQF	A	1010
3QSB	B	260
3QSR	A	224
2QTL	A	340
2QTL	A	364
2QTL	A	421
2QTZ	A	340
2QTZ	A	421
1QVZ	A	138
1QVZ	B	138
2QX0	A	37
2QX0	B	37

3R0B	A	634
3R0B	A	669
3R0B	B	634
3R0B	C	634
3R0B	D	669
3R0B	E	634
3R0B	E	669
2RFV	A	4
2RG2	A	2
3RJT	A	18
3RJT	B	18
3ROJ	A	286
3ROJ	B	286
3ROJ	C	286
3ROJ	D	286
3RPL	B	281
3RPL	B	286
1RQ4	B	93
1RQ4	D	93
3RVA	A	230
1SBH	A	206
1SBI	A	206
3SF8	A	106
1SK4	A	300
3SS6	A	88
3SS6	B	88
3SUJ	A	63
1SVY	A	235
3T6E	M	160
3T8T	A	25
3TJJ	A	124
3TJJ	B	124
3TJJ	C	124
3TJJ	D	124
3TJJ	E	124
3TKS	A	87
3TKS	B	87
3TP1	A	44
3TPS	A	44
3TRN	A	44
3TS6	A	44
3TU8	A	94
3U11	A	586
3U11	B	586
3U2A	A	97
3U7E	B	450
3U7F	B	450
3U7H	B	450
3U9X	A	38
3UAO	A	150
3UAO	B	150
3UAO	C	150
3UAO	D	150
3UAO	E	150

3UAO	F	150
3UAO	G	150
3UAO	H	150
1UB7	A	110
1UB7	B	110
1UB7	C	110
1UB7	D	110
3UBW	A	98
1UGP	A	113
1UGR	A	113
3UU7	A	381
3UU7	A	417
3UU7	B	381
3UU7	B	417
3UU7	B	530
3UUA	A	381
3UUA	B	381
3UUA	B	530
3UUC	A	381
3UUC	B	381
3UUC	C	381
3UUC	D	381
3UUD	A	381
3UUD	A	530
3UUD	B	381
3UUD	B	417
2V3X	A	249
2V3Y	A	249
2V3Z	A	249
2V5B	A	118
2VEC	A	122
1VHQ	A	138
1VHQ	B	138
2VH3	B	65
2VL2	A	151
2VL2	B	151
2VR8	A	111
2VR8	F	111
2VRN	A	115
2VRN	B	115
2VT8	A	17
2VT8	A	36
2VT8	B	17
2VT8	B	36
2VU0	A	89
2VU0	B	89
2VU0	C	89
2VU0	D	89
2VU1	A	89
2VU1	B	89
2VU1	C	89
2VU1	D	89
1W2M	A	249
1W2M	B	249

1W2M	C	249
1W2M	D	249
1W2M	E	249
1W2M	F	249
1W6M	A	1016
1W6N	A	1016
1W6O	A	1016
1W6P	A	1016
1W6Q	A	1016
2WAW	A	81
2WEE	A	177
2WEE	B	177
2WKO	F	111
2WKT	A	89
2WKT	B	89
2WKT	D	89
1WL4	A	92
1WL5	A	92
1WL8	A	148
1WL9	A	249
2WL4	B	89
2WL4	D	89
2WL5	A	89
2WL5	B	89
2WME	C	286
2WME	E	286
1WUH	L	84
1WUH	L	546
1WUI	L	84
1WUI	L	546
1WUJ	L	546
1WUK	L	546
1WUL	L	546
2WYZ	A	111
2WYZ	F	111
2X2H	A	336
2X2H	B	336
2X2H	C	336
2X2H	D	336
2X2I	A	336
2X2I	B	336
2X2I	C	336
2X2I	D	336
2X2J	A	336
2X2J	B	336
2X2J	C	336
2X2J	D	336
1XAE	A	21
1XAE	A	47
1XAE	B	21
1XAE	B	47
1XCM	A	118
2XF1	A	34
1XVW	A	45

1XVW	B	45
2XZD	A	163
2XZD	C	163
2XZT	A	163
2XZT	C	163
2Y0B	A	163
2Y0B	C	163
2Y0M	A	316
2Y0M	A	416
1Y1F	X	336
2Y6U	A	26
2Y6U	A	127
2Y6U	A	205
2Y6U	A	298
2Y6V	A	26
2Y6V	A	127
2Y6V	A	205
2Y6V	A	298
2Y6V	B	26
2Y6V	B	127
2Y6V	B	205
2Y6V	B	298
2Y6V	C	26
2Y6V	C	127
2Y6V	C	205
2Y6V	C	298
2YES	A	177
2YES	B	177
1YJA	A	206
1YJB	A	206
1YJC	A	206
1YML	A	473
2Z2W	A	509
2ZCF	A	114
2ZCT	A	50
2ZCT	B	50
2ZCT	C	50
2ZCT	D	50
2ZCT	E	50
2ZCT	F	50
2ZCT	G	50
2ZCT	H	50
2ZCT	I	50
2ZCT	J	50
2ZPB	A	114
2ZPE	A	114
2ZPF	A	114
2ZPG	A	114
2ZPH	A	114
2ZPI	A	114
1ZSF	A	67
1ZSF	B	167
1ZSR	A	67
1ZSR	B	167

3ZS0	C	150
3ZS0	D	150
3ZS1	C	150
3ZS1	D	150
3ZVH	A	361
1ZW6	A	51
