# Supplementary material for: Protein Topology Determines Cysteine Oxidation Fate: The Case of Sulfenyl Amide Formation among Protein Families
Source: PLoS Comput Biol. 2015 Mar 5;11(3):e1004051. doi: 10.1371/journal.pcbi.1004051 (PMC4351059; doi:10.1371/journal.pcbi.1004051)
Supplement: S6 Table — Highlighted in bold are the seven families described in the main text. (PDF) [file pcbi.1004051.s017.pdf]

Table S6. HMM search results against Swiss-Prot database.

Highlighted in bold are the seven families described in the main text.

PFAM Family	Family	Number	Score	Sd Dev
**PF00102**	**Y_phosphatase**	**147**	**33.72**	**12.68**
**PF00782**	**DSPc**	**166**	**23.43**	**6.36**
PF14671	DSPn	9	22.64	1.9
PF09192	Act-Frag_cataly	1	21.5	0
**PF00117**	**GATase**	**1665**	**17.74**	**3.62**
**PF00795**	**CN_hydrolase**	**31**	**16.51**	**2.23**
PF00988	CPSase_sm_chain	1	16.3	0
**PF01174**	**SNO**	**144**	**15.8**	**4.59**
PF06418	CTP_synth_N	8	15.79	3.71
PF14566	PTPlike_phytase	5	15.5	3.44
**PF01965**	**DJ-1_PfpI**	**63**	**15.08**	**4.05**
PF07722	Peptidase_C26	12	14.95	3.26
PF06602	Myotub-related	8	14.55	1.96
PF13606	Ank_3	1	14.4	0
PF02075	RuvC	1	14.3	0
PF13507	GATase_5	156	14.21	2.35
PF00885	DMRL_synthase	3	13.8	0
**PF00581**	**Rhodanese**	**27**	**13.36**	**1.66**
PF03162	Y_phosphatase2	2	13.35	1.25
PF13292	DXP_synthase_N	2	12.95	1.25
PF01008	IF-2B	40	12.86	1.19
PF05706	CDKN3	3	12.43	0.52
PF07685	GATase_3	68	12.3	1.35
PF01255	Prenyltransf	1	12.2	0
PF04273	DUF442	1	12	0
PF00264	Tyrosinase	1	11.9	0
PF08544	GHMP_kinases_C	1	11.8	0
PF00265	TK	1	11.6	0
PF01773	Nucleos_tra2_N	1	11.5	0
PF13527	Acetyltransf_9	1	11.5	0
PF13350	Y_phosphatase3	1	11.5	0
PF01040	UbiA	1	11.4	0
PF13631	Cytochrom_B_N_2	1	11.3	0
PF00444	Ribosomal_L36	24	11.1	0.46
PF01048	PNP_UDP_1	1	11	0
PF00953	Glycos_transf_4	1	11	0
PF00089	Trypsin	1	10.7	0
PF01541	GIY-YIG	1	10.2	0
PF00083	Sugar_tr	1	9.7	0
