# Supplementary material for: Protein Topology Determines Cysteine Oxidation Fate: The Case of Sulfenyl Amide Formation among Protein Families
Source: PLoS Comput Biol. 2015 Mar 5;11(3):e1004051. doi: 10.1371/journal.pcbi.1004051 (PMC4351059; doi:10.1371/journal.pcbi.1004051)
Supplement: S7 Table — (PDF) [file pcbi.1004051.s018.pdf]

Table S7 .Structural and energetic parameters for the cyclic sulfenyl amide formation in water

Parameter	Peptide in water		
ΔE	-1.5 kcal/mol		
Eact	9 kcal/mol		
	Reactive	TS	Product
N-CA-C-N+1	-155	-154	-109
dSO	1.73	2.23	2.4
dNH	1.03	1.58	2.63
dSN	2.81	1.92	1.83
$dH_{NH}O_W$	1.83	1.06	1.02
qS	0.012	0.018	-0.16
qOH	-0.158	-0.032	-0.028
qH_{NH}	-0.16	-0.044	0.002
qN	0.354	0.126	0.156
